# Pharmacological treatments in disorders of consciousness

**DOI:** 10.1093/braincomms/fcag369

**Published:** 2026-10-06

**Authors:** Christian Saleh, Fatemeh Seyfzadeh, Emilie Szymkowicz, Naji Alnagger, Tatiani Soultana Ilia, Alexandra A Oancea, Olivia Gosseries

**Affiliations:** University of Basel, 4001 Basel, Switzerland; Coma Science Group, GIGA-Consciousness, GIGA Institute, University of Liège, 4000 Liège, Belgium; NeuroRehab & Consciousness Clinic, Neurology Department, University Hospital of Liège, 4000 Liège, Belgium; NeuroRecovery Lab, GIGA-Consciousness, GIGA Institute, University of Liège, 4000 Liège, Belgium; Coma Science Group, GIGA-Consciousness, GIGA Institute, University of Liège, 4000 Liège, Belgium; NeuroRehab & Consciousness Clinic, Neurology Department, University Hospital of Liège, 4000 Liège, Belgium; Coma Science Group, GIGA-Consciousness, GIGA Institute, University of Liège, 4000 Liège, Belgium; NeuroRehab & Consciousness Clinic, Neurology Department, University Hospital of Liège, 4000 Liège, Belgium; Division of Neuropediatrics, Development and Rehabilitation, Department of Pediatrics, Inselspital, Bern University Hospital, University of Bern, 3010 Bern, Switzerland; Department of Neurology, University Hospital of Basel, 4031 Basel, Switzerland; Coma Science Group, GIGA-Consciousness, GIGA Institute, University of Liège, 4000 Liège, Belgium; NeuroRehab & Consciousness Clinic, Neurology Department, University Hospital of Liège, 4000 Liège, Belgium

**Keywords:** coma, unresponsive wakefulness syndrome, vegetative state, minimally conscious state, pharmacology

## Abstract

Disorders of consciousness (DoC) are among the most severe and devastating neurological conditions. The poorly understood underlying pathophysiology of DoC renders the search for adequate treatment options particularly difficult. The aim of this narrative review is to provide an overview of currently available pharmacological treatments, report the limitations of current research and discuss future directions, notably potential new treatments, advances in modelling approaches, potential mechanisms of action and the development of a standardized registry. We review pharmacological treatments that promote the recovery of consciousness in patients with DoC acting on dopaminergic (amantadine, levodopa, apomorphine, methylphenidate, bromocriptine, modafinil and selegiline) and GABAergic (zolpidem, baclofen and midazolam) pathways, as well as other pharmacological treatments (tricyclic antidepressants, anticonvulsants and simvastatin) and the combination of different treatments. The literature includes a limited number of randomized controlled trials (RCTs) alongside a large body of retrospective studies and case reports, which are classified as class IV evidence. Reported improvements in arousal or responsiveness are inconsistent, reflecting marked heterogeneity in patient populations, dosing regimens and outcome measures. Risk-of-bias assessment indicated that 12 of 22 RCTs (∼55%) exhibit some concerns or a high risk of bias. Multiple correspondence analysis yielded a descriptive mapping of RCT characteristics, demonstrating how pharmacological trials in DoC are grouped. Although mechanistic hypotheses converge on dopaminergic, GABAergic and thalamocortical modulation, the current literature on pharmacological treatments in DoC remains poorly reproducible. Given the significant methodological variability (e.g. study design, patient characteristics and outcome measures) across studies, current evidence does not allow firm conclusions regarding the most effective consciousness-enhancing pharmacological treatments in DoC. To make informed decisions in terms of pharmacological treatments and move towards personalized care for patients with DoC, rigorously designed multicentre controlled trials with harmonized outcome measures conducted with mechanistic and computational approaches are required. This can be achieved through an international registry to standardize data collection and improve reproducibility and replicability.

## Teaser

Saleh *et al*. reviewed the literature on pharmacological treatments for disorders of consciousness, with qualitative and quantitative analyses of randomized controlled trials. Given the limited and heterogeneous evidence, they recommended standardized methods, mechanistic studies, investigation of novel therapeutic agents and establishment of a shared international registry to guide future practice.

## Graphical Abstract

**Graphical Abstract:**
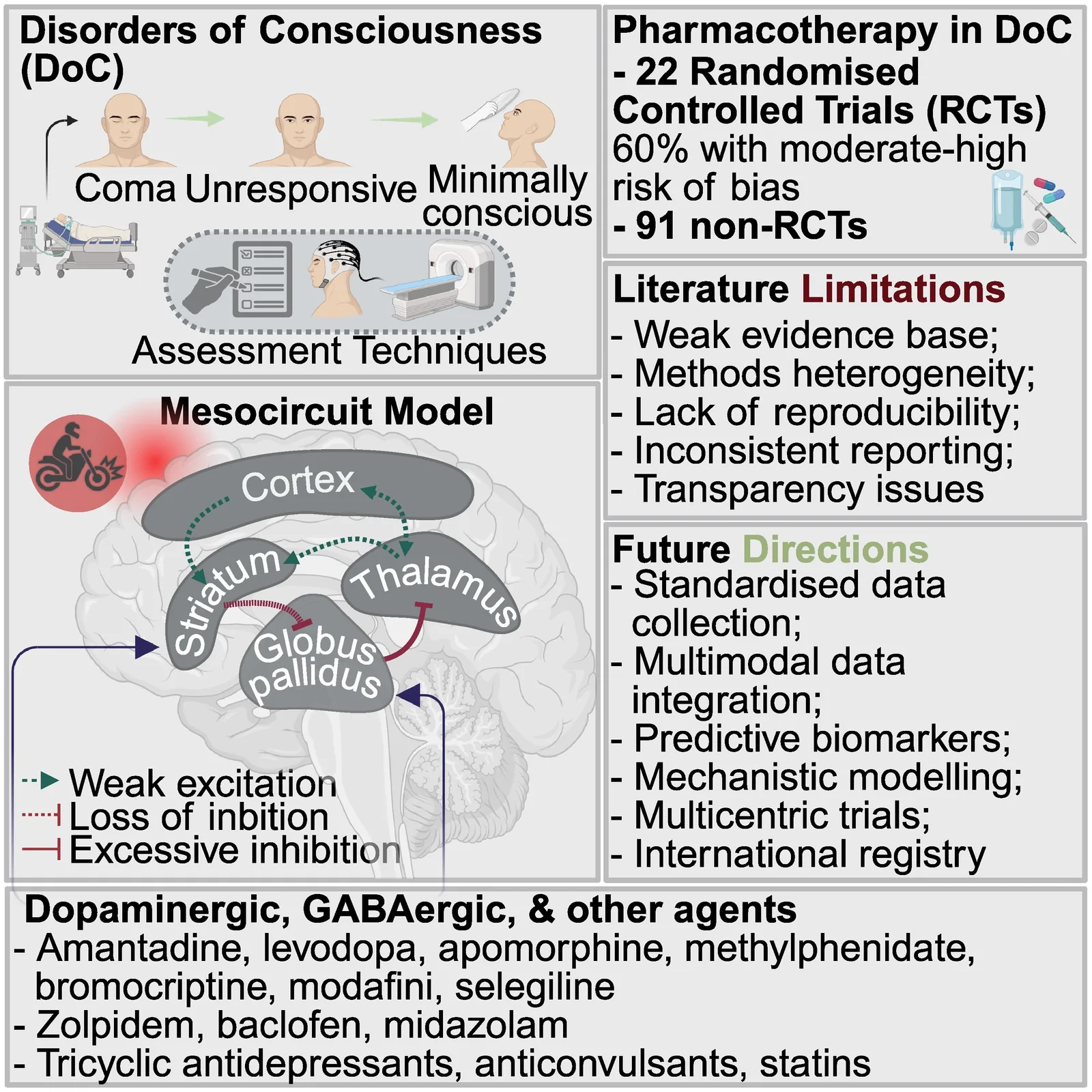

## Introduction

Disorders of consciousness (DoC) are caused by severe acquired brain injuries, including traumatic brain injuries (TBIs), ischaemia and hypoxia, which lead to severe disruption of neuronal networks.^1-3^ DoC are viewed on a continuum of states, in which the comatose state is defined by an absence of wakefulness (no eye opening), with only reflex movements.^3^ Recovery of wakefulness (i.e. spontaneous or induced eye opening) without signs of awareness leads to the unresponsive wakefulness syndrome (UWS), also referred to as the vegetative state (VS).^4,5^ Recovery of awareness leads to the minimally conscious state (MCS), which is defined as ‘a condition of severely altered consciousness in which minimal but definite behavioural evidence of self or environmental awareness is demonstrated’.^6,7^ The clinical characteristics of the MCS include, among others, consistent or reproducible responses to command, visual pursuit and fixation, automatic motor responses, and localization of nociceptive stimulations.^8^ Recent works have suggested that other behaviours, such as resistance to eye opening or habituation to auditory startle reflex, should be considered as potential indicators of consciousness that denote the MCS.^9-11^ Depending on the presence or absence of language, the MCS has been categorized into MCS plus and MCS minus, respectively.^12^ Once patients can functionally communicate or use objects, they are considered to have emerged from the MCS. Spontaneous recovery from DoC may occur on average within the first 6–12 months or even years later.^13^

To diagnose these different clinical entities,^1^ several behavioural scales have been developed in the last two decades. A summary of the currently available diagnostic tools to assess DoC patients’ state of consciousness is presented in Supplementary Box 1, given that accurate diagnosis is essential not only for clinical management and prognostication but also for guiding therapeutic interventions and evaluating treatment efficacy.

Building on this diagnostic framework, in this narrative review, we provide an overview of the currently available pharmacological treatments for recovery from DoC, including dopaminergic (amantadine, levodopa, apomorphine, methylphenidate, bromocriptine, modafinil and selegiline) and GABAergic (zolpidem, baclofen and midazolam) agents, as well as other pharmacological agents, such as tricyclic antidepressants, anticonvulsants, simvastatin and combined treatments. We highlight the limitations of current literature that hinder translation of scientific findings into clinical practice and complement the descriptive synthesis of the studies with both qualitative and quantitative analyses [risk-of-bias (RoB) assessment and multiple correspondence analysis (MCA)] of randomized controlled trials (RCTs). Together with a brief mechanistic overview derived from prior research, we also outline key future directions, including testing potential new treatments as well as advanced modelling approaches and further clarifying mechanisms of pharmacological neuromodulation. We also emphasize the need for a standardized multicentric registry of DoC patients treated with consciousness-enhancing drugs to facilitate large-scale, generalizable clinical research. Overall, this work aims to guide high-quality future studies and support evidence-based personalized care for patients with DoC.

## Materials and methods

### Literature search

A non-systematic literature search was conducted on PubMed and Scopus databases to identify studies on pharmacological interventions in patients with DoC published before December 2025. The search strategy combined controlled vocabulary (e.g. MeSH terms on PubMed) and free-text terms related to DoC and pharmacological treatments (see Supplementary material). In addition to the electronic database search, the reference lists of all eligible articles were manually screened to identify further relevant publications not captured through the initial search. The search was limited to human studies and excluded reviews, editorials and conference abstracts.

### Eligibility criteria and study selection

Studies were included if they met the following criteria: (i) primary research articles with empirical data on pharmacological treatments for patients with DoC, and (ii) published in English or French. Both paediatric and adult patient populations were considered eligible to provide a comprehensive overview of pharmacological evidence in different clinical contexts. The selection process consisted of three steps: (i) title screening, (ii) abstract screening and (iii) full-text review, when necessary, to confirm eligibility. Retrieved records were screened by two authors, and relevant studies were retained for full-text review. Discrepancies were resolved through discussion.

### Data extraction and synthesis

Data extraction was performed by two authors and verified by one of the authors to ensure accuracy and consistency. The following data were extracted from each included study: pharmacological treatment(s) (drug class, dose and duration of treatment), study design, sample size, patients’ characteristics (sex, age, brain injury aetiology, time since injury and diagnosis/state of consciousness at baseline), outcome measures and outcomes. A descriptive overview of all included studies was conducted to summarize methodological features and general outcomes. The level of evidence (LoE) of each study was assessed based on the Oxford Centre for Evidence-Based Medicine (OCEBM) framework (Table 1, Supplementary Tables 1 and 2).^36^ Further qualitative and quantitative analyses were conducted exclusively on RCTs to illustrate their methodological quality and heterogeneity. This decision was made because RCTs represent the highest LoE among the available study designs and offer the methodological rigour needed to support reliable comparative analyses.

**Table 1:** Overview of RCTs included in this narrative review

Agent	Authors (year)	Design/LoE	No. of patients	Aetiology	Main outcome(s)
Dopaminergic					
AM	Coppler *et al*. (2024)^14^	Double-blind/Level 1b	14 (10 m)	Non-TBI	No significant between-group differences
	Gatto *et al*. (2024)^15^	Double-blind/Level 1b	37 (13 m)	Non-TBI	No significant between-group differences
	Shimia *et al*. (2023)^16^	Triple-blind/Level 1b	57 (45 m)	TBI	Greater change from baseline in DRS scores in the amantadine group
	Shafiee *et al*. (2022)^17^	Single-blind/Level 2	66 (45 m)	TBI	Greater changes in GCS scores from admission to day 11 in the amantadine group (107%) compared to zolpidem (58%) and placebo (31.5%)
	Abbasivash *et al*. (2019)^18^	Double-blind/Level 1b	66 (46 m)	TBI	No significant between-group differences
	Akcil *et al*. (2018)^19^	Double-blind/Level 2	12 (7 m)	Non-TBI	Higher CRS-R and lower DRS scores on day 5, and at 3 and 6 months in both groups versus baseline, and in the amantadine group versus placebo
	Ghalaenovi *et al*. (2018)^20^	Double-blind/Level 1b	40 (37 m)	TBI	Greater GCS score increases from day 1 to day 7 in the amantadine group
	Giacino *et al*. (2012)^21^	Double-blind/Level 1b	184 (133 m)	TBI	Faster recovery in the amantadine group, compared to placebo
	McMahon *et al*. (2009)^22^	Double-blind crossover/Level 1	7 (6 m)	TBI	Physicians reporting greater improvements in consciousness during amantadine
	Patrick *et al*. (2006)^23^	Double-blind/Level 1b	10 (4 m)	TBI	No significant efficacy differences between amantadine and pramipexole
	Meythaler *et al*. (2002)^24^	Double-blind crossover/Level 1	35 (26 m)	TBI	Faster recovery during amantadine in both groups
AP	Othman *et al*. (2025)^25^	Double-blind crossover/Level 1b	50 (36 m)	Mixed	Significantly increased probability of improved arousal after methylphenidate No significant effect of apomorphine or methylphenidate on pupillary responses
ME	Othman *et al*. (2025)^25^	See above	See above	See above	See above
	Moein *et al*. (2006)^26^	Double-blind/Level 1b	80 (66 m)	TBI	Shorter ICU and hospital stay in severe TBI
MO	Talebi Kiasari *et al*. (2024)^27^	Double-blind/Level 1b	18 (9 m)	Non-TBI	No significant between-group differences
	Zand *et al*. (2024)^28^	Double-blind/Level 1b	85 (73 m)	TBI	Higher proportion of patients achieving clinically meaningful improvement with modafinil versus placebo (54% and 56% versus 32% and 34%)
▪ GABAergic					
ZO	Rahat Dahmardeh *et al*. (2024)^29^	Double-blind/Level 1b	80 (55 m)	Mixed	No significant between-group differences
	Shafiee *et al*. (2022)^17^	See above	See above	See above	See above
	Chatelle *et al*. (2014)^30^	Double-blind crossover/Level 1b	3 (1 m)	Non-TBI	Emergence from MCS and increases in metabolism after zolpidem versus placebo
	Machado *et al*. (2014)^31^	Double-blind crossover/Level 1b	8 (6 m) 8 controls	Mixed	Generalized neurobehavioural and EEG activation and autonomic shift toward sympathetic dominance with preserved physiological stability after zolpidem
	Whyte *et al*. (2014)^32^	Double-blind crossover/Level 1	84 (57 m)	Mixed	Increased movement, social interaction, command following, communication and functional object use in 8 male patients (∼5%)
	Whyte and Myers (2009)^33^	Double-blind, crossover/Level 1	15 (10 m)	Mixed	1/15 patients (∼7%) showing a clinically significant response within 30 minutes of zolpidem administration with a change of diagnosis from VS to MCS
	Brefel-Courbon *et al*. (2007)^34^	Double-blind crossover/Level 4	1 (f)	Non-TBI	Improved motor performance, language-related functions, cerebral metabolism and regional blood flow, compared to placebo
Other					
SI	Shafiee *et al*. (2021)^35^	Double-blind/Level 1	98 (66 m)	TBI	More conscious patients at discharge and 1 month after in the simvastatin group

Detailed information on RCTs in Table 1 is available in Supplementary Table 1. Studies with other designs (e.g. case reports) are presented in Supplementary Table 2.

*Aetiology*: TBI: traumatic brain injury. *Diagnosis*: MCS: minimally conscious state; VS: vegetative state. *Behavioural measures*: CRS-R: Coma Recovery Scale-Revised; DRS: Disability Rating Scale; GCS: Glasgow Coma Scale. *Pharmacological agents*: AM: amantadine; AP: apomorphine; ME: methylphenidate; MO: modafinil; ZO: zolpidem; SI: simvastatin. *Neuroimaging measures*: EEG: electroencephalography. *Other*: f: female; ICU: intensive care unit; LoE: level of evidence; m: male; RCT: randomized controlled trial.

### Risk-of-bias assessment

The methodological quality of the included RCTs was assessed using ‘RoB 2: A Revised Cochrane Risk-of-Bias Tool for Randomised Trials’.^37^ Each RCT was evaluated in terms of bias arising from the following domains: D1: randomization process, D2: deviations from intended interventions, D3: missing outcome data, D4: measurement of the outcome, and D5: selection of the reported result. For crossover RCTs, an additional domain (DS) assessing bias arising from period and carryover effects was included. Each domain was rated as ‘low risk of bias,’ ‘some concerns’ or ‘high risk of bias’. The overall RoB was also determined for each study following the Cochrane guidelines.^37^

### Multiple correspondence analysis

MCA was performed to explore associations among categorical RCT characteristics. MCA is a factorial method that generalizes correspondence analysis to multiple qualitative variables.^38-40^ In this review, 22 RCTs were studied based on the following qualitative characteristics: pharmacological treatment, study design (parallel and crossover), blinding (no blinding, single-blind, double-blind and triple-blind), patients’ sex proportion (male-dominant, female-dominant and balanced sex) and age category (child/adolescent, young adult, middle-aged and mixed age), aetiology (TBI, non-TBI, and mixed aetiology), assessment techniques [clinical only (e.g. intensive care unit {ICU}/hospital stay and duration of mechanical ventilation), neurobehavioural (e.g. Coma Recovery Scale-Revised {CRS-R} and Glasgow Coma Scale {GCS}), neurobehavioural and clinical, neurobehavioural and imaging (e.g. positron emission tomography {PET})], sample size (*N* ≤ 20: small, 20 < *N* < 50: medium and *N* ≥ 50: large), treatment administration (single or multiple administration) and RoB (low, some concerns, or high) (see Supplementary Tables 3 and 4).

These study characteristics were selected for the MCA based on their relevance to heterogeneity across RCTs. Patient-related variables (age, sex and aetiology) have been widely discussed as factors influencing recovery and treatment response in DoC which may also affect the selection and interpretation of outcome measures.^1,41-44^ Methodological variables (study design, blinding, and sample size) were selected due to their impact on the reliability and interpretability of findings.^45-47^ Given the importance of selecting appropriate outcome measures in clinical studies,^48^ outcome measures were incorporated to capture how consciousness and treatment effects were evaluated across RCTs. Treatment administration frequency was included to account for differences in intervention delivery that may influence observed effects. RoB was included as an integrative measure of study quality. With these characteristics, the MCA provides an overview of how RCTs on pharmacotherapy in DoC methodologically differ, offering a framework for interpreting heterogeneity across studies. The analysis was conducted in R (version 4.4.0). To ensure transparency and reproducibility, Supplementary Tables 5–9 provide detailed numerical outputs of the MCA (for more detailed information, see Supplementary material).

## Neuropharmacology studies

The literature on pharmacological treatments for DoC is presented below and summarized in Fig. 1. Table 1 provides a brief overview of RCTs (*n* = 22), Supplementary Table 1 presents a more detailed overview of RCTs, and Supplementary Table 2 reports studies with other designs (*n* = 91).

**Figure 1:**
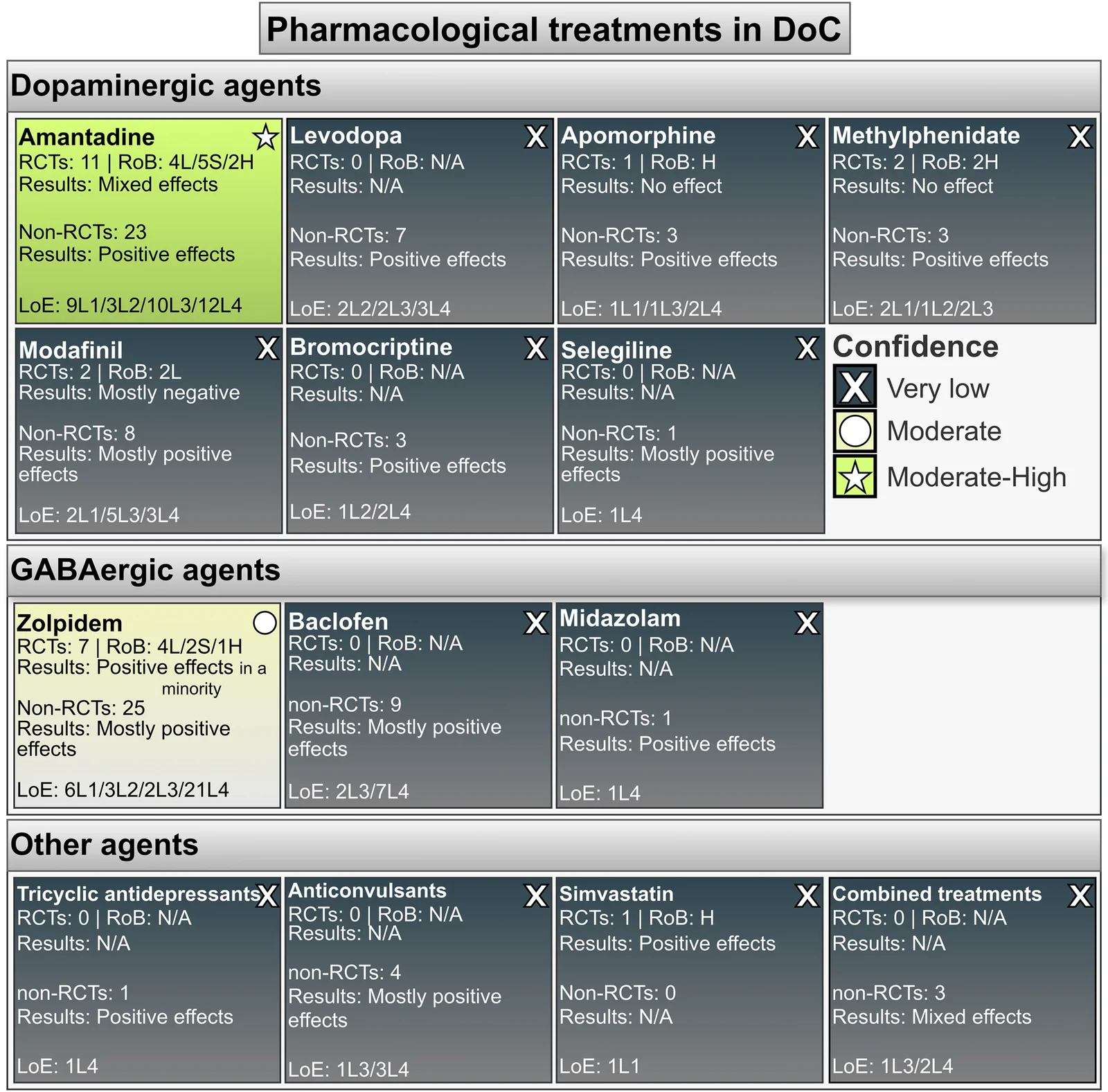
**Summary of evidence on pharmacological treatments in disorders of consciousness (DoC).** Among dopaminergic agents, amantadine presents the strongest evidence with 11 randomized controlled trials (RCTs) and 23 non-RCTs, showing mixed but generally positive outcomes and thus a moderate to high level of confidence. Among GABAergic agents, the evidence on zolpidem represents a moderate level of confidence with 7 RCTs and 25 non-RCTs, showing positive outcomes in a minority of patients. The level of evidence for other pharmacological treatments remains very low, despite some positive outcomes. RoB: risk of bias; L: low; S: some concerns; H: high; LoE: level of evidence; L1/2/3/4: Level 1/2/3/4. Colours and symbols represent the level of confidence based on the LoE.

### Dopaminergic agonists/antagonists

Dopaminergic agents modulate specific receptors and/or neurotransmitters in the dopamine pathways through upregulation (i.e. agonist) or downregulation/blockade (i.e. antagonist). They have emerged as a key target for the treatment of DoC.^49,50^

#### Amantadine

Amantadine, an adamantane derivative, is an indirect dopamine agonist and a non-competitive *N*-methyl-D-aspartate receptor antagonist with neurogenic anti-inflammatory effects.^51^ The Food and Drug Administration (FDA)-approved indications for amantadine include Parkinson's disease, drug-induced extrapyramidal symptoms, and influenza. Based on a large RCT, the American clinical practice guidelines (2018) provided a level B recommendation for the administration of amantadine (100–200 mg twice daily) to VS/UWS or MCS adult patients with traumatic DoC between 4 and 16 weeks post-injury.^1,21^

Thirty-four studies (11 RCTs, 1 alternate-treatment baseline-controlled trial, 2 single-case experimental studies, 2 observational cohort studies, 12 retrospective studies and 6 case reports/series) have been published on amantadine (50–400 mg or 4–6 mg/kg daily for paediatric patients and 50–600 mg daily for adult patients) in 1235 acute and prolonged DoC patients (including 24 paediatric (<18 years old) patients, setting not systematically reported, mixed aetiologies). In an RCT, Giacino and colleagues (2012)^21^ reported significantly faster functional recovery with amantadine compared to placebo in 184 TBI patients, as measured by the Disability Rating Scale (DRS). Further RCTs (including paediatric patients) have also reported significantly increased CRS-R, DRS, GCS, Coma/Near coma Scale (CNCS), Glasgow Outcome Scale (GOS), Western Neuro Sensory Stimulation Profile (WNSSP), Mini-Mental Status Examination (MMSE), and Functional Independence Measure (FIM-cog) scores with amantadine.^16,17,19,20,23,24,52^ However, positive behavioural outcomes were not necessarily associated with favourable clinical outcomes (e.g. duration of mechanical ventilation, length of hospitalization and/or mortality).^17,18^ Non-significant increases in CRS-R, GCS and GOS scores were also reported with amantadine in several studies,^17,18,22,53,54^ including RCTs.^14,15^

Other non-RCT studies have reported positive behavioural, neurophysiological and neuroimaging responses to amantadine only,^23,55-59^ as well as amantadine in combination with other treatments (see Combination of Agents section).^52-54,56,58,60-73^ From a neural perspective, amantadine has been associated with higher levels of anterior forebrain corticothalamic integrity and increased predominant background alpha activity.^62,69^ Functional magnetic resonance imaging (fMRI) showed increased activation of brain areas corresponding to task instructions after 3 months of treatment and metabolic changes in a widespread fronto-temporo-parietal network and the sensorimotor area.^71,73^

Reported adverse effects of amantadine in DoC patients include epileptic seizures, nausea, vomiting, elevated liver function, dizziness and intermittent tremor.^20,22,52^

#### Levodopa

Levodopa, a synthetic dopamine precursor, is converted to dopamine by L-aromatic amino acid decarboxylase. To prevent levodopa conversion to dopamine in peripheral tissues, a peripheral decarboxylase inhibitor, such as carbidopa or benserazide, is often combined with levodopa.^74^ Approved by the FDA for the treatment of Parkinson’s disease, levodopa has been used as an off-label treatment for DoC.

Seven studies (two prospective studies, one retrospective study and four case reports/series) have been conducted so far with levodopa (100 mg with carbidopa 25 mg or 2 mg/kg daily for paediatric patients and 200 mg with carbidopa 50 mg daily to 750 mg with carbidopa 75 mg daily for adult patients) in 30 acute and prolonged DoC patients (including five paediatric patients, setting not systematically reported, mixed aetiology). Improvements such as alertness and wakefulness, language-related functions, communication, self-feeding, supported walking, CRS-R diagnosis and increased WNSSP scores have been reported, along with the electroencephalography (EEG) showing a progressive replacement of delta and theta waves by alpha waves.^52,75-80^ Criteria for recommending levodopa after severe brain injury are: a diagnosis of UWS or MCS, concomitant symptoms of Parkinsonism (particularly with unilateral predominant rigidity/spasticity and akinesia), and concomitant neuroradiological findings of high-intensity lesions in the dopaminergic pathway on T2-weighted MRI, particularly in the acute stage.^77^

Reported levodopa-induced adverse effects are limited to visual hallucinations in one DoC patient at a high daily dose of 750 mg, which were resolved once the dose was reduced.^75^

#### Apomorphine

Apomorphine, an aporphine derivative of the dibenzoquinoline class, acts as a potent direct non-selective dopamine receptor agonist with strong affinity for D2 receptors (particularly concentrated in the striatum) and a serotonin and α-adrenergic receptor agonist.^81^ Approved by the FDA for the treatment of Parkinson’s disease, apomorphine has been investigated off-label for the treatment of DoC.

Four studies (one crossover RCT, one open-label controlled study, one open-label pilot study, and one case report) have been conducted on 65 acute and prolonged DoC patients (continuous daily subcutaneous infusions of up to 8 mg/h for 12 h, mixed aetiology). The RCT on apomorphine showed no significant superiority of apomorphine over methylphenidate or placebo, with Full Outline of Responsiveness (FOUR), Simplified Evaluation of Consciousness Disorders (SECONDs), or automated pupillometry assessments in the acute setting.^25^ Nonetheless, in non-RCT studies, change of CRS-R diagnosis and new language-related behaviours were among the positive outcomes.^82^ Long-term increases in CNCS, DRS and GOS-E scores were also observed up to two years post-treatment.^83,84^ From a neural perspective, EEG findings indicated increased whole-brain and alpha-band parietotemporal connectivity as well as increased whole-brain and frontal alpha-band network participation coefficient following apomorphine treatment, along with increased global metabolism observed in PET.^82^

Reported adverse effects of apomorphine in DoC include vomiting and nausea (preventable with concomitant antiemetic treatment), cutaneous inflammation, mild eosinophilia, agitation/tachycardia, sedation/drowsiness, sleeping disturbances and mild dyskinesia.^83,84^

#### Methylphenidate

Methylphenidate, a piperidine derivative, is a central nervous system stimulant (pharmacologically distinct from amphetamine) that acts as an indirect dopamine and norepinephrine agonist.^85^ Approved by the FDA for the treatment of attention-deficit hyperactivity disorder and narcolepsy, methylphenidate has been used off-label for the treatment of DoC.

Five studies (one crossover and one parallel RCT, one prospective study and two retrospective studies) have been conducted with methylphenidate (10–80 mg daily or 0.3 mg/kg/day twice daily) in a total of 119 acute and prolonged DoC patients (in ICU and neurorehabilitation settings, including at least three paediatric patients, mixed aetiologies). The crossover RCT results indicated a higher probability of arousal after methylphenidate, but no clinical superiority to apomorphine or placebo was observed. The other RCT did not report any significant changes in GCS.^26^

In non-RCT studies, improved GCS, modified Rankin Scale (mRS) and WNSSP scores were reported along with command-following, higher survival rates to hospital discharge, and Cerebral Performance Categories score (in a cohort treated with methylphenidate, among other neurostimulants).^52,66,86^ From a neural perspective, increased cerebral glucose metabolism was observed in the precuneus, occipital gyri, and frontal gyrus after methylphenidate.^86^

Reported adverse effects in DoC include paroxysmal or ventricular tachycardia.^66^

#### Bromocriptine

Bromocriptine, a semisynthetic ergot derivative agent, acts as a sympatholytic dopamine D2 and serotonin receptor agonist but also as a partial dopamine D1 receptor agonist, dopamine D4 receptor antagonist, and glutamate release inhibitor.^87^ Approved by the FDA for the treatment of Parkinson's disease, acromegaly, pituitary prolactinomas, and type 2 diabetes, bromocriptine has also been used off-label for the treatment of DoC.

Three studies (one prospective cohort study and two retrospective studies) have been conducted with bromocriptine (0.125–20 mg daily for paediatric patients and 5–25 mg daily for adult patients) in a total of 42 DoC patients in neurorehabilitation (including seven paediatric patients, TBI aetiology). Increased GCS, GOS, CRS-R scores (with diagnostic category transition), DRS scores (up to 1 year post-treatment) and WNSSP scores were reported.^3,52,88^

No adverse effects were reported in patients with DoC.

#### Modafinil

Modafinil, a synthetic eugeroic, is an arousal-promoting agent (pharmacologically distinct from central nervous system stimulants such as methylphenidate and amphetamines), stimulating dopamine but also histamine, noradrenaline, serotonin and orexin neurotransmitters with antioxidant effects.^89-91^ Approved by the FDA for the treatment of sleep disorders, modafinil has also been investigated off-label for the treatment of DoC.

Ten studies (two parallel RCTs, five retrospective studies, and three case reports) have been conducted with modafinil (50–400 mg daily) in a total of 232 ICU and neurorehabilitation DoC patients (including one paediatric patient, mixed aetiologies). One RCT in patients with non-TBI aetiology was not indicative of superiority of modafinil to placebo in GCS, blood markers, or mortality rates.^27^ Results of the other RCT in TBI patients aligned with previous results in terms of length of ICU/hospital stay, duration of mechanical ventilation, number of ventilator-free days, duration of intubation, tracheostomy rates or mortality. The latter, however, showed that with modafinil, a higher proportion of patients achieved a clinically meaningful increase in GCS motor scores.^28^ Non-RCT studies reported improved Wessex Head Injury Matrix (WHIM), GCS, mRS, DRS, CNCS and Levels of Cognitive Functioning Assessment Scale (LOCFAS) scores along with a higher probability of favourable discharge, shorter hospital stay and partial restoration of circadian rhythm.^53,63,79,92-96^

Reported adverse effects of modafinil in the DoC population included agitation, insomnia, sedation, intermittent tremor and dizziness.^23,53,63,79,92-96^

#### Selegiline

Selegiline, an irreversible selective monoamine oxidase B (MAO-B) inhibitor, presents dopaminergic and neuroprotective effects.^97^ Approved by the FDA for the treatment of depression and Parkinson’s disease, selegiline (5–10 mg daily) has been investigated off-label in a single open pilot study in 10 UWS and MCS patients in neurorehabilitation (mixed aetiologies), reporting improved CRS-R diagnosis and arousal level.^98^

The reported adverse effects of selegiline in DoC include tachycardia and persistent diarrhoea.^98^

### Summary of the level of evidence on dopaminergic agents

The LoE for dopaminergic agents in DoC is heterogeneous, with amantadine dominating, supported by moderate to high-quality evidence from multiple RCTs and guideline recommendations.^1,21^ While some variability and non-significant findings exist,^14,15,17,18,22,53,54^ the overall body of evidence indicates reproducible awareness-enhancing effects,^16-20,23,24,52-58,60-73^ supporting moderate to high confidence in benefit and justifying it as the standard of care for TBI-induced DoC at 4–16 weeks post-injury.^1,21^

For levodopa, bromocriptine and selegiline, the LoE is very low with uncontrolled studies and case reports.^75-79^ The high-level evidence on apomorphine,^25^ methylphenidate^25^ and modafinil^27,28^ remains limited and inconsistent. Notably, the majority of RCTs (9/15) present a moderate to high RoB (see Risk-of-Bias Assessment section). As such, this evidence should be considered hypothesis-generating rather than confirmatory, and these agents remain experimental and require further investigations in larger methodologically rigorous studies.

### Gamma-aminobutyric acid agonists

Gamma-aminobutyric acid (GABA) is a primary inhibitory neurotransmitter that inhibits neural transmission, leading to reduced neuronal excitability.^99^ The frequently used GABAergic treatments for DoC are zolpidem, baclofen and midazolam.

#### Zolpidem

Zolpidem is a highly selective non-benzodiazepine hypnotic that acts as a positive allosteric modulator of GABA-A receptors. Approved by the FDA for the treatment of insomnia, zolpidem has been investigated off-label for the treatment of DoC. Initially prescribed to address restlessness, zolpidem was accidentally discovered to induce rapid transient paradoxically improved functional communication in an allegedly UWS patient.^100^

Thirty-two studies have been conducted (2 parallel and 5 crossover RCTs, 1 single-patient placebo-controlled trial, 1 single-case experimental study, 1 open-label study, 4 prospective studies, 1 retrospective study and 17 case reports/series) on the effects of zolpidem (2.5–30 mg/day, single or repeated administrations) in 532 adult and 3 paediatric patients (acute and prolonged, mixed aetiologies). Approximately 5–20% of DoC patients, irrespective of aetiology, have responded paradoxically to zolpidem and recovered signs of consciousness,^32,33,101,102^ such as walking, verbalizing and swallowing food. These ‘awakening effects’ can, however, be present with or without a change of diagnosis or behavioural responses,^33,102-104^ mostly assessed using the CRS-R, GCS, MMSE, RASS, paediatric FIM, WHIM and MATADoC scales.^31,103,105-112^

Across RCTs, one parallel trial found no significant differences between zolpidem and control in GCS, duration of mechanical ventilation, length of ICU/hospital stay, mortality, or GCS scores across aetiologies.^29^ In a three-arm parallel trial, no between-group differences were found in mortality, mechanical ventilation duration or hospitalization length, yet the zolpidem group showed an upward trend in GCS scores although changes were greater with amantadine.^17^ Crossover RCTs identified a small subset of zolpidem responders with clinically meaningful behavioural changes, including emergence from MCS, command following, and improved motor performance while no such effects were observed after placebo.^30,32,33^ From a neural perspective, post-zolpidem MRI and PET in zolpidem responders demonstrated increased frontal activation, mainly in bilateral superior frontal gyri and medial frontal cortex, and increased blood flow in anterior cingulate and orbitofrontal regions.^30,34^

Non-RCT studies in paradoxical responders reported, among others, recovery of command following, verbalization, functional communication and object use, and motor functions.^107,108,111-118^ Neurobehavioural evaluations have been mostly performed in parallel with neurophysiological or neuroimaging assessments, including EEG, functional near-infrared spectroscopy, magnetoencephalography, MR, single-photon emission computed tomography, PET and/or cerebral state monitoring.^31,103-106,109-112,119-123^ From a neural perspective, paradoxical responders have shown the suppression of pathological slow oscillations and a shift from theta to alpha and beta bands.^31,32,103,104,119,120^ Similar post-zolpidem shift from slow to fast oscillations was also observed with magnetoencephalography.^121^ Resting-state functional near-infrared spectroscopy in a responder with TBI was indicative of higher frontal connectivity, compared to pre-medication parietal connectivity.^111^ Paradoxical responders have also had increased cerebral metabolism, perfusion and blood oxygenation,^100,110,124,125^ yet another study on TBI patients showed no significant difference in cerebral perfusion, assessed with SPECT, between paradoxical and non-paradoxical responders.^126^

The reported adverse effects of zolpidem in patients with DoC are sedation and restless movements.^32,127^ However, several studies have reported no adverse effects.^102,105,106,114^

#### Baclofen

Baclofen is a selective GABA-B receptor agonist administered orally or intrathecally as a muscle relaxant in multiple sclerosis, spinal cord injury, and DoC.^128-131^ Although primarily used to manage severe spasticity, intrathecal baclofen (50–800 mcg/day) has been shown to improve consciousness and decrease spasticity, dysautonomia and hypertonia in nine studies (one open-label study, two retrospective studies and six case reports/series) in a total of 100 DoC patients (including at least one paediatric patient, mixed aetiologies).^131-139^

Intrathecal baclofen led to behavioural responsiveness in VS patients while oral baclofen had failed.^131^ Consciousness was mostly assessed with the CRS-R, but also with the GCS.^132,133,135,140^ Spasticity and pain were assessed with the Modified Ashworth Scale and the Nociception Coma Scale-Revised (NCS-R).^133,135,140^ Besides common decreases in spasticity,^131,134,135,137-139^ significant behavioural improvements were reported, such as motor responses, decreased disability in daily activities, command following, communication and object manipulation.^131,133,137,138,140^ However, in a retrospective study with 26 patients with mixed aetiologies, decreased spasticity and increased CRS-R scores were not significantly correlated.^134^ Aetiology-related variabilities were also reported,^132,133^ with more responsiveness in TBI compared to hypoxic brain injury.^133^

Regarding the adverse effects, baclofen pump malfunction can result in either over/underdosing, which can induce mild to severe symptoms, such as weakness, tiredness, muscular hypotonia, hypothermia, and return of baseline spasticity.^136^

#### Midazolam

Midazolam is classified as a short-acting benzodiazepine, which activates GABA-A receptors and is commonly used to induce sedation and anaesthesia.^60,141^ Off-label use of midazolam (5 mg in 100 cc of saline solution within eight minutes) led to temporary emergence from MCS in an adult TBI patient. Parallel EEG assessment showed a more balanced distribution of low- and high-frequency cortical activity after midazolam. Interestingly, other medications such as amantadine, levodopa, and zolpidem had failed to improve consciousness in this patient.^142^ No adverse reactions were reported in this case report.

### Summary of the level of evidence on GABAergic agents

The LoE for GABAergic agents in DoC is heterogeneous and largely driven by zolpidem. Zolpidem has moderate evidence with several RCTs, single-patient controlled designs, observational studies, and case reports. Although its effects are not consistently demonstrated at the group level,^17,29^ ∼5–20% of patients have shown rapid but transient behavioural responsiveness.^31-33,101-106,109-112,119-121^ Neurophysiological and neuroimaging findings, including shifts from slower to faster EEG frequencies and increased cerebral blood flow and metabolism,^30-32,34,100,103,104,110,111,119-121,124^ provide converging support for a neurobiologically plausible responder endotype. The majority of RCTs (4/7) present a low RoB (see Risk-of-Bias Assessment section). Overall, confidence in zolpidem as a therapeutic option for DoC is moderate since the effect appears reproducible in selected responders, but the responder profile remains unknown. The literature, therefore, supports the experimental use and warrants methodologically robust mechanistic investigations. However, given that no or only minor adverse effects have been reported in the literature,^32,102,105,106,114,127^ its favourable safety profile may support a single-dose trial in routine clinical care to identify patients who might benefit from zolpidem.

In contrast, the LoE for baclofen as an awareness-enhancing therapeutic option is very low. Intrathecal baclofen has shown improvements in spasticity, dysautonomia, hypertonia and behavioural responsiveness, mostly in observational studies and case reports.^131,133,137,138,140^ However, favourable behavioural outcomes are difficult to interpret independently from baclofen-induced antispastic effects. The evidence for midazolam is very low and purely anecdotal, with a single case report.^142^ Overall, confidence in baclofen and midazolam for the treatment of DoC remains very low, and their use for this purpose should be considered experimental; however, baclofen remains the standard of care for the management of severe spasticity in DoC patients.

### Other pharmacological agents

#### Tricyclic antidepressants

As serotonin and/or norepinephrine reuptake inhibitors, tricyclic antidepressants are primarily used for depression and panic disorder.^143^ Yet, they have led to improved consciousness in three adult TBI patients.^144^ Amitriptyline (50 mg/day for 7 months) and desipramine (50 mg/day for 5 months in one patient and 75 mg/day for several months in another patient) improved arousal, response to command, functional communication, and ambulation as assessed with GCS and Rancho Los Amigos Scale (RLAS). Discontinuation of the treatment led to clinical deterioration in two of the three patients.^144^ In terms of adverse reactions, tricyclic antidepressants can increase the risk of seizures in TBI.^144^

#### Anticonvulsants

Anticonvulsants are referred to as anti-seizure medications with symptomatic effects used in the treatment of epilepsy, bipolar disorder, and neuropathic pain.^145,146^ Four studies on the administration of anticonvulsants (one retrospective study on different anticonvulsants and three case reports/series on lamotrigine, a glutamatergic agent) in 72 adult and 1 paediatric DoC patients showed contradictory results. In the retrospective study on 103 UWS and MCS patients with mixed aetiologies, anticonvulsants had no positive effect on the level of cognitive functioning in 54 medicated patients compared to the other 49 patients not treated with anticonvulsants.^147^ In the case report, lamotrigine (25–200 mg/day) improved arousal, cognition and communication in a TBI patient as assessed by LOCFAS and RLAS while methylphenidate, amantadine, and venlafaxine had previously failed to improve consciousness.^147,148^ The other case series on 13 patients in neurorehabilitation (mixed aetiologies) reported that lamotrigine (150–400 mg/day) led to marked improvements in arousal and cognition, as assessed by the RLAS, in nine patients and more modest improvements in the other four, with 10/13 patients finally discharged to community settings.^149^ Quite recently, in a case series of three adults and one paediatric patient with TBI, lamotrigine (25–100 mg/day for at least 4 weeks) resulted in command following, verbalization, goal-directed movements and/or functional communication and object use in all the patients. Discontinuation of lamotrigine led to worsened frontal syndrome in one patient.^150^

Regarding the adverse effects, disinhibited behaviour with verbal aggression was reported only in one patient after the administration of lamotrigine, but it was attributed to natural recovery from a severe brain injury.^148^ The case series did not report any major adverse events.^149^

#### Simvastatin

Simvastatin, an oral inhibitor of 3-hydroxy-3-methylglutaryl-coenzyme A (HMG-CoA) reductase, belongs to statins commonly used to reduce blood cholesterol levels.^151^ Yet, it has been found effective in the treatment of some brain disorders such as Parkinson’s disease, Alzheimer’s disease and Huntington’s disease.^152^

In a double-blind placebo-controlled RCT, simvastatin (40 mg daily for 10 days) was investigated in 49 patients with severe TBI and a GCS <8,^35^ with eight patients in VS. The patients were evaluated at admission (T1), discharge (T2) and 1 month post-discharge (T3). The study showed statistical significance in favour of the simvastatin group in GCS scores at discharge and 1-month follow-up and in terms of proportion of conscious patients at follow-up. No significant adverse effects were reported.^35^

#### Combination of treatments

A combination of drugs was reported in three studies (one pilot alternate treatment baseline-controlled trial and two retrospective studies), including 38 patients with acute and prolonged DoC (one paediatric, mixed aetiologies).^60,63,64,66,114^

In an alternate treatment trial of amantadine, repetitive transcranial magnetic stimulation (rTMS), and combined amantadine and rTMS in four patients with prolonged TBI-induced DoC, when rTMS was administered before amantadine, it doubled auditory-language gains, as reflected in EEG resting-state functional connectivity for language, salience and sensorimotor networks.^60^ In a retrospective study of 87 acute patients with non-TBI aetiology, three patients received both amantadine and modafinil, of whom one was considered a responder.^63^ In another retrospective case–control study, 84 patients in VS or MCS received either amantadine alone or in combination with cerebrolysin.^64^ Although improvements were reported in both groups, the dual regimen group showed greater change in the mean total CRS-R score and higher scores in visual and oromotor/verbal functions. The patients who benefited the most from the combination of drugs were in prolonged VS.^64^

### Summary of the level of evidence on other pharmacological agents

The LoE for other pharmacological agents is low, as it is largely based on small retrospective studies and case series. Although a single RCT has reported positive behavioural outcomes with simvastatin in TBI patients,^35^ the evidence represented a high RoB and requires further investigation. Improvements in consciousness with tricyclic antidepressants remain anecdotal with case series.^144^ Although no favourable outcomes were reported with anticonvulsants in the recovery of consciousness in a large retrospective study,^147^ lamotrigine showed potential benefits in a few case series^148-150^; however, the evidence remains inconsistent. Combined treatments also resulted in preliminary positive results with different combinations (i.e. amantadine coupled with rTMS, modafinil, or cerebrolysin).^60,63,64,66,114^ Overall, confidence in the clinical benefit of these combined interventions with at least one pharmacological component is very low, and the current evidence remains largely hypothesis-generating, limited by heterogeneous results, small samples and methodological inconsistencies. Consequently, these combinations for the treatment of DoC should be considered experimental, with no current evidence-based rationale for routine clinical application.

## Risk-of-bias assessment results

Among 22 RCTs (14 parallel and 8 crossover; Fig. 2A), six (27%) presented a high RoB,^20,22,25,26,29,35^ six (27%) raised some concerns^14,15,17,19,24,34^ and 10 (45%) had a low RoB (Fig. 2B). Approximately a quarter (27%) of RCTs were rated as having some concerns, while another quarter (27%) had a high RoB across most domains, although some domains, particularly deviation from intended interventions (D2; e.g. deviations from the protocol), missing outcome data (D3; e.g. exclusion of participants with missing data) and selection of reported results (D5; e.g. not handling missing data, reporting only significant findings), showed a higher frequency of high-risk judgment (Fig. 2A). Among crossover RCTs, one of them (12.5%) showed some concerns in carryover and period effects (DS; e.g. no/insufficient washout period).

**Figure 2:**
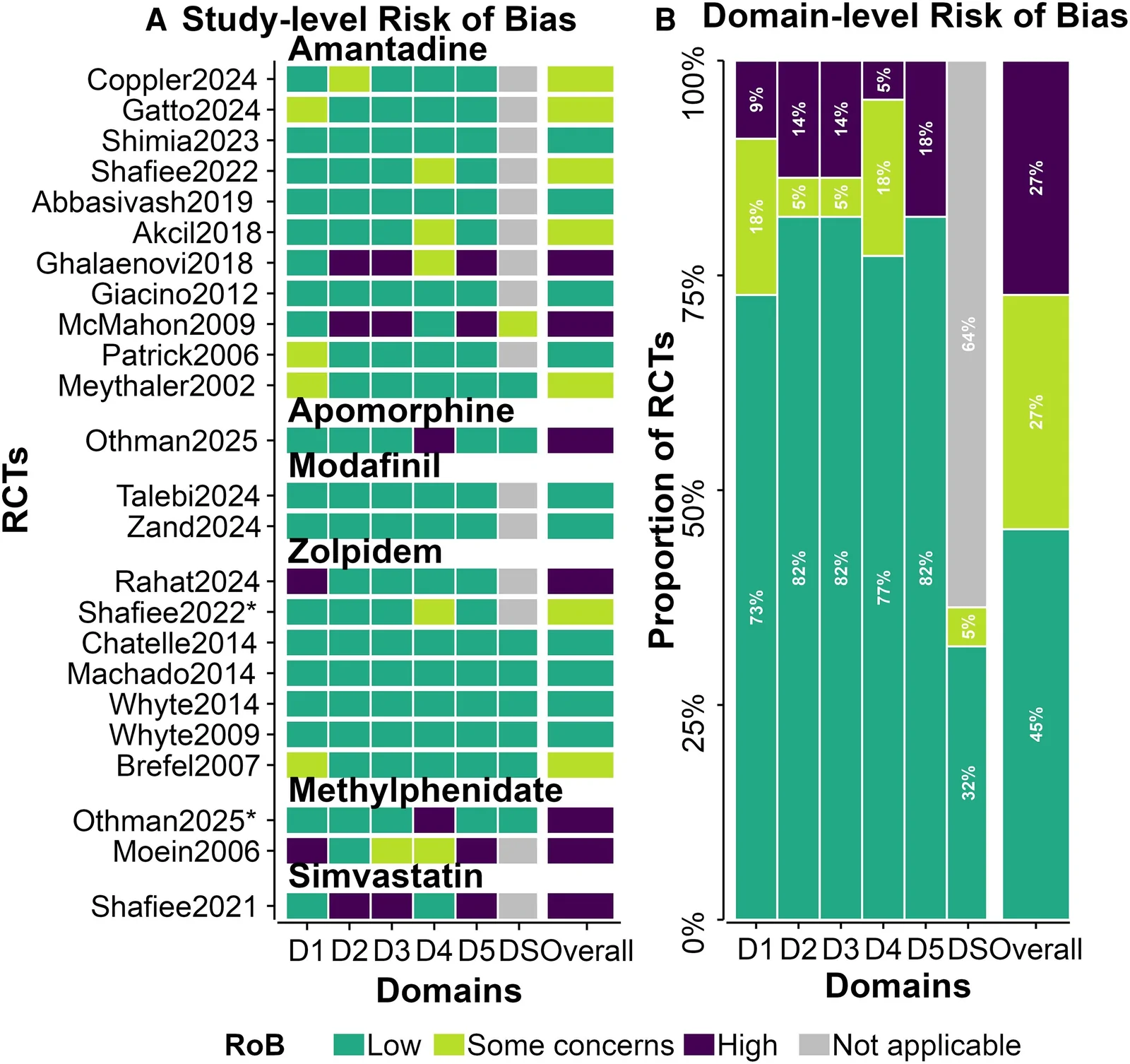
**(A) Study-level risk of bias (RoB) in randomized controlled trials (RCTs).** D1: bias arising from the randomization process; D2: bias due to deviations from intended interventions; D3: bias due to missing outcome data; D4: bias in measurement of the outcome; D5: bias in selection of the reported result; DS: bias due to carryover or period effects. (B) Domain-level RoB in RCTs. Stacked bar charts show the proportion of RCTs classified as low RoB, some concerns and high RoB within each RoB domain. The stacked bar on the right represents the overall RoB and the proportion of RCTs per RoB judgment. RCTs appearing with an asterisk (*) are shown twice since two pharmacological treatments are studied in these studies.

## Multiple correspondence analysis in RCTs

MCA was performed to visualize the proportion of corrected inertia (variance) explained by each dimension and RCT characteristics within the first two dimensions, contributing the most to the variance across RCTs on pharmacological treatments in DoC.

The first two dimensions of the MCA explained 18.9% and 14.7% of the total corrected inertia, respectively, accounting for ∼34% of the overall variability in study characteristics. Although the proportion of explained variance was distributed across 23 dimensions, the first two dimensions captured the most interpretable structure and were therefore further investigated below (Fig. 3A; see Supplementary Table 5).

**Figure 3:**
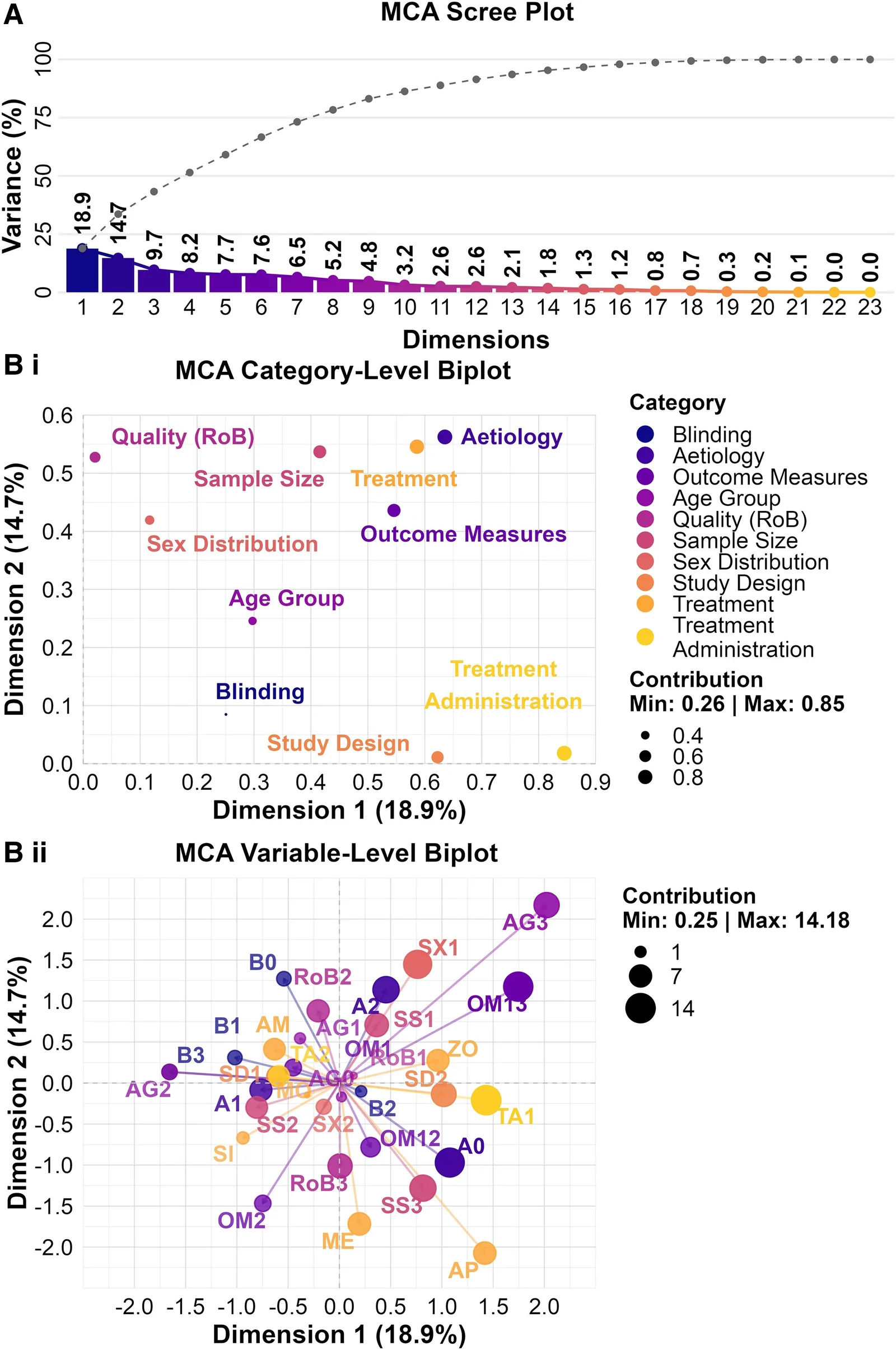
**(A) Scree plot of variance by dimension in the multiple correspondence analysis (MCA).** Each bar represents the percentage of variance (corrected inertia) explained by each dimension. The dashed line indicates the cumulative percentage of variance across dimensions. Colours follow a colour-blind-friendly sequential palette to enhance accessibility. **(B) (i) MCA category-level biplot.** Each point corresponds to the coordinates of a category of methodological characteristics on the first two MCA dimensions. Categories located farther from the origin are generally better represented by higher cosine squared (cos^2^) values and contribute more strongly to the MCA structure, whereas categories near the origin have lower discriminatory power. Point sizes indicate each category’s contribution to the two dimensions. **(B) (ii) MCA variable-level biplot.** Each point represents coordinates of a randomized controlled trial (RCT; *n* = 22) characteristic on Dimension 1 and Dimension 2. Variables that are closer together tend to co-occur in the same RCTs, whereas those located on opposite sides of an axis are negatively associated. Variables located far from the origin contribute most strongly to the corresponding dimension, while those near the origin are more frequent and less discriminating across RCTs. Point sizes indicate each variable’s contribution to the two dimensions. Point colours correspond to categories. Colours follow a colour-blind-friendly palette to enhance accessibility. Min: minimum; Max: maximum; B(0/1/2/3): blinding (none/single-blind/double-blind/triple-blind); A(0/1/2): aetiology (mixed, TBI and non-TBI); OM (1/2/12/13): outcome measure (neurobehavioural/clinical/neurobehavioural and clinical/neurobehavioural and neuroimaging); AG(0/1/2/3): age group (mixed/child-adolescent/young adult/middle-aged); RoB(1/2/3): risk of bias (low/some concerns/high); SS(1/2/3): sample size (small/medium/large); SX(1/2): sex distribution (female-dominant/male-dominant); SD(1/2): study design (parallel/crossover); TA(1/2): treatment administration (single/multiple administration); AM: amantadine; AP: apomorphine; MO: modafinil; ZO: zolpidem; ME: methylphenidate; SI: simvastatin.

In general terms, treatment administration and study design contribute the most to Dimension 1, while sex distribution and RoB contribute the most to Dimension 2. Pharmacological treatments, aetiology, outcome measures, sample size, and age group contribute to both dimensions and have more discriminatory power across RCTs from a methodological perspective (Fig. 3B(i); see Supplementary Table 6).

On a variable-level analysis, the first dimension (Dimension 1) primarily captures a methodological and interventional contrast. On the right-hand side, the plot is dominated by single administration, mixed aetiology, crossover design, neurobehavioural and imaging outcome measures, zolpidem and apomorphine. In contrast, the negative side of the first dimension is characterized by multiple administration, TBI aetiology, parallel design, and amantadine, simvastatin and modafinil. These variables are closer to young-adult, medium-size samples and to single- and triple-blind designs, reflecting more conventional therapeutic trial structures with repeated dosing. The second dimension (Dimension 2) mainly reflects differences in study quality and patients’ characteristics. The upper part of the plot is defined by some RoB, small sample sizes, female-dominant patient samples, no blinding and neurobehavioural outcome measures, indicating studies that are more clinically oriented but tend to exhibit less rigorous methodological quality. Conversely, the lower part of dimension 2 is associated with a high RoB, large sample size, male-dominant patient sample, neurobehavioural and clinical outcomes, and methylphenidate. Importantly, this dimension mostly reflects the relationship between methodological quality and sample size across RCTs (Fig. 3B(ii); see Supplementary Tables 7 and 8).

Taken together, the two dimensions reveal several coherent configurations. A clear cluster on the right side of the plot corresponds to zolpidem and apomorphine studies, mainly characterized by single administration, crossover design and combined neurobehavioural and imaging outcome measures, consistent with an exploratory and mechanistic research orientation. On the left side, amantadine, modafinil and simvastatin cluster with parallel designs and multiple administrations, reflecting more established therapeutic approaches. Methylphenidate occupies a more isolated position in the lower region, closely aligned with clinical outcomes and high RoB, highlighting its distinct methodological profile within the literature, with only two RCTs (one parallel and one crossover). Variables near the origin (e.g. double-blind design, mixed-age patient samples, and TBI aetiology) were common across RCTs and therefore contribute less to the differentiation of RCT profiles (see Supplementary Table 9). This structure might suggest that the findings may partly depend on methodological decisions (e.g. study design, treatment administration, outcome measure selection), rather than treatment effects alone.

## Limitations of current literature

As reviewed above, available pharmacological treatment options are foremost based on studies with small cohorts or uncontrolled cases, which cannot differentiate drug effects from spontaneous recovery. RCTs have been conducted as well, but their outcomes remain inconsistent, and 12 of 22 RCTs (∼60%) were judged to have some or high RoB.

Methodological heterogeneity across studies hinders meaningful comparisons and limit the generalizability of findings. These include notably differences in exclusion/inclusion criteria, treatment duration, dosing regimens, different or no follow-up assessments and various bedside behavioural assessments. The large heterogeneity of study samples or absence of information on sample characteristics, including demographics, clinical diagnosis and behavioural profile, brain injury mechanism and lesion sites, time since injury, concurrent treatments and medical comorbidities, accounts for the diverging results. In line with this, use of heterogeneous terminology (e.g. in terms of study design) and inconsistent or missing information (e.g. time since injury, limitations) introduce further heterogeneity of studies, hindering comparative studies and limiting replicability. Moreover, challenges such as limited patient availability, high costs associated with clinical trials, and difficulties in recruitment significantly impede reproducibility and generalisability of the findings.

While the current research focuses on prolonged DoC, there remains a paucity of evidence supporting pharmacological interventions in the acute phase of brain injury.^153^ Despite the complexities posed by ICU experiments due to medical instability, early interventions hold potential for augmenting recovery trajectories. The choice of intervention must be context-dependent, tailored to the setting and patient characteristics. Considering the limited understanding of the underlying pathophysiology of DoC and recovery mechanisms, it is important to note that differences in pharmacological mechanisms as well as the heterogeneity in brain lesion mechanisms and volume suggest that no single pharmacological agent is yet likely to benefit all DoC patients.

## Future perspectives on pharmacological treatments for disorders of consciousness

### Potential new pharmacological treatments

While several ongoing clinical trials are evaluating the effectiveness of existing drugs, parallel efforts are focusing on the potential of new theory-driven interventions. A growing consensus suggests that consciousness is closely linked to brain complexity: when individuals are consciously experiencing something, their brain activity exhibits higher complexity than when no experience is present.^154^ Building on this idea, a recent proposal has suggested leveraging this property as a novel approach to enhance consciousness in patients with DoC. Psychedelics, including classic 5-HT2A agonists such as psilocybin and atypical agents such as the NMDA antagonist ketamine, have been shown to increase brain complexity in healthy individuals.^155-157^ When given to patients with DoC, psychedelics might thus boost their brain complexity and, in turn, expand their behavioural repertoire or richness of phenomenological experiences.^158,159^ At present, the published clinical evidence is limited to a feasibility study administering an intravenous sub-anaesthetic dose of ketamine to 3 patients with prolonged DoC, and a single case report describing psilocybin administration in a patient in MCS.^160,161^ The results demonstrated no positive effects on overt behaviours as assessed with the SECONDs, but an overall increase in brain complexity.^160,161^ In addition, a large retrospective analysis informed by deep-learning drug screening identified saxagliptin (a dipeptidyl peptidase-4 inhibitor) as a potential therapeutic agent in acute settings.^162^

### Modelling and simulation of pharmacological treatments

Whole-brain models are useful preclinical and theoretical tools that can be used to explore pharmacological treatments (Fig. 4). In essence, they are generative models that replicate physiological data, such as EEG or fMRI BOLD signals of a particular target state. The target state could take several forms, from a particular condition or disorder at the group level to individual patients. Whole-brain models are ‘semi-empirical’ in the sense that empirical multimodal imaging data are used to optimize the model. At their base, the models use a mathematical representation of the local neural activity. This could take several forms, one commonly employed model is based on Stuart–Landau oscillators, which vary between two dynamical regimes. The dynamics at the Hopf bifurcation between these two regimes have been shown to have similar dynamical properties to BOLD signals. Other more biologically realistic models (e.g. dynamic mean-field models) model mean-field reductions of integrate and fire spiking neurons with excitatory and inhibitory populations. Whole-brain modelling has been previously employed to investigate the dynamical properties and mechanisms defining several states of consciousness, including pharmacologically induced states,^163-165^ non-pharmacological states and pathological DoC.^166-170^

**Figure 4:**
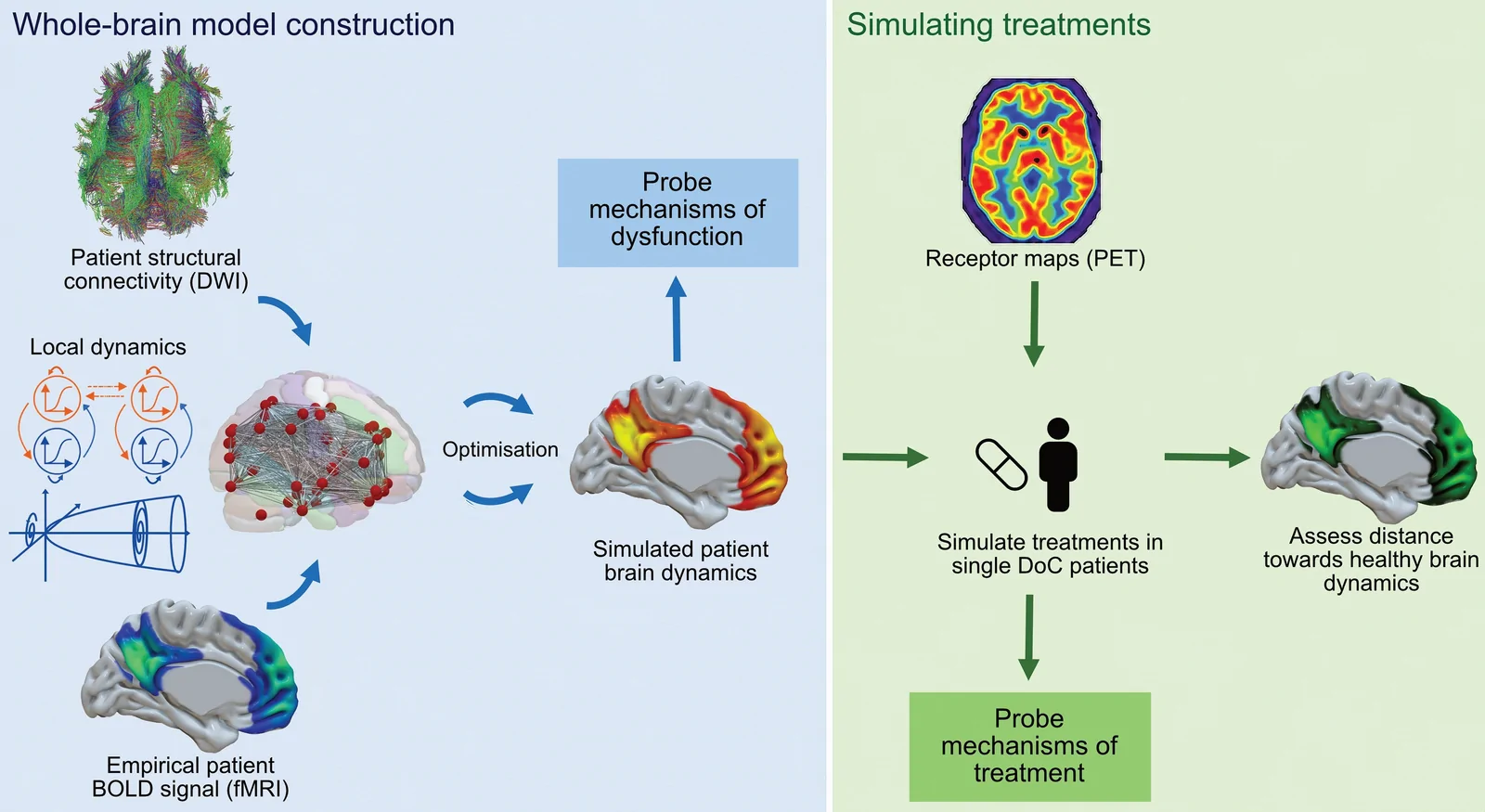
**Schematic showing the construction of computational whole-brain models and their potential uses in drug discovery and personalized medicine.** Left, the brain is parcellated into several nodes, which are subsequently connected by the empirical structural connectivity from diffusion-weighted imaging (DWI). The empirical resting-state functional magnetic resonance imaging (fMRI) dynamics are used to optimize the model. Local dynamics can take several forms, including supercritical Hopf bifurcations (bottom) or the dynamic mean field (top). Extracted parameters or other metrics from models at the group, or single-subject level, can be used to better understand the mechanisms underlying disorders of consciousness (DoC). Neurotransmitter information from positron emission tomography (PET) can also be added to the model, which can facilitate the simulation of pharmacological perturbations. In the future, single-subject models could be constructed on which different treatments could be simulated. Adapted from Alnagger *et al*. (2023)^172^ under the terms of the Creative Commons Attribution License (CC BY).

Whole-brain models have also been used in several productive ways for pharmacological treatments for patients with DoC. Having the model parameters entirely accessible allows researchers to perform simulations that would be experimentally challenging or even impossible. This is directly relevant to psychedelic drugs. Due to the current legal status of psychedelics, conducting RCTs presents significant challenges. This makes every step, from obtaining ethical approval to securing the necessary support and expertise required to design and perform such studies, particularly difficult. Recent research has leveraged whole-brain modelling to provide the first steps in investigating these treatments. A recent study used individualized whole-brain models, built upon the patient structural data given by the diffusion-weighted imaging, and functional data given by the resting-state BOLD signal.^170^ These digital twins of each patient were subjects in a virtual clinical trial in which the administration of a psychedelic drug was simulated and the resulting change in brain dynamics were assessed. Psilocybin and lysergic acid diethylamide (LSD) were shown to increase the complexity of the modelled patient brain activity, with a greater effect in MCS patients compared to UWS patients. Here, the modelled responses to artificial perturbations were denoted as proxies for the complexity of brain activity. Interestingly, the treatment effect in patients with UWS was associated with the average patient structural connectivity, whereas in patients in MCS, the treatment effect was associated with the average patient functional connectivity. This implies that increases in complexity need sufficient underlying structure to manifest. More broadly, another recent study used biophysical whole-brain models optimized to patients with DoC at the group diagnostic level in conjunction with PET-derived receptor maps to simulate pharmacological receptor stimulations.^167^ Upon assessing the simulated stimulation of multiple receptors, the resulting trajectory of patient dynamics was quantified in space using variational autoencoders and compared to the spatial representation of healthy controls. In support of the potential of psychedelic treatment, the 5-HT2A and 5-HT1A receptor stimulations were shown to bring the brain dynamics of DoC patients closest to the dynamics of healthy controls.

While the research is still in its infancy, these studies show the potential of whole-brain modelling to investigate theoretical pharmacological efficacy, identify potential candidates with promising treatment responses and investigate the underlying treatment mechanisms.^50^ In time, modelling-based predictions could be validated with real-world observations, continually refining the models to improve their accuracy. As the accuracy of the models continues to improve, driven by improving technology and methodology, whole-brain models are poised to make a meaningful impact in drug discovery and personalized medicine.^171-173^ Nevertheless, routine clinical applications of these models remain some time away.

### Potential mechanisms of action

Each of the pharmacological agents reported in this review modulates specific neuronal pathways. Based on pharmacodynamic and pharmacokinetic properties, pharmacological agents modulate neuronal pathways involved in consciousness through specific receptors and neurotransmitters to attenuate lesion-induced perturbations and thus promote consciousness recovery.^50,174^ Although the neurological mechanisms underlying the therapeutic effects of these pharmacological agents are not yet fully elucidated, some evidence is emerging in the literature.

The anterior forebrain mesocircuit model provides a conceptual framework for representing recovery mechanisms of DoC as well as for investigating the effects of neuropharmacological interventions (Fig. 5).^175^ This model suggests a significant deafferentation of loop connections between the frontal cortex, striatum, pallidum, and central thalamus due to a downregulation of background synaptic activity induced by severe brain injuries.^175,176^ Striatal neurons inhibit the internal globus pallidus, relying on robust basal synaptic activity and elevated dopaminergic innervation for maintenance. Disruption of striatal projections to the globus pallidus, due to insufficient dopaminergic innervation, results in globus pallidus-mediated inhibition of central thalamic nuclei, subsequently leading to cortical inhibition and potentially causing DoC.^175^ These ascending and descending pathways collectively modulate the state of consciousness.^175^

**Figure 5:**
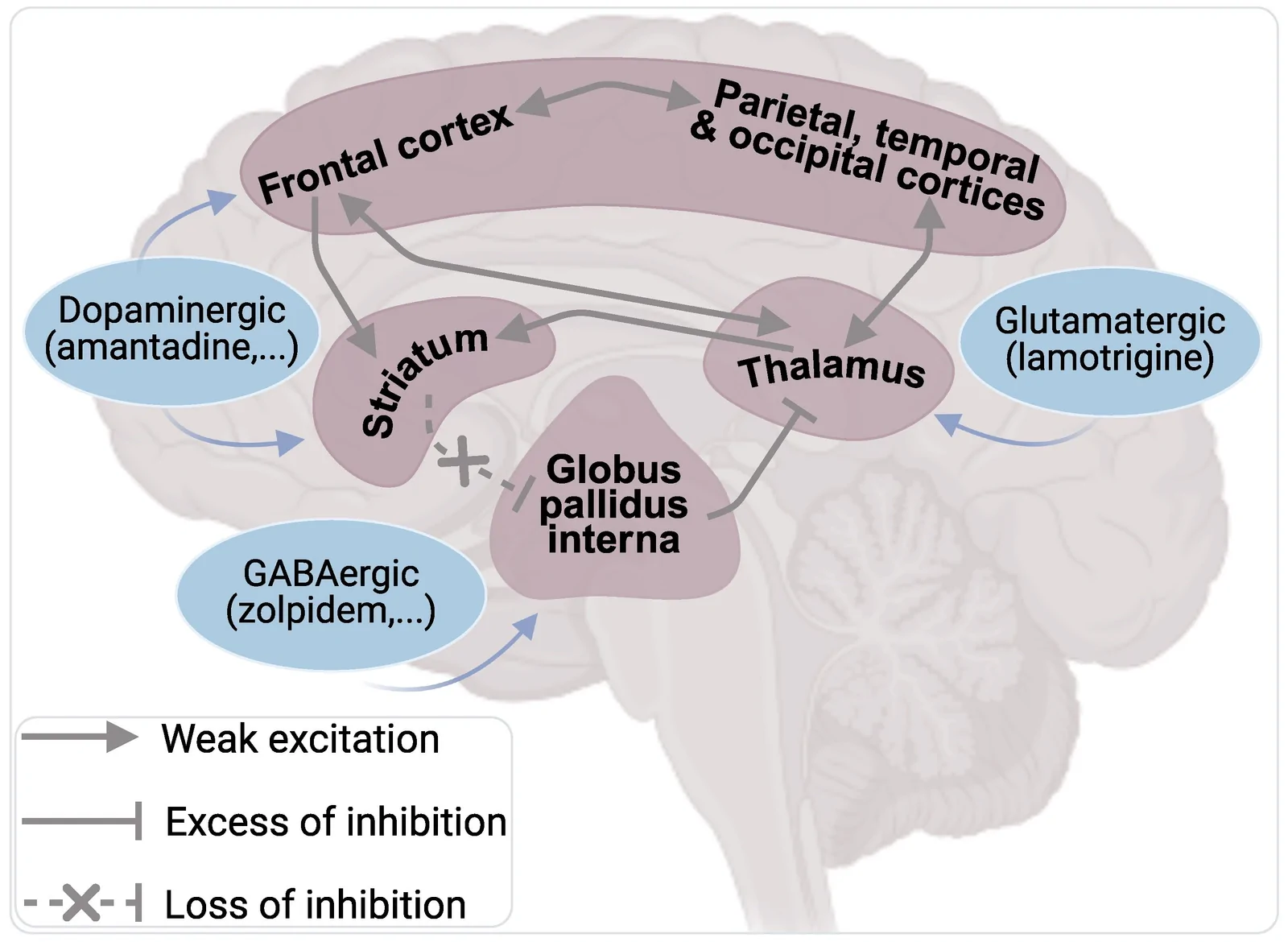
**Representation of dopaminergic and GABAergic pharmacological effects based on the fronto-parietal mesocircuit model.** In physiological conditions, the striatum inhibits the pallidum, facilitating thalamic activation of cortical networks, thereby sustaining the cortico-subcortical loop. In pathological conditions, neuronal deafferentation results in a loss of thalamostriatal and corticostriatal projections, leading to reduced striatum activity, inhibition of thalamic activity, and reduced cortical activation. Dopamine agents are thought to act primarily on striatal dopamine neurons and GABAergic agents on globus pallidus interna GABA neurons. These pharmacological agents would thus help restore striato-pallido-thalamo-cortical loops. Adapted from Szymkowicz *et al*. (2024)^174^ with permission. Created in BioRender. Seyfzadeh, F. (2026) https://BioRender.com/py1hapj.

Dopaminergic agents (e.g. amantadine) are thought to enhance striatal projections to the globus pallidus, modulating frontoparietal cortical neurons and re-establishing cortico-subcortical loops.^177^ GABAergic agents would act at different sites; notably, zolpidem would produce broad direct excitation across the frontal cortices and striatum as well as inhibition of the globus pallidus.^177^ Tricyclic antidepressants act by blocking the reuptake of norepinephrine and serotonin neurotransmitters at presynaptic terminals, thereby increasing noradrenergic and serotonergic neurotransmission.^144,178^ Anticonvulsants act on ion channels (typically by blocking sodium channels) or by enhancing inhibitory GABAergic transmission, thereby affecting neuronal excitability properties.^179^ Neuroprotective and neurorestorative agents reinforce endogenous defensive mechanisms activated following brain injury: simvastatin exerts pleiotropic properties for preventing platelet aggregation, inflammation and endothelial dysfunction; and cerebrolysin induces neurotrophic stimulation, modulates neuroprotection against toxic agents, and enhances neuronal metabolism by increasing resistance to hypoxic conditions and preventing lactate accumulation.^152,180^

Translational, multi-scale and multimodal data should be combined with new biomarkers, theory-driven approaches and computational models to improve the mechanistic understanding of DoC.^50^ To ensure the generalization of results, data need to be collected across different states of altered consciousness (e.g. sleep, anaesthesia, seizures) and from the same patients over time.^50^ A bidirectional interaction between scientific research and clinical practice is essential to ensure that scientific advances support clinical practice and that clinical insights guide future research.^50,180^ Future research should focus on (i) differentiating the neural mechanisms of consciousness from those underpinning connectedness to the environment and behavioural responsiveness, (ii) integrating structural brain damage and abnormal brain function in DoC, (iii) bridging micro- and macro-scale neural phenomena and (iv) exploring possible synergies between data-driven and theory-driven approaches.

### Development of a registry

While several studies have reported potential benefits of pharmacological treatments in patients with DoC, it is equally important to acknowledge negative or null results, as they highlight the complexity of pharmacological modulation of consciousness and the strong influence of methodological variability and patient heterogeneity on outcomes. Therefore, to determine the validity of pharmacological treatments in DoC, a community effort with multicentric experiments, standardized data collection, and transparent reporting is necessary. This will facilitate replications and provide more precise and advanced mechanistic insights into the therapeutic efficacy of pharmacological treatments in DoC and personalized care.

Legal and institutional regulations regarding consent can also limit patient participation, leading to small-sample underpowered studies, particularly in pharmacological trials; therefore, the development of an international registry and large-scale data sharing initiatives may help mitigate this barrier. The Curing Coma Campaign has already taken steps in this direction by developing standardized common data elements for DoC.^181,182^ Notably, Monti and colleagues (2024)^183^ proposed a common dataset on therapeutic interventions for patients with DoC. This minimal dataset should include patient information (diagnosis of DoC, comorbidities, medications and doses, time of initiation of treatment), general study information, behavioural interventions, pharmacological and/or medical device interventions. Adoption of these collective data sharing and management practices will reduce publication bias, facilitate a more balanced and exhaustive evaluation of different pharmacological treatments studied in DoC, and work towards establishing the most effective treatments in DoC.

In addition to methodological and clinical constraints, practical real-world factors also limit the translation of favourable therapeutic outcomes in DoC into clinical practice. Drug availability, cost, and operational differences across healthcare systems influence treatment accessibility and implementation. These factors collectively constrain real-world applicability through logistical, regulatory, and economic challenges. More methodologically rigorous and operationally feasible research with systematic collection of safety and cost-effectiveness data can help address these barriers. Therefore, the development of a registry would also enable more accurate evaluation of treatment efficacy across diverse healthcare contexts and facilitate the development of contextualized clinical guidelines.

## Conclusion

Current evidence on consciousness-enhancing pharmacological treatments in DoC remains limited. The literature is dominated by retrospective studies, small cohorts, and case reports, with only a few RCTs available, characterized by substantial heterogeneity. The available literature on pharmacological treatments in DoC is thus not conclusive on the most effective treatments. Although several pharmacological agents have shown potential, their efficacy remains uncertain and may reflect an interaction between treatment-induced favourable outcomes and spontaneous recovery. From a therapeutic perspective, as the current literature focuses on efficacy rather than effectiveness in pharmacological trials, translation of the findings into clinical practice remains far-fetched. Consequently, methodologically rigorous, multicentre studies and systematic investigation of new and theory-driven pharmacological treatments with further investigations into mechanisms of action are required to push scientific and clinical boundaries. Equally critical is the standardization of data collection and reporting practices, as inconsistent outcome measures and missing information currently jeopardize reliable evaluation and comparison across studies. To address these limitations, the development of an international registry for pharmacological interventions in DoC is essential. Such an initiative would enable more robust comparative studies, reduce publication and reporting biases, facilitate replicability, robustness and generalizability, and ultimately support the identification of the most effective patient-tailored treatments for DoC.

## Supplementary Material

fcag369_Supplementary_Data

## Data Availability

Data sharing does not apply to this article as no new patient-level data were generated or analysed in this study. The R script for MCA and related visualizations is available online at https://github.com/FatemehSeyfzadeh/MCAonRCTs.
