# Supplementary material for: Pharmacological treatments in disorders of consciousness
Source: Brain Commun. 2026 Oct 6;8(6):fcag369. doi: 10.1093/braincomms/fcag369 (PMC13765538; doi:10.1093/braincomms/fcag369)
Supplement: Supplementary file 1 [file fcag369_supplementary_data.pdf]

Pharmacological treatments in disorders of consciousness

Christian Saleh[1*#], Fatemeh Seyfzadeh[2,3,4*], Emilie Szymkowicz[2,3*], Naji Alnagger[2,3], Tatiani Soultana Ilia[5], Alexandra A. Oancea[6], Olivia Gosseries[2,3#]

Supplementary Background

Behavioural diagnostic tools in DoC

A summary of the currently available diagnostic tools to behaviourally assess treatment effects in DoC patients is presented in **Supplementary Box 1**.

Supplementary Box 1. Behavioural diagnostic tools to assess treatment effects

Precise diagnosis is essential for guiding treatment and for interpreting the effects of therapeutic interventions. Differentiating between UWS and MCS remains challenging, with error rates approaching 40% when standardised behavioural assessments are not used. [1-3] The first widely adopted bedside tool was the **Glasgow Coma Scale (GCS)**, which is based on eye opening (maximum score of 4), verbal response (maximum score of 5), and motor response (maximum score of 6), for a total score of 15. [4] However, the GCS does not reliably distinguish UWS from MCS. A major advance came with the **Coma Recovery Scale-R (CRS)**, which comprises six hierarchical subscales (auditory, visual, motor, oromotor/verbal, communication, and arousal), ranging from reflexive behaviours to cognitively mediated responses. [5,6] Diagnosis is based on the presence of specific behaviours (e.g., visual pursuit indicates MCS). Although scores ≥10 are often associated with MCS, diagnosis should rely on behavioural items rather than total score, as some MCS patients score below this threshold. [6] Several complementary rapid assessments have also been recently developed. The **Simplified Evaluation of Consciousness Disorders (SECONDs)** scale provides a simplified tool derived from the most frequent MCS behaviours and facilitates quick bedside evaluation. [7] The **CRS-R For Accelerated Standardised Testing (CRSR-FAST)** adapted the CRS-R for use in intensive care units. [8] Functional outcomes in TBI rehabilitation is often measured with the **Disability Rating Scale (DRS)**, which evaluates impairment, disability, and handicap. [9] Other instruments used in DoC research include the **Coma/Near Coma Scale (CNCS)**, **Glasgow Outcome Scale (GOS; GOS-E)**, **Western Neuro Sensory Stimulation Profile (WNSSP)**, **Wessex Head Injury Matrix (WHIM)**, **Levels of Cognitive Functioning Assessment Scale (LOCFAS)**, **Rancho Los Amigos Scale (RLAS)**, **MATADOC**, **Mini-Mental Status Examination (MMSE)**, **modified Rankin Scale (mRS)**, **Cerebral Performance Categories Scale (CPC)**, **Sensory Tool to Assess Responsiveness (STAR)**, **Sensory Modality Assessment and Rehabilitation Technique (SMART)**, **Functional Independence Measure (FIM), and Karnofsky Performance Scale (KPS)**. [10-24] These scales have been mostly applied across studies evaluating pharmacological interventions, typically in pre–post treatment designs.

Supplementary Methods

Search query

A non-systematic literature search was conducted on PubMed and Scopus databases to identify studies on pharmacological treatments in disorders of consciousness (DoC) published before December 2025. The search strategy combined controlled vocabulary (e.g., MeSH terms on PubMed) and free-text terms, and was conducted in two stages to ensure broad identification of eligible studies and comprehensive coverage of identified pharmacological treatments in DoC. An initial search was performed to identify studies investigating pharmacological treatments in DoC (**Query 1**). Based on the agents identified in the initial search, additional targeted searches were performed (**Query 2**).

Query 1. Initial search query

```
(pharmacologic OR pharmacotherapy) AND ("disorders of consciousness" OR "disorder of consciousness" OR "unresponsive wakefulness syndrome" OR "vegetative state" OR "minimally conscious state")
```

Query 2. Follow-up search query

```
(amantadine OR levodopa OR apomorphine OR methylphenidate OR bromocriptine OR modafinil OR selegiline OR zolpidem OR baclofen OR midazolam OR anticonvulsants OR antiepileptics OR “tricyclic antidepressants” OR simvastatin) AND ("disorders of consciousness" OR "disorder of consciousness" OR "unresponsive wakefulness syndrome" OR "vegetative state" OR "minimally conscious state")
```

Multiple Correspondence Analysis (MCA)

MCA operates on a complete disjunctive table, where each category of every variable is represented by a binary indicator. Distances between studies were computed using the chi-squared metric, giving greater weight to rare categories, and the centred, weighted data matrix was decomposed by singular value decomposition to extract factorial axes ordered by decreasing inertia (variance explained). The analysis was conducted in R (Version 4.4.0) using the FactoMineR package and visualised with factoextra. Eigenvalues (corrected inertia), category coordinates, contributions, and squared cosine (cos^2) values were examined to interpret the dimensions. To ensure transparency and reproducibility, Supplementary Tables 5-9 provide detailed numerical outputs of the MCA in terms of coordinates of each category on MCA dimensions, category-level and variable-level contributions to the construction of dimensions, and the quality of representation (cos^2) of each category on each dimension. Together, these outputs allow a detailed assessment of which RCT characteristics most strongly structure the MCA space, how well categories are represented on the retained dimensions, and how methodological, clinical, and demographic characteristics contribute to heterogeneity across pharmacological RCTs in DoC.

Supplementary Tables

Supplementary Table 1. RCTs included in the review

Authors (year)	Type of study<br><br>*LoE	Number of patients & Sex distribution	Age[1]	Aetiology	Time since injury[2]	Diagnosis/State of consciousness[3]	Outcome measure(s)	Dose & duration[4]	Outcome(s)[5]	Reported limitations
**DOPAMINERGIC AGENTS**										
**Amantadine**										
**Coppler et al. (2024)** [25]	Double-blind multicentre pilot RCT<br><br>*Level 1b	Total: 14 (10 male)<br><br>• Amantadine: 7 (6 male)<br>• Placebo: 7 (4 male)	• A adine: 63 (range: 48-65)<br>• P o: 55 (range: 52-62)	Cardiac arrest	72-120h after resuscitation	Comatose patients; DoC states not specified	Awakening (following 2-step commands) within 28d & time to awakening, Length of stay, FOUR (motor & brainstem components), Survival to hospital discharge	100mg BID, 7d	• No significant difference in awakening within 28 days between amantadine (28.6%) and placebo (42.9%) groups<br>• Non-significantly shorter median time to awakening with amantadine (4 days) *vs.* placebo (13 days)<br>• No significant differences in length of hospital stay or survival to hospital discharge between amantadine and placebo<br>• Death of 4 patients in amantadine and 3 patients in placebo group, with 6 deaths due WLST and 1 due to hemodynamic instability	• Premature termination of RCT before reaching the pre-specified sample size<br>• Limited statistical power<br>• RCT initiation 72-120h after return of spontaneous circulation<br>• High proportion of deaths due to WLST, introducing potential bias
**Gatto et al. (2024)** [26]	Double-blind RCT<br><br>*Level 1b	Total: 37 (13 male)<br><br>• Amantadine: 20 (5 male)<br>• Placebo: 17 (8 male)	59.7y± 11.4 (range: 28-81y<br>• A adine: 61± 9.1y<br>• P o: 57.8±13y	Aneurysmal SAH	Median: 53d, range: 30-123d	Severe DoC; GCS mean: 7.2±2.3	mRS<br>GOS<br>DRS<br>CRS-R<br>mortality	100-400mg/d, 6w	• No clinical or statistically significant superiority of amantadine over placebo<br>• No between-group differences in aneurysm treatment modality, surgical complications, vasospasm, delayed cerebral ischemia, central nervous system infections, overall mortality, adverse drug effects, or neurological recovery<br>• Higher need for emergency external ventricular drainage in the amantadine group, increased sepsis in the placebo group, and association between mortality and worse baseline neurological status	• Small sample size and limited statistical power<br>• Absence of treatment effects probably linked to delayed medication initiation<br>• Potential confounding effects of bias by indication and treatment timing<br>• Limitations of consciousness assessment with GCS in intubated or tracheostomised patients

										☙ Lack of routine continuous EEG monitoring to detect breakthrough seizures<br>☙ Insufficient data to inform optimal dosing or combination strategies
**Shimia et al. (2023)** [27]	Triple-blind RCT<br><br>*Level 1b	Total: 57 (45 male)<br><br>☙ Amantadine 29 (22 male)<br>☙ Placebo 28 (23 male)	☙ A adine: 29.8 (range: 21-34.5)<br>☙ P o: 28.9 (range: 20.2-37.5)	TBI	/	GCS≤8<br><br>☙ A adine: GCS scores 5.59±1.55<br>☙ P o: GCS scores 5.25±1.69	DRS<br>GOS	200-400mg/d, 43d	☙ Both groups showing significant improvement in DRS scores over the trial period<br>☙ Greater change from baseline in DRS scores in the amantadine group<br>☙ Fewer patients with (extremely) severe DRS scores with no patient in VS in the amantadine group, compared to one VS patient in the placebo group at week 6<br>☙ Improved GOS scores in both groups	☙ Small sample size<br>☙ Short time interval<br>☙ No washout period<br>☙ Insensitivity of GOS for measuring recovery rates in short-term periods
**Shafiee et al. (2022)** [28]	Single-blind RCT<br><br>*Level 2	Total: 66 (45 male)<br><br>☙ Amantadine 22 (15 male)<br>☙ Zolpidem 22 (15 male)<br>☙ Placebo 22 (15 male)	☙ A adine: 40.72±14.58<br>☙ Z em:40.22±14.65<br>☙ P o: 47.40±16.01	TBI	/	GCS≤8<br><br>☙ A adine: GCS scores 5.40 ±1.14<br>☙ Z em: GCS scores 5.50±1.30<br>☙ P o: GCS scores 5.77±1.14	GCS<br>GOS<br>Duration of mechanical ventilation<br>Length of hospitalisation	200mg/d, 8d	☙ Similar GCS scores across all 3 groups during the first 3 days with the scores increasing from day 4<br>☙ Largest GCS score increases in the amantadine group<br>☙ Highest mean GCS on the third day after discontinuation in the amantadine group<br>☙ Noticeable upward trend in both amantadine and zolpidem groups, compared to slow GCS score increase in the placebo group<br>☙ Greater changes in GCS scores from admission to day 11 in the amantadine group (107%) compared to zolpidem and placebo groups (58% and 31.5%, respectively)<br>☙ 2 mortalities in the amantadine, 2 in the placebo, and 1 in the zolpidem group<br>☙ No significant differences between groups in terms of mechanical	☙ No follow-up after discharge

									ventilation duration or length of hospitalisation	
**Abbasivash et al. (2019)** [29]	Double-blind RCT<br><br>*Level 1b	Total: 66 (46 male)<br><br>• Amantadine: 33 (22 male)<br>• Placebo: 33 (24 male)	• A adine: 39.63±13.08<br>• P o: 35.84±11.25	TBI	/	GCS≤8<br><br>• A adine: GCS scores 5.72±1.58<br>• P o: GCS scores 5.51±1.64	GCS<br>GOS<br>Duration of mechanical ventilation<br>Length of hospitalisation	200-400mg/d<br><br>Duration not specified ("continued until the desired outcome, death, or complication")	• No significant between-group differences in GCS at discharge<br>• No significant between-group differences in the duration of mechanical ventilation<br>• No significant between-group differences in length of hospitalisation<br>• 2 mortalities in the amantadine and 3 mortalities in the placebo group and discharge	• No follow-up after discharge<br>• Lack of clear consensus regarding radiological diagnosis of DAI<br>• Small sample size
**Akcil et al. (2018)** [30]	RCT<br><br>*Level 2	Total: 12 (7 male)<br><br>• Amantadine: 5 (3 male)<br>• Control: 7 (4 male)	• A adine: 50.6±11.86<br>• P o: 54.57±8.44	SAH	<2d	GCS≤8	CRS-R<br>DRS	200mg/d IV, 5d<br>200mg/d enteral, 25d	• Higher CRS-R scores on day 5, and at 3 and 6 months in both groups, compared to baseline<br>• Lower DRS scores on day 5, and at 3 and 6 months in both groups, compared to baseline<br>• Lower DRS scores on day 5, and at 3 and 6 months in the amantadine group, compared to placebo	• Small sample size
**Ghalaenovi et al. (2018)** [31]	Double-blind RCT<br><br>*Level 1b	Total: 40 (37 male)<br><br>• Amantadine: 19 (19 male)<br>• Placebo: 21 (18 male)	• A adine: 32.16±13.87<br>• P o: 40.95±20.86	TBI	• A adine: 3.21±2.32d<br>• P o: 3.42±2.67d	• A adine: GCS scores 7.1±1.56<br>• P o: GCS scores 6.95±1.74	GCS<br>FOUR<br>MMSE<br>GOS<br>DRS<br>KPS	200mg/d, 6w	• No significant between-group differences in the mean hospitalisation time<br>• Greater GCS score increases from day1 to day7 in the amantadine group, compared to placebo<br>• 6 mortalities in the amantadine group and 5 mortalities in the placebo group<br>• No other significant between-group differences	• No post-treatment assessments<br>• Exclusion of patients with drug-limitation factors
**Giacino**	Multicentre Double-	Total: 184 (133 male)	• A adine:	TBI	• A adine: 48d	VS<br>MCS	DRS<br>CRS-R	200-400mg/d, 4w	• DRS improvements in both groups over 4 weeks	• Patient selection bias<br>• Not generalizable to non-white patient populations

**et al. (2012)**[32]	blind RCT<br><br>*Level 1b	• Amantadine:87 (64 male)<br>• Placebo:97(69 male)	35.5±15.3<br>• Po: 37.2±15.4		(range: 38-66)<br>• Po: 47d (range: 37-65)				• Faster recovery in the amantadine group, compared to placebo<br>• Fewer dose increases required in the amantadine group at weeks 2 and 3<br>• Faster recovery in patients with smaller TSI and in MCS patients<br>• More patients in the amantadine group showing favourable DRS outcomes and fewer remaining in a VS after 4 weeks<br>• Greater percentage of amantadine patients recovering key CRS-R behavioural benchmarks (no statistical comparisons)<br>• Follow-up DRS improvements in the placebo group at weeks 5 & 6<br>• Slowing of recovery in the amantadine group at weeks 5 & 6<br>• Maintained behavioural improvements in the amantadine group despite decreased between-group differences by week 6	• Brief treatment interval and short-term outcome assessment<br>• No restriction of standard rehabilitation interventions<br>• Concomitant use of potentially confounding psychoactive drugs<br>• No monitoring of subclinical seizures
**McMahon et al. (2009)**[33]	Double-blind crossover RCT<br><br>*Level 1	Total: 7 paediatric (6 male)	12.7 (range: 6-18)	TBI	4-10w (mean: 6w)	UWS<br>MCS	CNCS CRS-R<br>Wee-FIM<br>Physician- and parent-rated arousal and consciousness<br>Nightly Sleep Scale	4mg/kg daily, 1w<br>6mg/kg daily, 2w	• No significant slope differences in CNCS, CRS-R, or Wee-FIM weekly scores<br>• Non-significant individual favourable slopes in some patients during amantadine<br>• Physicians reporting greater improvements in consciousness during amantadine (4/6) vs. placebo (1/6)<br>• No significant differences in physician-rated arousal between conditions<br>• No significant differences in parent-rated consciousness or arousal<br>• No significant differences in nightly Sleep Scale	• Small sample size<br>• Heterogeneity of patients

**Patrick et al. (2006)** [34]	Double-blind RCT<br><br>*Level 1b	Total: 10 (6 paediatric; 4 male)<br><br>- Amantadine: 6<br>- Pramipexole: 4	16.7 (range: 18-21)	TBI	32-232d	GCS: 3	CNCS<br>WNSSP<br>RLAS<br>DRS	- tadine: 50-200mg daily, 6w<br><br>- pexole: **<10y:** 0.003-0.021mg/kg/d **10-14y:** 0.125-0.75mg/kg/d **14-21y:** 0.25-1.5mg/kg/d	- Improved CNCS, WNSSP and DRS scores from baseline for both amantadine and pramipexole<br>- Minimal improvement premedication, maximal improvement during medication phase, and stable but non-progressive improvement after weaning<br>- Medication-phase gains exceeding post-medication gains for CNCS and DRS and exceeded premedication gains for WNSSP as the most sensitive outcome measure<br>- No significant efficacy difference between amantadine and pramipexole, with notable inter-individual variability; non-response occurring mainly in patients starting at RLAS level II, while patients at RLAS level III showing significantly better responses	- Small sample size
**Meythaler et al. (2002)** [35]	Double-blind crossover RCT<br><br>*Level 1	Total: 35 (26 male)<br><br>- Amantadine-first: 15<br>- Amantadine-second: 20	31 (range: 16-69)	TBI (DAI)	2-6w	GCS≤10<br><br>GCS mean score: 5.4±2.4	MMSE<br>DRS<br>GOS<br>FIM-cog<br>GOAT<br>ABS	200mg/d, 6w	- MMSE improvements during amantadine but not during placebo in the amantadine-first group<br>- MMSE improvements during placebo and amantadine in the placebo-first group<br>- Faster recovery during amantadine compared to placebo in both groups<br>- DRS improvements during amantadine but not during placebo in the amantadine-first group<br>- DRS improvements during placebo and amantadine in the placebo-first group<br>- GOS improvements during amantadine but not during placebo in the amantadine-first group	/

									☙ GOS improvements during placebo but not during amantadine in the placebo-first group<br>☙ FIM-cog improvements during amantadine but not during placebo in the amantadine-first group<br>☙ Significant FIM-cog improvements during placebo and amantadine in the placebo-first group<br>☙ No significant changes or trends in GOAT or ABS	
**Apomorphine**										
**Othman et al. (2025)** [36]	Double-blind crossover RCT<br><br>*Level 1b	Total: 50 (36 male)	63±10y	TBI/SDH<br>Non-TBI (cardiac arrest, other cardiovascular causes, SAH, IH, AIS, other causes)	Not specified (acute severely brain-injured patients in the ICU)	Coma<br>UWS<br>MCS	Clinical observation<br>FOUR<br>SECONDs<br>Automated pupillometry	Apomorphine: 2mg (subcutaneous)<br><br>Methylphenidate: 20mg (oral)	☙ No significant effect of apomorphine or methylphenidate on pupillary responses compared to placebo<br>☙ Significantly increased probability of improved arousal after methylphenidate<br>☙ Non-significant positive trend in arousal after apomorphine<br>☙ No significant between-group differences in changes in consciousness level<br>☙ Higher probability of improved arousal in patients with higher pupillary responsiveness<br>☙ Pupillary responses observed in 52.1% of patients at least once and in 25% with stricter thresholds, leading to CMD diagnosis in 14.6% of patients<br>☙ Non-significant association of CMD status, higher baseline FOUR scores, higher baseline consciousness level, survival to ICU discharge, non-anoxic injuries, and infratentorial lesions with numerically higher arousal responses	☙ Incomplete reporting of NIRS–EEG outcome<br>☙ Highly heterogeneous acute DoC cohort<br>☙ Underrepresentation of TBI aetiology due to the use of NIRS-EEG camps incompatible with external ventricular drains used in TBI<br>☙ Lack of confirmation of residual consciousness using fMRI- or EEG-based active paradigms<br>☙ Not yet established sensitivity of pupillometry to detect CMD<br>☙ Interchangeable use of the following terms: arousal, awareness, and consciousness<br>☙ Relying on investigator observation for detection of improved arousal rather than repeated standardised assessments, leading to potential missing information on worsening or additional positive outcomes after drug administration

										• Limited statistical power, necessitating replication
**Methylphenidate**										
**Othman et al. (2025)** [36]	Double-blind crossover RCT<br><br>*Level 1b	See above	See above	See above	See above	See above	See above	See above	See above	See above
**Moein et al. (2006)** [37]	Double-blind RCT<br><br>*Level 1b	Total: 80 (66 male)<br><br>• Methylphe e: 41 (33 male)<br>• Control: 39 (33 male)	• M phenidate: 33.6±16.7<br>• C l: 31.7±14.3	Moderate TBI (GCS: 9-12)<br>Severe TBI (GCS≤8)	/	• M phenidate: GCS 8.63±2.09<br>• C l: GCS 8.49±1.96	GCS<br>ICU stay<br>Hospital stay	0.6mg/kg daily (max. 20mg/dose) until discharge	• No significant GCS changes at discharge<br>• Shorter ICU and hospital stay in severe TBI<br>• Non-significantly shorter ICU and hospital stay in moderate TBI<br>• 4 mortalities in severe TBI (2 in methylphenidate and 2 in control group)<br>• Post-TBI seizure in 5 severe TBI (2 in methylphenidate and 3 in control group)	• Lack of a direct clue to link the drug's mechanism of action with the measured ICU and hospital length of stay<br>• Limited sample and centre size<br>• Marginal statistical significance
**Modafinil**										
**Talebi Kiasari et al. (2024)** [38]	Double-blind RCT<br><br>*Level 1b	Total: 18 (9 male)<br><br>• Modafinil: 9 (6 male)<br>• Control: 9 (3 male)	• M nil: 75.33±13.28<br>• C l: 70±12.06	Encephalopathy (COVID 19-related)	Hospitalisation to medication administration:<br>• M nil: 5.56 ±7.4d<br>• C l: 4.11±4.59d	GCS:<br>• M nil: 11.89±1.05<br>• C l: 11.67±1.32<br>Reduced level of consciousness stated; DoC states not specified	GCS<br>Blood laboratory parameters<br>Mortality	400mg/d (starting from 100mg, escalation every 2h), 1d (if 1-point GCS increase, continuation up to 4 weeks)	• Non-significantly slightly higher GCS scores after four doses of modafinil *vs.* placebo<br>• No significant differences in GCS after any dosing round<br>• No significant between-group differences in blood laboratory parameters, discharge, or mortality	• Small sample size, due to specific inclusion criteria (non-intubated patients with COVID19–related encephalopathy)<br>• Lack of additional paraclinical investigations (e.g., MRI, EEG, CSF analysis)<br>• Short duration of modafinil administration, limiting assessment of sustained or delayed effects<br>• Potential underestimation of treatment effects due to small sample size and brief exposure<br>• Limited demographic diversity, with patients drawn from a single

										geographic region, limiting generalizability<br>• Potential implementation and adherence biases, despite blinding and randomisation
**Zand et al. (2024)** [39]	Double-blind RCT<br><br>*Level 1b	Total: 85 (73 male)<br><br>• Modafinil: 41 (36 male)<br>• Control: 44 (37 male)	• M nil: 36.22±16.53<br>• C l: 36.11±15.76	• M ate TBI (N = 2)<br>• S TBI	4d<br><br>• M nil: 4d (range: 3-6.25d)<br>• C l: 4d (range: 3-6d)	GSC: 5-12;<br>DoC states not specified	GCS<br>ICU/hospital stay duration<br>Mechanical ventilation duration<br>Tracheostomy needs<br>GOS-E<br>APACHE II<br>ISS<br>Mortality	200mg/d, 7d	• Higher proportion of patients achieving clinically meaningful improvement (≥2-point increase in total GCS or a ≥1-point increase in motor GCS) with modafinil *vs.* placebo (54% & 56% *vs.* 32% & 34%)<br>• No significant difference in time to GCS improvement, early (≤7 days) GCS improvement, ICU/hospital stay, ventilation duration, ventilator-free days, intubation duration, tracheostomy rates, or mortality between groups<br>• No significant differences in GCS improvement among patients with or without neurosurgical intervention<br>• No significant differences in GOS-E scores at discharge, 1mo, or 3mo	• Single-centre study design limiting generalisability<br>• Small sample size, despite screening all ICU admissions over two years<br>• Underrepresentation of moderate TBI, likely due to strict inclusion criteria<br>• Consciousness assessment with GCS only<br>• Lack of continuous EEG monitoring, limiting detection of subclinical seizures<br>• Exclusion of patients with motor GCS score of <3, limiting applicability of findings to patients with more severe motor impairments
**GABAERGIC**										
**Zolpidem**										
**Rahat Dahmardeh et al. (2024)** [40]	Double-blind RCT<br><br>*Level 1b	Total: 80 (55 male)<br><br>• Zolpidem: 40 (26 male)<br>• Control: 40 (29 male)	37.7±16.3<br><br>• Z em: 36.7±16.5<br>• P o: 38.16±16.2	TBI<br>Ischaemia<br>Haemorrhage	Acute brain injury; TSI not specified	Mean GCS:<br>• Z em: 6.1±2.4<br>• P o: 5.9±1.7<br><br>DoC states not specified	GCS<br>Mechanical ventilation duration<br>Hospital stay<br>ICU discharge<br>Mortality	10mg BID, 14d	• No significant between-group difference in GCS, duration of mechanical ventilation, length of hospital stay, ICU discharge, or mortality<br>• No significant between-group GCS change based on aetiology	/

**Shafiee et al. (2022)** [28]	Single-blind RCT<br><br>*Level 2	See above	See above	See above	See above	See above	See above	See above	See above	See above
**Chatelle et al. (2014)** [41]	Double blind crossover RCT<br><br>*Level 1b	3 (1 male)	37,<br>38,<br>50	Anoxia	P1: 18mo,<br>P2: 12y and 7mo,<br>P3 : 7y	MCS	CRS-R<br>PET<br>MRI	10mg, single dose	- Emergence from MCS<br>- Increases in bilateral superior frontal gyri & right medial frontal cortex and with less conservative thresholds in left insula & middle frontal gyri after zolpidem *vs.* placebo	/
**Machado et al. (2014)** [42]	Double-blind crossover RCT<br><br>*Level 1b	8 (6 male; 2 paediatric)<br>8 age & sex-matched healthy controls	Mean or range not specified.<br>15, 15, 20, 21, 21, 22, 37, 54	Postanoxic encephalopathy (n=5)<br>Central transtentorial herniation (n=1)<br>Herpes simplex encephalitis (n=1)<br>Head trauma (n=1)	Variable, mean or range not specified<br>TSI reported per individual patient (range: 1.61-113.93d)	PVS	Clinical evaluation of arousal (yawns & hiccups)<br>EEG ECG<br>Physiological markers (Respiratory rate<br>Systolic (SBP) & Diastolic (DBP) blood pressure<br>Mean blood pressure (MBP)<br>Peripheral oxygen saturation (HbO_2, via pulse oximetry)	10mg, single dose	- Synchronised EEG phases (activated vs. non-activated) with HRV spectral dynamics in 1 patient after zolpidem; with yawns temporally associated with tachycardia (shorter R–R intervals)<br>- Yawns corresponding to a generalised EEG activation (higher frequency and greater spectral power across multiple bands)<br>- Eyes mostly open, more yawning episodes in 7 patients and hiccups in 1 patient for 53 minutes, compared to baseline and placebo<br>- Increased intensity and frequency of startle reactions and non-stereotyped limb/trunk movements<br>- Hemodynamic and respiratory indices (patients and controls) remaining within ±2 SD of baseline values<br>- 1 patient showing two significant decreases in respiratory rate and three short-term increases in cardiac output associated with high frequency HRV	- Small sample size

									- Reduction in high frequency in HRV<br>- Decreases in Z values of mean R-R intervals (faster heart rate) coincided with increases in Z values of low frequency/high frequency ratio<br>- Peaks in Stress Index in parallel with decreased high-frequency power and mean R-R interval<br>- PVS patients showing marked reductions in high frequency power in HRV, compared to no abnormal PSD fluctuations during placebo<br>- Healthy controls showing no abnormal spectral fluctuations under either condition<br>- Consistent HRV results with repeated zolpidem administration	
**Whyte et al. (2014)** [43]	Double blind crossover RCT<br><br>*Level 1	84 (57 male)	- N responders (median:34; range: 19-69)<br>- R nders (median: 36.5; range not specified: 19, 27, 28, 31, 36, 41, 42, & 44)	TBI<br>Ischemia<br>Hypoxia	- N responders (median: 47mo; range: 5-87mo)<br>- R nders (median: 33mo; range not specified: 6, 16, 21, 31, 35, 47, 86, & 99mo)	VS<br>MCS	DRS<br>CRS-R	10mg, single dose	- Increased movement, social interaction, command following, communication, and functional object use in 8 patients (~5%)<br>- No significant differences in age, TSI, or DRS between responders and non-responders<br>- Seven responders had TBI and 1 patient had hypoxic aetiology<br>- Responders were all men	- Low representativeness of the study sample due to convenience sampling<br>- Limited generalizability<br>- Qualitative report of families taken for inclusion of responders leading to lower sensitivity<br>- Over/underestimation of response to zolpidem due to difficulty distinguishing a drug effect from random performance variation in MCS patients<br>- Limited assessments to classify responders<br>- Not including patients responding to higher doses of zolpidem

**Whyte & Myers (2009)**[44]	Double-blind, crossover preliminary RCT<br><br>*Level 1	15 (10 male)	22-59	TBI<br>Anoxic encephalopathy<br>Toxic encephalopathy<br>Stroke	Range: 1mo-23y (median: 30.5mo)<br>>1y for 60% of patients	VS<br>MCS	CRS-R	10mg, single dose	- 1/15 patients (~7%) showed a clinically significant zolpidem response within 30min of zolpidem administration with a change of diagnosis from VS to MCS with spontaneous movements, exploratory eye movements, command following, visual tracking, attempts to grasp objects, kick a soccer ball, and wave<br>- No signs of consciousness or behaviours indicating MCS after placebo administration<br>- A replication with three 5mg zolpidem administration within 3h showed a less dramatic superiority of zolpidem *vs* placebo<br>- In 14 non-responders, no measurable behavioural effect after zolpidem with CRS-R scores remaining stable across zolpidem and placebo sessions	- Small sample size

**Brefel-Courbon et al. (2007)** [45]	Double-blind crossover RCT<br><br>*Level 4	1 (female)	48	Postanoxic en-cephalopathy	2y	Akinetic mutism	Motor tests<br>Language tests<br>FDG-PET<br>$H_2{}^{15}O$ PET	20mg<br>Three single doses	- Increased motor performance (standing up & walking)<br>- Increased language-related functions (reading, naming, & repeating), compared to complete mutism at baseline and during placebo<br>- FDG-PET: increased cerebral metabolism in postrolandic regions (+30%) and in lateral frontal cortex, anterior cingulate, medial and orbitofrontal cortices, and thalami (+25%), and decreases in bilateral rostral/subcortical regions (frontal cortex and thalamic areas), compared to placebo<br>- $H_2{}^{15}O$ PET: increased regional cerebral blood flow, mainly in anterior cingulate and orbitofrontal cortices in parallel with improved naming and repletion performance in naming task, compared to no significant change in regional cerebral blood flow & poor performance in naming task during placebo	/
**OTHERS**											
**Simvastatin**											
**Shafiee et al. (2021)** [46]	Double blind RCT<br><br>*Level 1	Total: 98 (66 male)<br><br>- Simvastatin: 49 (32 male)<br>- Control: 49 (34 male)	- Statin: 38±13<br>- Po: 36.6±11.4	TBI	/	GCS ≤8<br><br>8 patients in VS in simvastatin and 9 patients in VS in placebo group	GCS<br>Mechanical ventilation duration<br>ICU stay<br>Neurosurgery stay	Simvastatin 40mg/d, 10d	- Increased proportion of conscious patients at discharge and 1m after discharge in simvastatin group, compared to control group<br>- Decreased proportion of mild to moderate TBI in simvastatin vs. placebo group at discharge and 1m after discharge<br>- No severe TBI cases remained at discharge or 1m after discharge in either group<br>- Increased GCS scores in simvastatin group vs. placebo at discharge and 1m after discharge	- No assessment of neurological function using scales such as mRS, GOS, and BI<br>- Pattern of intracranial injury

									↪ Non-significantly lower length of mechanical ventilation, ICU and neurosurgery ward stay, and mortality rate in simvastatin vs. placebo group	

[1]In years, mean ± Standard Deviation (SD)/median/range; [2]Mean ± SD/median, range; [3]Before intervention; [4]Max./range; [5]Time interval between treatment administration and first signs of improvement. **Aetiology:** DAI: Diffuse Axonal Injury; SAH: subarachnoid haemorrhage; TBI: Traumatic Brain Injury. **Diagnosis:** CMD: Cognitive Motor Dissociation; MCS: Minimally Conscious State; UWS: Unresponsive Wakefulness Syndrome; (P)VS: (Persistent) Vegetative State. **Behavioural measures:** ABS: Agitated Behaviour Scale; APACHE: Acute Physiology and Chronic Health Evaluation; CNCS: Coma-Near Coma Scale; CRS-R: Coma Recovery Scale-Revised; CPC: Cerebral Performance Category; DRS: Disability Rating Scale; GCS: Glasgow Coma Scale; GOS(-E): Glasgow Outcome Scale(-Extended); FIM™: Functional Independence Measure; FOUR: Full Outline of Unresponsiveness; GOAT: Galveston Orientation and Amnesia Test; ISS: Injury Severity Score; KPS: Karnofsky Performance Scale; mRS: modified Rankin Scale. **Neuroimaging measures:** ECG: Electrocardiography; EEG: Electroencephalography; H-MRS: Proton Magnetic Resonance Spectroscopy; MRI: Magnetic Resonance Imaging; PET: Positron Emission Tomography. **Other:** BID: twice a day; BOLD: Blood Oxygen Level Dependent; CSF: Cerebrospinal Fluid; d: day; h: hour; IV: intravenous; LoE: Level of Evidence; min: minutes; mo: month; P: Patient; RCT: Randomised Controlled Trial; SOC: Standard of Care; w: week; y: year.

Note: Studies with other designs (e.g., case reports, retrospective studies) are presented in the Supplementary Table 2.

Supplementary Table 2. Overview of non-RCT studies included in this narrative review

Authors (year)	Type of study	Number of patients	Age[1]	Aetiology	Time since injury[2]	Diagnosis/Level of consciousness[3]	Outcome Measure(s)	Dose & duration[4]	Outcome(s)[5]	Time to onset[5]	Reported limitations
**Dopaminergic agents**											
**Amantadine**											
**Ayhan et al. (2025)** [47]	Retrospective descriptive study<br><br>*Level4	21	68.3y±17.6	Ischaemic stroke<br>Status epilepticus<br>Hypoxic & ischaemic encephalopathy<br>Dementia<br>SAH<br>Unknown (n = 4)	Mean: 16.6±9.5d post-ICU admission	Mean ICU GCS: 7.1±3.3; DoC states not specified	GCS<br>ICU stay length<br>Mechanical ventilation duration	150-600mg/d (median: 400mg/d)	↪ Consciousness recovery (>2-point ↑ in GCS score) in 16 (76.2%) patients<br>↪ Significant sex-related difference in consciousness recovery in the univariate model (all men and 45.4% of women showed recovery)<br>↪ No significant correlations with age, ICU stay, comorbidities, ventilation duration, dosing, or treatment duration<br>↪ Logistic regression not presenting a meaningful predictive model	Median: 3.5d; Range: 2-5.75d	↪ Retrospective study design & unavailability of some clinical parameters (e.g., sequential organ failure assessment at discharge, long-term consciousness monitoring)<br>↪ Limited statistical analyses<br>↪ Single-centre study design<br>↪ Limited generalizability to other ICU settings and prolonged DoC
**Blum et al. (2025)** [48]	Retrospective study (no control group)<br><br>*Level4	442	73.2y±10.7	AIS<br>ICH<br>SAH<br>Non-traumatic SDH<br>Meningitis/encephalitis	ICU patients; Time from weaning from mechanical ventilation to amantadine administration: 5.4±7.2d	Media GCS at admission: 3 (IQR: 3,14); DoC states not specified	GCS<br>RASS<br>SAPS II<br>NIHSS<br>mRS	200-400 mg/d, 3-5d	↪ 267 patients (60.4%) classified as responders<br>↪ Significantly lower baseline GCS, better premorbid functional status, fewer pathological cerebral imaging, fewer hypoxia-related changes, fewer EEG abnormalities, & shorter mechanical ventilation in responders<br>↪ Significantly lower mortality at discharge & follow-up and significantly better functional outcomes at follow-up in responders<br>↪ Higher premorbid mRS, pathological cerebral	1d	↪ Retrospective study design & potential selection bias<br>↪ Absence of a control group<br>↪ Variability in record-keeping quality<br>↪ Variability in amantadine dosing regimen<br>↪ Potential reporting bias in EEG and imaging findings<br>↪ Responder classification based on GCS and clinical documentation<br>↪ Lack of standardised DoC outcome measures (CRS-R, DRS, FOUR)<br>↪ Incomplete adverse event notes<br>↪ No long-term follow-up

									imaging, & cardiac arrest significantly associated with lower odds of response<br>• Significantly persisting higher CGS & RASS and lower SAPS scores after amantadine in responders,<br>• Significantly more frequent seizures and delirium in non-responders<br>• Stacking ensemble learning model showing the most optimal predictive performance (accuracy = 64.5%, F1-score = 61.3%)		
**Hintze et al. (2022)** [49]	Retrospective cohort study<br><br>*Level3	142<br><br>• adine: 43<br>• nil: 13<br>• 86	• ntadine: 34 (range: 25-56)<br>• finil: 41 (range: 25-61)<br>• : 34 (range : 26-53)	TBI	Amantadine: 13d (range: 8-19)	GCS: Amantadine: range 7-11	GCS	50-200mg/d, 6-16d	• No improvement in GCS scores or hospital length of stay with amantadine (or modafinil) compared to SOC	/	• Retrospective data<br>• Single-centre design<br>• Selection bias (clinical selection)<br>• Small sample size<br>• No functional outcome measures<br>• No information on decision-making for initial drug dose and increase<br>• Majority of patients with a blunt mechanism of injury
**Rülh et al. (2022)** [50]	Observational cohort study (pooled patient data of five single-centre studies)<br><br>*Level3	184<br><br>• adine: 84<br>• l: 100	• ntadine: 76 (range: 69–80)	IS<br>ICH<br>SAH<br>CAMBSE	Amantadine: 10.74d (range: 7.87–14.09)<br>Control: 12.41d	Amantadine: GCS scores 5 (4-5)<br>Control: GCS scores 4 (3-6)	GCS	≥ 100mg/d	• ↑GCS scores with amantadine<br>• Rate of epileptic seizures numerically ↑ after amantadine initiation	/	• Biased by indication and timing of treatment initiation<br>• Single-centre design<br>• Underpowered to evaluate treatment effects on mortality, seizures and delirium<br>• Practical limitations of the GCS with intubated patients

			☙ ol: 65 (range: 57–74)		(range: 7.70–15.84)						
**FitzGerald et al. (2021)** [51]	Single-case experimental design<br><br>*Level4	1	65	ABI	6y	/	Functional tasks	100-200mg/d	☙↑ mean grades (tasks) and ↓ mean sleep duration with amantadine<br>☙↓ mean grades (tasks) and ↑ afternoon sleep duration after amantadine withdrawal	/	☙ No placebo and/or blinding<br>☙ Possible learning effect
**Hadgu et al. (2021)** [52]	Retrospective cohort study<br><br>*Level3	79	40.6±15.7	TBI	8.2±10.5d	GCS: 10.4±3.4	GCS	9-267mg/d, 12.1±9.3d	☙↑ GCS scores	/	☙ Limited external and internal validity<br>☙ Missing or incomplete data<br>☙ No comparator group<br>☙ Comorbid conditions not assessed<br>☙ Short assessment period
**Leclerc et al. (2021)** [53]	Retrospective cohort study<br><br>*Level3	87<br><br>79 patients in effectiveness evaluation<br>☙ adine: 62<br>☙ nil: 15<br>☙ 24	66 (range: 56-73)<br><br>☙ ntadine: 66 (range: 55-74)<br>☙ finil:<br>☙ (range: 61-71)<br>☙ 64 (range: 64-67)	IH Ischaemic stroke SAH	7d (range: 4.25-12.75)	/	GCS Behavioural examination (wakefulness, participation)	100-200mg/d,<br><br>50mg every two days if impaired kidney function or 100-200mg weekly doses if haemodialysis	☙34/62 patients receiving amantadine monotherapy<br>☙No responder in modafinil monotherapy<br>☙8/24 patients receiving both treatments considered as responders<br>☙Responders more frequently discharged home or to acute rehabilitation compared to non-responders	3d (range: 2-5)	☙ Small sample size (no analysis by stroke type and location)<br>☙ Reliance on caregiver notes<br>☙ No control for changes in the quality of documentation over time<br>☙ Variable frequency of assessments from patient to patient<br>☙ No untreated control group<br>☙ Inconsistent follow-up after starting or changing neurostimulant medication

**Alkhachroum et al. (2020)** [54]	Retrospective observational study<br><br>*Level3	44	/	ABI	/	/	EEG	/	• Association between sleep features/posterior dominant rhythm after amantadine initiation and recovery of command following<br>• ↑ levels of anterior forebrain corticothalamic integrity after amantadine initiation for patients with later behavioural recovery	/	• Small sample size<br>• No comparative group (without amantadine treatment)<br>• Short duration of treatment<br>• Command following assessed as surrogate for consciousness recovery
**Bender Pape et al. (2020)** [55]	Pilot alternate treatment, within-subject, baseline-controlled trial<br><br>*Level4	4 (1 paediatric)	21±3.3	TBI	6.1y	UWS MCS-	DOCS	200mg daily, 28d	• Meaningful neurobehavioural gains (↑DOCS total measures) after rTMS, amantadine, and rTMS+amantadine<br>• Auditory-language gains after rTMS, doubled when rTMS preceded rTMS+amantadine, with gains reflected in resting state functional connectivity for language, salience, and sensorimotor networks	/	• Small sample size<br>• Non-blind evaluators<br>• Not powered to determine efficacy (possibility of type 2 error)
**Gao et al. (2020)** [56]	Retrospective controlled study<br><br>*Level3	46<br><br>• adine: 12<br>• l: 34	• ntadine: 53±7.98<br>• ol: 51±15.1	Haemorrhage	30-90d	Amantadine: CRS-R scores 3.5 (range: 2-7)<br>Control: CRS-R scores 3.5 (range: 2-7)	GOS	200-400mg/d, 2-4mo	• ↑ GOS scores in amantadine group (not significant)<br>• Faster recovery in amantadine group	3-7d	• Small sample size<br>• Unknown effects of other adjuvant therapies<br>• Inconsistent starting time and duration of medication
**Lee et al (2020)** [57]	Retrospective case-control study<br><br>*Level3	84<br><br>• adine: 42<br>• adine &	60.2±17.1	Stroke (ischemic/haemorrhagic)<br>TBI<br>HBI<br>Metabolic	24.9±25.6w<br>Amantadine: 25.6±29.3<br>Amantadine	UWS<br>MCS-<br>MCS+	CRS-R	100-200mg/d, 4w	• ↑ CRS-R scores in the dual regimen group compared with the single regimen group	/	• Demographic heterogeneity between groups<br>• Selection of patients by physician (selection bias)<br>• Dose of cerebrolysin lower in previous studies<br>• No assessment of persisting effect of cerebrolysin after discontinuation

		cerebrolysin: 42		encephalopathy Leukoencephalopathy	and cerebrolysin: 24.2±21.6						• Patients assessed only once before and once after drug administration
**Zão et al. (2020)** [58]	Case report<br><br>*Level4	1	16	TBI	17w	UWS	CRS-R<br>GSC<br>FIM<br>RLA	100-200mg/d, 9w	• ↑ CRS-R, GSC, FIM, RLA scores (clinical and functional improvement even after amantadine discontinuation)<br>• ↑ neuromotor deficit<br>• ↑ active participation in care	3w	/
**Chen et al. (2019)** [59]	Case report<br><br>*Level4	1	52	TBI	/	UWS	fMRI	200-400mg/d, 3mo	• ↑ brain activity compared to before amantadine<br>• Activation around left insular regions during stimulation by familiar voice<br>• Activity in left temporal and bi-occipital cortices during stimulation by familiar/unfamiliar voice<br>• Activity in bilateral frontal and parietal cortices during motor-imagination task	/	/
**Lehnerer et al. (2017)** [60]	Case report<br><br>*Level4	1	36	SAH	10mo	MCS	CRS-R	100-300mg/d	• ↑ CRS-R scores (to the maximum of 23 points)	16d	/
**Estraneo et al. (2015)** [61]	Single-case experimental design<br><br>*Level4	1	57	Cerebral cyst	16mo	MCS	CRS-R<br>EEG	100-200mgd, 16w	• ↑ CRS-R scores (with emergence from MCS) and EEG background activity (predominant alpha activity) during amantadine treatment<br>• On-off effect with return to baseline results after amantadine discontinuation	3d	/
**Avecillas-Chasin & Barcia (2014)** [62]	Case report<br><br>*Level4	1	49	Cerebral artery rupture (during embolisation)	/	MCS	CRS-R	200-400mg/d	• ↑ CRS-R scores<br>• ↓ alertness level after temporary discontinuation	3d	/

**Reynolds et al. (2013)** [63]	Retrospective cohort study<br><br>*Level3	16<br><br>ꕥ A<br>adine: 6<br>ꕥ M<br>phenidate: 8<br>ꕥ<br>2	ꕥ<br>ntadine: range: 38-78<br>ꕥ<br>ylphenidate: Range: 23-78	Cardiac arrest	Amantadine: 4-35d<br>Methylphenidate: 4-10d	GCS scores: 3-6<br>FOUR scores: 0-8	Command following<br>Survival to hospital discharge<br>CPC<br>mRS	**Amantadine**: range: 100-200mg/d<br>**Methylphenidate**: 10-60mg/d	ꕥRecovery of command following prior to discharge for 6 patients<br>ꕥNeurostimulants improved rate of following commands, survival to hospital discharge, and distribution of CPC and mRS scores	1-18d	ꕥ Small sample size<br>ꕥ Retrospective data collection<br>ꕥ Limited to in-hospital phase
**Schnakers et al. (2008)** [64]	Case report<br><br>*Level4	1	23	Cardiac arrest (ventricular fibrillation)	2y	MCS	CRS-R<br>FDG-PET<br>Actimetry	200mg BID, 6w (with a 6w pause between)	ꕥ↑ CRS-R scores<br>ꕥ↑ metabolic activity<br>ꕥ↑ motor activity	/	/
**Patrick et al. (2006)** [34]	Case report<br><br>*Level4	1	16	TBI	6w	GCS 3<br>CNCS 2.8/4 (moderate coma, almost marked coma); DoC states not specified	CNCS<br>WNSSP<br>DRS<br>RLAS	50-200mg/d	ꕥImproved from baseline moderate coma (CNCS: 2.8/4, WNSSP: 20/113, DRS: 24/29, RLAS III) to peak-dose recovery with no coma (CNCS: 0.36), reduced disability (DRS: 14), functional responsiveness eligible for rehabilitation (WNSSP: 71), and higher consciousness levels (RLAS IV–V), with gains maintained and modestly enhanced on WNSSP after medication tapering	/	/
**Hughes et al. (2005)** [65]	Retrospective cohort study<br><br>*Level3	123<br><br>ꕥ A<br>adine: 28<br>ꕥ C<br>l: 95	38±19	TBI	6w	ISS 39.4±8.7	Emergence from coma (RLA)	200-400mg/d, 25±20d	ꕥ46.4% of cases emerged from coma compared to 37.9% of controls	/	ꕥ Small sample size<br>ꕥ No consistent data recording<br>ꕥ Unknown criteria for prescribing amantadine<br>ꕥ Selection and treatment biases<br>ꕥ Lack of follow-up for subjects returning to acute/referring facility<br>ꕥ Cases and controls not truly comparable

											• Results not truly representative of treatment effectiveness in this population
**Whyte et al. (2005)** [66]	Longitudinal observational cohort study<br><br>*Level2b	124<br><br>Amantadine: 47	34.2±13.3	TBI	/	DRS scores 22.97±2.09	DRS	/	• Improved DRS scores with amantadine	/	• Data extracted from medical records based on availability<br>• Patients receiving several drugs simultaneously or successively<br>• Incomplete information provided on treatment (particularly dosage and duration)
**Saniova et al. (2004)** [67]	Retrospective pilot study<br><br>*Level3	74<br><br>• adine: 41<br>• l: 33	Amantadine: 42.12±16.8<br>Controls: 43.91±18.45	TBI	3d	Amantadine: GCS scores 4.74±2.26<br>Control: GCS scores 4.70±2.14;<br>DoC states not specified	GCS	600mg/d, 3d	• ↑ GCS scores and lower mortality rate with amantadine	/	• Retrospective data<br>• Small series
**Patrick et al. (2003)** [68]	Retrospective study<br><br>*Level3	10 (9 paediatric)<br><br>• adine: 1	8-19y	TBI	52.5d (range: 22-112d)	Low response state; DoC states not specified	WNSSP	100-400mg/d, 6-132d	• ↑ WNSSP scores with dopamine-enhancing medications	/	• Heterogeneous group by age, aetiology of brain injury and dopamine-enhancing drug selected<br>• Retrospective data

		• adine/bromocriptine: 1<br>• adine/pramipexole: 1								
**Levodopa**										
**Roy et al. (2021)** [69]	Case report<br><br>*Level4	1	60	COVID-19 encephalopathy<br>Stroke	47d	/	Behavioural examination<br>Follow-up calls	Levodopa 300mg/carbidopa 75mg/d, 30d	• ↑ wakefulness, alertness, movements (left hemiparesis), command following, feeding, communication, participation in physiotherapy, occupational therapy and speech therapy with levodopa/carbidopa and modafinil, as well as ventilator weaning, tracheostomy removal, walking and discharge to home	2d	No control of confounding factors
**Bancalari et al. (2018)** [70]	Case report<br><br>*Level4	1	62	IVH<br>Hydrocephalus	/	MCS	CRS-R	Levodopa 300mg/carbidopa 75mg/d	• Spontaneous eye opening<br>• ↑ hand movement, muscular strength in distal upper and lower limbs and communication abilities	4d	/
**Ugoya et al. (2010)** [71]	Prospective study<br><br>*Level2b	11 (2 paediatric)	Range: 10-75	TBI<br>ICH<br>CO<br>SE<br>Intoxication<br>Unknown	/	PVS with parkinsonism features	Aspen criteria for MCS	Levodopa 200-300mg/carbidopa 50-75mg/d, 2-5mo	• ↑ response to command and reciprocal interaction<br>• complete recovery and discharge	Within 10d	• Observational study<br>• Limited control of confounding factors<br>Financial and cultural constraints (neuroimaging investigations and autopsy reports)
**Matsuda et al. (2005)** [72]	Retrospective case series<br><br>*Level3	5 (2 paediatric)	Range: 7-51	TBI	3-22mo	PVS<br>MCS	Behavioural examination<br>MRI<br>EEG	Levodopa 200-450mg/carbidopa 20-30mg or	• Rapid recovery from PVS or MCS with the treatment despite concomitant diffuse axonal injury involving the cerebral peduncle,	4d to 1.5 mo	/

								benserazide 50-75mg/d (adult), 2 mg/kg daily (children)	dorsolateral midbrain, pallidum, or corpus callosum<br>❧↑ alpha activity and attenuation of delta or theta wave activity after the start of treatment<br>❧↓ rigidity/spasticity and akinesia symptoms		
**Krimchansky et al. (2004)** [73]	Prospective study<br><br>*Level2b	8	Range: 20-50	TBI	30-210d	VS	Behavioural criteria	Levodopa 125-750mg/carbidopa 12.5-75mg/d	❧Recovery of moving a limb on request, performing more than one command, opening the mouth on request and reciprocal interaction with gradual dose increase	Within 2w	/
**Matsuda et al. (2003)** [74]	Cases series<br><br>*Level4	3 (1 paediatric, same patient as in Matsuda et al, 2005)	Range: 14-51	TBI	3-12mo	PVS	Behavioural examination<br>MRI	Levodopa 200-450mg/carbidopa 30mg or benserazide 50-75mg/d, 1y to indefinitely	❧↑ neurological status (including post-traumatic parkinsonism): ↓ involuntary movements and spasticity/rigidity/akinesia, eyes turning towards voice, simple verbal commands, walking to school, communication	4-9d	/
**Patrick et al. (2003)** [68]	Retrospective pilot study<br><br>*Level3	10<br><br>Levodopa: 1 (paediatric)	Range: 8-19	TBI<br>Encephalopathy<br>Stroke	52.5d (range: 22-112d)	Low response state; DoC states not specified	WNSSP	100/25mg/d, 15d	❧↑ WNSSP scores with dopamine-enhancing medications	/	❧ Heterogeneous group by age, aetiology of brain injury and dopamine-enhancing drug selected<br>❧ Retrospective data
**Apomorphine**										
**Sanz et al. (2024)** [75]	Open-label controlled study<br><br>*Level3	13<br><br>❧ A orphine: 6<br>❧ C l (SOC): 7	Range: 26-52<br><br>❧ morphine: 44 (36-52)	TBI<br>Non-TBI	❧ morphine: 93d (70-96d)<br>❧ ol: 100d	UWS<br>MCS	CRS-R<br>DoC-feeling<br>WHIM<br>DRS<br>hdEEG<br>FDG-PET	1-6mg/h for 12h/d, 30d	❧ Significant effect of group (apomorphine *vs.* control) and study phase (treatment & washout *vs.* baseline) on behavioural diagnosis<br>❧ More new conscious and language-related behaviours during treatment and washout in apomorphine *vs.* control group	3mo	❧ Limited sample size, reducing statistical power to detect effects, particularly for brain activity outcome measures; decreasing precision of mixed-effects model estimates, reflected by wide confidence intervals, increasing the sensitivity to individual patient variations

			• ol: 40 (26-49)		(95-104d)				• Non-significant between-group difference in CRS-R index<br>• Higher DoC-feeling scores reported by healthcare staff and family members after apomorphine *vs.* control<br>• Significant post-treatment ↑ (*vs.* baseline) in hdEEG whole brain and alpha-band parietotemporal connectivity as well as ↑ in whole brain and frontal alpha-band network participation coefficient<br>• No significant post-treatment changes (*vs.* baseline) in hdEEG spectral power, number of connections, or other frequency bands<br>• Significant post-treatment ↑ (*vs.* baseline) in global metabolism, reflected by a mean +13.8% improvement		• Repeated-measures issues, with high intraclass correlation coefficients indicating strong within-patient interdependency, and reducing the amount of independent information available for statistical analyses<br>• Limited power to detect between-group baseline differences, particularly in diagnosis and aetiology<br>• Limited power potentially explaining non-significant group effect on CRS-R index<br>• Availability of neuroimaging and neurophysiology only for the apomorphine group<br>• Potential contribution of spontaneous recovery to positive outcomes<br>• Apomorphine dosing regimen extrapolated from Parkinson's disease literature and limited brain injury data due to lack of formal dose-finding studies<br>• Lack of individual drug concentration or absorption monitoring<br>• Inherent potential bias in open-label design with therapists and families aware of treatment allocation<br>• Potential bias in subjective measures (e.g., DoC-feeling)
**Fridman et al. (2010)** [76]	Open label multicentre pilot study<br><br>*Level4	8	Range: 18-41	TBI (DAI)	46-104d	UWS MCS	CNCS DRS	2mg/h to 8mg/h during 12h per d (5mg/ml), duration not specified	• ↑ CNCS and DRS scores up to at least 1y, even after treatment discontinuation	Within 10d	• No dissociation between apomorphine-induced response and spontaneous recovery

**Fridman et al. (2009)** [77]	Case report<br><br>*Level4	1	25	TBI (DAI)	104d	MCS	CNCS<br>GOS-E<br>DRS<br>MRI(DTI)	2mg/h to 8mg/h during 12h per d (5mg/ml), 76d	• ↑ CNCS, DRS and GOS-E scores over time from baseline to 2y post-treatment<br>• ↓ thalamocortical and corticothalamic projections in compared to healthy controls at 2y post-injury with remaining projections in the normal range	Within 24h	• Open-label design<br>• No control of confounding factors related to the standard rehabilitation program
**Methylphenidate**											
**Reynolds et al. (2013)** [63]	Retrospective cohort study<br><br>*Level3	See above	See above	See above	See above	See above	See above	See above	See above	See above	See above
**Kim et al. (2009)** [78]	Prospective study<br><br>*Level2	14 (1 paediatric)	Range: 15-65	Stroke/ICH<br>SAH<br>IVH<br>TBI	24-78d	GCS scores 7.79±1.12; DoC states not specified	GCS<br>FDG-PET	0.3mg/kg per d	• ↑ GCS scores and cerebral glucose metabolism (most evident in left praecuneus, right posterior cingulate gyrus, right retrosplenial cortex and right inferior parietal cortex)	/	• Heterogeneous aetiology of impaired consciousness<br>• No detailed evaluations of consciousness (GCS not sensitive to reflect visual, auditory, and motor functions recovery)<br>• No evaluation of initial injury severity<br>• Lack of control data
**Patrick et al. (2003)** [68]	Retrospective pilot study<br><br>*Level3	10 (2 paediatric)<br><br>Methylphenidate: 4	Range: 8-19	TBI<br>Encephalopathy<br>Stroke	52.5d (range: 22-112d)	Low response state; DoC states not specified	WNSSP	10-80mg/d, 7-47d	• ↑ WNSSP scores with dopamine-enhancing medications	/	• Heterogeneous group by age, aetiology of brain injury and dopamine-enhancing drug selected<br>• Retrospective data
**Bromocriptine**											
**Munakomi et al. (2017)** [79]	Prospective cohort study<br><br>*Level2	36 (3 paediatric)	/	TBI (mild, moderate and severe)	/	MCS	GCS<br>FND<br>BMC motor score<br>FIM	7.5mg/d (adult), 3.75mg/d (children)	• Accelerated arousal<br>• ↑ GOS scores	4d	• Small sample size<br>• No long-term follow-up<br>Confounding bias

**Patrick et al. (2003)** [68]	Retrospective pilot study<br><br>*Level4	10<br><br>Bromocriptine: 1 (paediatric)	Range: 8-19	TBI	52.5d (22-112d)	Low response state; DoC states not specified	WNSSP	0.125-20mg/d, 31d	ଓ↑ WNSSP scores with dopamine-enhancing medications	/	ଓ Heterogeneous group by age, aetiology of brain injury and dopamine-enhancing drug selected<br>Retrospective data
**Passler & Riggs (2001)** [80]	Retrospective study<br><br>*Level4	5 (3 paediatric)	18.9±4.7	TBI	33-50d	VS	DRS<br>FIM<br>CRS-R<br>BRISC	2.5-5mg/d, 2-6mo	ଓ↑ DRS, FIM, CRS-R and BRISC scores	/	ଓ No control of confounding factors related to recovery
**Modafinil**										
**Hintze et al. (2022)** [49]	Retrospective cohort study<br><br>*Level3	142<br><br>ଓ adine: 43<br>ଓ nil: 13<br>ଓ 86	Amantadine: 34 (25-56)<br>Modafinil: 41 (25-61)<br>SOC: 34 (26-53)	TBI	Modafinil: 14d (range: 9-19d)	Modafinil: GCS scores 8 (range: 6-10)	GCS	100-200mg/d, 6-24d	ଓNo improvement of GCS scores or hospital length of stay with amantadine (or modafinil) compared to SOC	/	ଓ Retrospective data<br>ଓ Single-centre design<br>ଓ Selection bias (clinical selection)<br>ଓ Small sample size<br>ଓ No functional outcome measures<br>ଓ No information on decision-making for initial drug dose and increase<br>ଓ Majority of patients with a blunt mechanism of injury
**Roy et al. (2021)** [69]	Case report<br><br>*Level4	1	60	COVID-19 encephalopathy<br>Stroke	47d	/	Behavioural examination<br>Follow-up calls	200mg/d, 30d	ଓ↑ wakefulness, alertness, movements (left hemiparesis), command following, feeding, communication, participation in physiotherapy, occupational therapy and speech therapy with levodopa/carbidopa and modafinil, as well as ventilator weaning, tracheostomy removal, walking and discharge to home	2d	ଓ No control of confounding factors
**Cross et al. (2020)** [81]	Retrospective study<br><br>*Level3	199<br><br>ଓ nil: 72<br>ଓ l: 127	72.9±14	Stroke	7.56±6.02d	Modafinil: NIHSS scores 13.5 (15)	NIHSS<br>mRS	50-200mg/d, 8.5±13.3d	ଓNonsignificant ↑ NIHSS scores and mRS with the treatment<br>ଓ↑ odds of favourable discharge disposition with the treatment (across all	/	ଓ Lack of uniform protocol (time to first dose and treatment dose)<br>ଓ No objective measure fatigue/lethargy (documentation by staff)<br>ଓ Possible patient selection bias

						Control: NIHSS scores 11 (10)			subgroups of patients, except severe stroke)<br>•		• No long-term follow-up
**Leclerc et al. (2021)** [53]	Retrospective study<br><br>*Level3	See above	See above	See above	See above	See above	See above	See above	See above	See above	See above
**Mo et al. (2018)** [82]	Retrospective, cohort study<br><br>*Level3	60	67±10.4	Neurological<br>Respiratory<br>Cardiovascular<br>Liver diseases	/	/	GCS<br>SAS	170mg±41mg/d, 6-12d	• Nonsignificant ↑ GCS scores	Within 6h	• No control group<br>• Unmeasured confounding factors<br>• Duration of the study limited to 48 hours before and after treatment<br>• No examination of the treatment effect on ICU delirium<br>•
**Formica et al. (2017)** [83]	Case report<br><br>*Level4	1	15	Tumour resection	7mo	DRS 19<br>CNCS 0<br>LOCFAS 3	DSR<br>LOCFAS<br>CNCS<br>PSG	200mg/d	• ↑ DRS, LOCFAS and CNCS scores<br>• ↑ diurnal wakefulness and nocturnal sleep with partial restoration of circadian rhythm<br>• Consistent emotions with environment, oral feeding, recognition of objects, images and graphemes, and implementation of alternative communication<br>• Persistent quadriplegia	1d	• /
**Dhamapurkar et al. (2016)** [84]	Retrospective pilot study<br><br>*Level3	24	Range: 29-68	TBI<br>Non-TBI	6-45mo	VS<br>MCS	CRS-R<br>WHIM<br>Sleep-wake cycle<br>Behavioural charts	100-400mg/d, 4-72w	• ↑ wakefulness, awareness, concentration, tracking and following commands<br>• ↑ WHIM scores between pre-intervention and intervention (particularly in TBI patients)	/	• Heterogeneous clinical presentation<br>• No control of confounding factors<br>• No placebo

**Rivera (2005)** [85]	Case report<br><br>*Level4	1	77	Transcallosal surgery (tumour, haematoma, ventriculomegaly)	11d	GCS score 8	GCS	400mg/d, 1mo	↪ Rapid improvement of GCS scores	3d	/
**Selegiline**											
**Masotta et al. (2018)** [86]	Open pilot study<br><br>*Level4	10	42.4±19.1	Vascular Anoxia TBI	19±23.2mo	VS MCS	CRS-R	5-10mg/d, 10w	↪ ↑ clinical diagnosis in four patients after treatment and at 1 month of follow-up (↑arousal level only in three patients)<br>↪ Treatment stopped in three patients because of possible side effects	2w	↪ Small sample size<br>↪ No control group<br>↪ -No neuroimaging structural and functional integrity assessment of cerebral dopaminergic networks
**GABAergic Agents**											
**Zolpidem**											
**Li et al. (2024)** [87]	Case report<br><br>*Level4	1	37	TBI	>3mo Not precisely specified	MMSE=7; DoC states not specified	MMSE Task-based fNIRS	10mg, single dose	↪ Reproducible, transient cognitive and language improvements, enabling communication, arithmetic calculation and game playing after zolpidem, beginning ~1h after administration and lasting 2–3h<br>↪ ↑ MMSE score from 7 to 18 after zolpidem<br>↪ Improved task accuracy across arithmetic calculation, face recognition, and picture naming fNIRS tasks after zolpidem<br>↪ Widespread bilateral brain activation during tasks before zolpidem *vs.* ↓ right-hemisphere activity and ↑ left-hemisphere activation after zolpidem	1h	↪ Case report of one patient, not generalizable<br>↪ Potential confounding factors: lack of a control

									• Resting-state fNIRS showing parietal connectivity before zolpidem *vs.* frontal connectivity after zolpidem<br>• Family reporting temporary cognitive and language improvements at follow-up and no tolerance after long-term use		
**Smack et al. (2024)** [88]	Case report<br><br>*Level4	1	53	Post-operation (resection of aggressive neck sarcoma) loss of consciousness and later posterior medial right cerebellar stroke	28d	MCS	CRS-R<br>RASS<br>EEG	5mg BID	• Marked improvement in attention and function with ↑ CRS-R score from 10 to 15, with improved alertness and communication<br>• ↑ RASS score from -10 to -1<br>• Continued difficulty with sustained attention<br>4d later:<br>• Further ↑ of CRS-R to 22 and normalisation of RASS score to zero, 4d later<br>• Attempted verbal communication (limited by tracheostomy), effective written communication with intact sentences<br>• Intact cognitive functioning (orientation, attention & logical thinking, memory)	1d	/
**Zhou et al. (2024)** [89]	Case series<br><br>*Level4	2	24,<br>23	TBI	8 mo,<br>6 mo	MCS+<br>eMCS	CRS-R	10mg, single dose	• Recovery of functional communication in one patient and intelligible verbalisations in both patients	1h	• Positive bias with the same person behaviourally assessing the patient pre- and post-zolpidem<br>• Behavioural assessment only<br>-No neuroimaging assessment
**Gao et al. (2023)** [90]	Prospective cohort study<br><br>*Level1b	31 prolonged DoC, 11 healthy controls	46.0±13.3	Anoxia (n = 15, 48.4%)<br>TBI (n = 8, 25.8%)<br>Cerebrovascular diseases	29-84d; (median = 37d)	UWS<br>MCS	EEG<br>CRS-R	10mg, single dose	• Beta band power after zolpidem administration predict recovery outcomes<br>• ↑ EEG beta-band power induced by zolpidem predict improved consciousness 6mo after enrolment (18 out of 31 patients improved)	1h	• EEG recording only once during the week following the hospital admission<br>• No post-zolpidem evaluations with neuroimaging<br>• Administration of only one dose of zolpidem<br>• Small sample size<br>• Single-centre study

				(n = 7, 22.6%)<br>Metabolic encephalopathy (n = 1, 3.2%)					• ↑ consciousness as assessed by CRS-R in 18 (58.1%) patients 6mo after zolpidem intake		• Uneven distribution of aetiologies
**Hao et al. (2022)** [91]	Prospective observational cohort study<br><br>*Level3	8	47.9±9.9	Trauma (n=3)<br>Stroke (n=4)<br>Anoxia (n=1)	3-12mo (mean: 4.5mo)	MCS+<br>MCS-<br>VS	CRS-R<br>EEG	10mg, single dose	• P1:↑CRS-R score from 12 to 22<br>• P2:↑CRS-R score from 7 to 12<br>• P3:↑CRS-R score from 7 to 8<br>• P4:↑CRS-R score from 7 to 12<br>• The other four patients: drowsiness after zolpidem	/	• No reports on EEG features of responders to zolpidem due to not having a responder<br>• Small sample size<br>• Heterogeneity of patients<br>• Only analysing EEG signals around 20-40 minutes after zolpidem administration
**Desmarais et al. (2021)** [92]	Case report<br><br>*Level4	1	75	PIHV	2d	/	FIM<br>MRI	2.5mg/d (bedtime; overall 6 administrations)	• ↑ motor function arousal, verbalisation, and command following<br>• After 3 doses, patient fully alert and oriented<br>• ↑ mobility, self-care, comprehension, and problem solving,<br>- ↑ regional oedema	12h	/
**Zhang et al. (2021)** [93]	Retrospective observational study<br><br>*Level3	146<br><br>Zolpidem: 87<br>Lorazepam: 48<br>Both: 11	36±15	Haemorrhagic stroke (n=11)<br>TBI (n=87)<br>Anoxia (n=48)<br>*11 non-responding stroke patients excluded	Median = 62d (IQR: 22,246d)	VS/UWS, MCS, eMCS	CRS-R	**Zolpidem**: 5-15mg; once, BID, TID, QD<br>**Lorazepam**: 1-4mg; once, BID, TID, QD	• 5 zolpidem and 6 lorazepam responders showing the following improvements to each medication:<br>• **Zolpidem:** ↑ verbalisation, arousal, attention, processing speed; more accuracy in answering yes/no questions; reproducible movement to command; more responsive and interactive.<br>• **Lorazepam:** ↑ arousal, alertness, verbalisation; ↑ cognitive ability; less restlessness, ↑ attention and engagement with environment, initiation, processing time; positive and	30-60min	• Uncontrolled practice setting and no pre-planned clinical management pattern due to the retrospective design<br>• Possibility of over/underestimation of the drug response due to the possibility of the confounding factors influencing the results<br>• Small sample size<br>• Impossibility of identifying responsiveness phenotypes

									spontaneous affect; participation in therapy; spontaneous movement<br>ↂ11 patients underwent both trials, but none showed positive response to both.		
**Khalili et al. (2020)** [94]	Prospective study<br><br>*Level4	12	36	TBI	Mean: 13mo	VS	GCS motor subscale SPECT	10mg BID, 2w	ↂ Six patients' diagnosis changed to MCS<br>ↂ ↑ motor function<br>ↂ Non-significant change in relative brain perfusion	2w	ↂ Small sample size
**Sripad et al. (2020)** [95]	Case report<br><br>*Level4	1	35	TBI	8y	Fully conscious after emerging from VS	MEG sMRI	10mg, single dose	ↂMinor subjective improvements reported by patient's family despite substantial improvements in speech, movement, gait, and coordination 2y before this study<br>ↂ↓ power in 4-12 Hz frequency band and ↑ power in 15-43 Hz frequency bands<br>ↂConnectivity difference in 15-43 Hz frequency band	30min	ↂ Possibility of recovery as a function of time since injury<br>ↂ Small sample size
**Arnts et al. (2020)** [96]	Single-case experimental study<br><br>*Level4	1	29	Severe hypoxic-ischemic brain injury	8y	Hyporesponsiveness with akinetic mutism; No formal diagnosis	EEG MEG MoCA	10mg, single dose	ↂReturn of awareness of the environment, functional speech, and spontaneous intentional object use<br>ↂSupported walking<br>ↂMoCA score ↑ from 0 to 13/30,<br>ↂMore wakefulness with generalised beta activity and some frontal theta/delta<br>ↂ↓ theta and alpha power in frontoparietal cortices<br>ↂ↑ relative beta and gamma power in frontotemporoparietal regions based on EEG and in all cortical regions based on MEG<br>ↂA shift from high levels of theta power in temporo-	30min	ↂ Performance confounds<br>ↂ Lacking a placebo group<br>ↂ Single-patient design

									occipital regions to high levels of alpha power<br>↓ beta band functional connectivity based on EEG and MEG<br><br>Single dose administrations for longer than 5 days led to no clinical improvements or sedation<br>- ↑ administrations (10 mg TID) led to ↓ effectiveness with ↓ talking and moving abilities during a narrower time window		
**Delargy et al. (2019)** [97]	Case report<br><br>*Level4	1	44	SAH	10mo	MCS	CRS-R WHIM MATAD OC	10mg, 1x/w, 8w combined with an IDT protocol	Temporary emergence from MCS<br>↑ arousal, visual attention, and motor skills<br>Spontaneous, appropriate verbalisation and communication<br>Emergence of the ability to sing familiar songs, use familiar objects, and make choices	30min	Not an RCT<br>Single-patient design<br>Impossibility of measuring the effects of each variable separately
**Hahm & Woo (2019)** [98]	Case report<br><br>*Level4	1	32	Hypoxic - ischemic brain injury	9mo	MMSE-K: 5/30 DoC states not specified	MMSE-K	5mg, single dose<br><br>5mg BID, 3y	MMSE-K ↑ from 5 to 16<br>↑ alertness, verbalisation and spontaneous communication, expressing thoughts, independent eating<br>Near-normal activities (washing hands, voluntary participation in exercises, talking to people)<br>Medication withdrawal led to a return to baseline<br>Drug tolerance started at 1.5y following zolpidem administration with ↓ effect duration and finally with no effect in the 3rd year	/	/

**Kondratyeva et al. (2019)** [99]	Case series<br><br>*Level4	2	18, 26	Non-TBI (cardiac arrest, diffuse ischemic stroke)	3mo, 11y	VS<br>MCS-	CRS-R<br>EEG<br>PET	10mg 2x/w	**P1:**<br>☙↓ wakefulness and consciousness (sedation)<br>☙Substantial positive changes on EEG (growing stability, increasing frequency and amplitude of alpha activity)<br>☙Glucose metabolism recovery in sensorimotor cortex, peristriate cortex, association cortex of occipital, parietal and medial cortex of temporal lobes of both hemispheres<br>**P2:**<br>☙partial convulsive seizure	60min	/
**Bomalaski & Smith (2017)** [100]	Case report<br><br>*Level4	1	24	CNS lymphoma	126d	MCS	MAS<br>MRI<br>DRS<br>GCS<br>CRS-R	10mg, single dose & 10mg, single dose two days after previous administration	☙↑ somnolence 45 minutes post-zolpidem<br>☙Awakened with tactile stimulation appearing to be more interactive as compared to pre-zolpidem<br>☙Functional object use<br>☙↑ motor function<br>☙Following simple commands<br><br>☙Total sedation 1h after the 2nd zolpidem administration,<br>☙↑ arousal, communication, and motor function<br>☙Functional object use<br>☙Answering simple yes/no questions	45min	☙ Possibility of natural recovery<br>☙ Low predictive validity due to the single-patient design
**Kim et al. (2016)** [101]	Case report<br><br>*Level4	1	48	Hypoxic-ischemic brain injury after cardiac arrest	2y	GCS: E2M4V2 (VS, MCS not specified)	GCS<br>EEG<br>[18F]Flumazenil PET<br>SPECT	10mg/d increased to 10 mg TID, 1mo	☙↑ alertness<br>☙Response to commands<br>☙↑ communication, orientation, short-term memory, repetition and comprehension<br>☙↑ swallowing without aspiration<br>☙↑ motor performance,<br>☙Assisted ambulation<br>☙↑ beta activity	30min	/

									ɞ↑ GABA receptor activity in frontoparietotemporal cortices<br>ɞ↑ cerebral perfusion<br>ɞ20-30 mg doses were administered but led to ↑ sleepiness		
**Calabrò et al. (2015)** [102]	Case report<br><br>*Level4	1	52	Cardiac arrest	3y	PVS	CRS-R<br>EEG	Max. 30mg/d; increased by 5mg/w; initial dose not specified	ɞ↑ awareness and wakefulness with 20-30mg<br>ɞ↑ EEG amplitude and voltage	/	/
**Appu & Noetzel (2014)** [103]	Case report<br><br>*Level4	1	16	Anti-NMDA R encephalitis	45d	MCS	WeeFIM	10mg	ɞ↑ neurological function and arousal<br>ɞDischarged earlier than expected (10d later) with high levels of independence	/	/
**Du et al. (2014)** [104]	Prospective cohort study<br><br>*Level2b	165 (4 groups based on the aetiology)<br>*38 patients withdrew	Group 1: 37.5±7.7<br>Group 2: 38.5±7.1<br>Group 3: 38.1±8.3<br>Group 4: 37.9±7.6	Contrecoup contusion<br>Space-occupying compression injury<br>Primary brain stem injury<br>Secondary brain stem injury	>1mo	VS	CRS-R<br>SPECT<br>CSM	10mg/d, 1w	ɞ↑ cerebral state index in the 1st and the 2nd groups after zolpidem administration<br>ɞ↑ blood flow in the 1st and the 2nd group (not in those with brain stem injuries)	30min-1h	/

**Thonnard et al. (2013)** [105]	Prospective open-label study	60	36±14	TBI, Non-TBI	4±5.5 y	VS, MCS	CRS-R	10mg, single dose	☙No response at the group level<br>☙↑ CRS-R total scores and/or ↑ behaviours in 12 patients<br>☙A change of diagnosis in 1/12 patients (not confirmed in a following clinical trial)	/	/
**Rodriguez-Rojas et al. (2013)** [106]	Single-patient placebo-controlled trial<br><br>*Level1	1 patient 1 healthy age and sex-matched control	21	CVA	5y	PVS	H-MRS fMRI	10mg, single dose	☙Patient showing progressive ↑ in N-acetyl-aspartate, glutamate/glutamine, and lactate/lipid after zolpidem with maximum changes of +38%, +42%, and +30%, respectively, as compared to the healthy control with progressive ↓ in N-acetyl-aspartate reaching a maximum of -27%, a slight and steady ↓ in glutamate/glutamine, and no consistent change in lactate/lipid<br>☙Patient showing irregular increasing trend in creatine/phosphocreatine, compared to the control with no consistent changes<br>☙Non-significant slight ↓ (<5%) in creatine/ phosphocreatine, partially reversed towards the end of the session after zolpidem in the patient, as compared to the control with slight ↑ during the first hour, then decreasing<br>☙Patient showing significant stable and reproducible BOLD signal ↑ in inferior and middle frontal cortices, bilateral anterior cingulate cortex, orbitofrontal cortex, left thalamus, right caudate nucleus after zolpidem but not after placebo; with frontal	Immediate effects	☙ Findings limited to a single patient<br>☙ Use of creatine/phosphocreatine leading to confounding results

									BOLD ↑ fading after ~90 minutes<br>꧁Patient showing significant transient activations in middle frontal cortex, precuneus, sensory-motor cortex activation appeared ~1h after zolpidem but not after placebo<br>꧁Healthy control showing robust ↓ in spontaneous activity in orbitofrontal cortex, anterior cingulate, superior parietal cortex, precuneus, cerebellum, with later reductions extended to inferior frontal cortex, temporal lobe, and inferior parietal areas<br>꧁Healthy control having bilateral thalamic inhibition ~45 minutes after zolpidem but not after placebo<br>꧁Healthy control showing maximum BOLD signal ↓ ~92 minutes after zolpidem<br>꧁Significant correlation between BOLD changes and N-acetyl-aspartate/creatine/phosphocreatine as well as BOLD changes and glutamate/glutamine/creatine/phosphocreatine after zolpidem (but not placebo) in the patient		
**Williams et al. (2013)** [107]	Case report<br><br>*Level4	3	45,<br>32,<br>55	TBI<br>TBI/Hypoxia<br>CVA	y,<br>2y,<br>not specified for the 3rd patient	MCS	CRS<br>EEG<br>FDG-PET	10mg, four doses for P1, five doses for P2, single dose for P3	꧁P1: CRS-R ↑ from 10-23<br>꧁P2: ↑ verbal fluency and articulation<br>꧁P3: ↑ spontaneous speech rate<br><br>꧁All 3 patients:<br>꧁↓ 6–10 Hz band power	Immediate	/

									❧↑ widely distributed 15-30 Hz band power		
**Cohen & Duong (2008)** [108]	Case report<br><br>*Level4	1	35	Anoxia (resulted from cardiopulmonary arrest)	8mo	Non-verbal, No command following<br>DoC states not specified	Clinical observation	Initial dose: 5mg/d<br><br>Dose increase: 10+5mg/d, 3w & 10mg BID at 3mo	❧ Marked increase in interaction and participation in activities, lasting ~3h<br>❧ Dose increase resulting in ↑ speech, social interaction with family and staff, walking with assistance, and ↑ willingness to eat<br>❧ One month after zolpidem initiation, improved alertness, discharge to home, recovery of orientation (with cues), verbal social interaction, oral intake, partial independence in self-care, and assisted ambulation up to 300 feet<br>❧ Interruption of zolpidem leading to relapse into lethargy, with resumption and dose increase (10mg BID), restoring positive outcomes<br>❧ At 3mo post-discharge, consistent, dose-dependent arousal within 1h lasting up to 6h, with sustained improvements in speech, independent eating, and assisted to occasional unassisted walking	~1h	/
**Shames et al. (2008)** [109]	Case report<br><br>*Level4	1	50	Anoxia	18mo	MCS	Clinical observation<br>RLAS	10mg/d	❧ Dramatic functional improvement within 30min, lasting ~3h<br>❧ Patient emerging from MCS with resolution of abnormal movements and marked cognitive, communicative, emotional, and motor	30min	/

									recovery, including orientation, meaningful speech, recognition of family, reading and writing abilities, appropriate affect, preserved remote memory despite anterograde amnesia, restoration of voluntary limb movements, feeding and assisted ambulation, and an ↑ RLAS score from 3 to 6<br>Reproducible effects over several consecutive days<br>Non-reproducible & inconsistent positive effects with 5mg		
**Singh et al. (2008)** [110]	Case report<br><br>*Level4	1	44	TBI	4y	GCS: 11 MCS	WHIM WAB Custom therapist-designed tasks (object selection, football test, coloured objects, yes/no cards)	10mg/d, 1w	No improvement on zolpidem, with average test scores not improving on treatment, with significantly better performance on football & yes/no tests off medication<br>Nursing staff observing no change in interaction or behaviour on or off zolpidem	/	/
**Clauss et al. (2000)** [111]	Case report<br><br>*Level4	1	28	TBI	3y	UWS	SPECT EEG	10mg, single dose	↑ wakefulness and communication<br>Generalised cortical activation relative to the cerebellum<br>↑ activation in hypoactive areas	15min	/
**Baclofen**											

**Halbmayer et al. (2022)** [112]	Retrospective study<br><br>*Level3b	26 (paediatric not specified)	28.1±14.2	TBI<br>Cerebral hypoxia<br>SAH<br>IH	Time from event to admission: median = 3.8mo & time from admission to pump implantation: median = 71d	VS<br>MCS	CRS-R<br>MAS	ITB 130–1050 245µg/d, up to 6mo	• ↓ spasticity<br>• ↑ CRS-R<br>• No correlation between CRS-R and MAS scores<br>• No correlation between time to ITB and CRS-R outcome	3mo	• Retrospective single-centre study design<br>• Large range of intervals from the acute event to the time of pump implantation<br>• Small sample size<br>• Heterogeneous and not evenly distributed diagnoses<br>• No fMRI study to differentiate UWS patients from those with LIS<br>• No assessment of comorbidities
**Formisano et al. (2019)** [113]	Case report<br><br>*Level4	1	26	Frontoparietal haemorrhage	14mo	VS	CRS-R<br>NCS-R	ITB 60µg/d	• ↑ behavioural responsiveness<br>• CRS-R score ↑ from 6 to 12<br>• Intentional and purposeful motor responses<br>• NCS-R score changed from 4 to 8	1mo	• Possibility of spontaneous recovery<br>• Anecdotal reporting of recovery of responsiveness and functional communication 14mo after the acute event
**Al-Khodairy et al. (2015)** [114]	Case series<br><br>*Level4	2	26,<br>21	TBI	5mo,<br>9mo	MCS	MAS<br>FIM	ITB<br>**P1:** 38mg/d, 10d; dose escalation to 80mg/d & 650 mg/d<br>**P2:** 24mg/d and 140mg/d at 13mo post-injury, 1mo	**P1:**<br>• Rapid emergence from MCS with marked ↓ of spasticity and vegetative symptoms (hyperhidrosis, hypersalivation, bruxism), accompanied by transient behavioural disturbances (cries, kicks, punches)<br>• Quick improvement in attention, audio-verbal comprehension, and sustained concentration (up to ~20min), enabling command following, object identification, and emergence of verbal and non-verbal communication<br>• Gradual, linear cognitive improvement over months	Immediate	/

									with complete resolution of post-traumatic amnesia at 12mo, discharge to home at 18mo without oral antispastic medications, with assisted communication, but persistent severe cognitive and behavioural deficits (executive, memory, attention, verbal suppression, social inappropriateness)<br>**P2:**<br>ↄ Voluntary upper-limb responses to verbal commands, and within three weeks of continuous ITB, emergence from post-traumatic amnesia with recovery of full consciousness (three-dimensional orientation and coherent speech), despite persistent severe cognitive deficits<br>ↄ Dose escalation leading to sufficiently ↓ spasticity to enable surgical correction of ankle deformities and restoration of walking, allowing discontinuation of ITB therapy 14mo after initiation due to sustained motor and neuropsychological improvement		
**Margetis et al. (2014)** [115]	Prospective open label study<br><br>*Level4	8	31.5±8.1 (range: 20-47)	TBI Anoxia, Acute obstructive hydrocephalus	37.25±33mo, (range: 5–108mo)	VS MCS	CRS-R ECOG performance status scale MAS	ITB 50-800μg/d	ↄ Variable results regarding CRS-R scores<br>ↄ ↑ CRS-R scores of 2/8 patients from 10 to 19 and from 11 to 22 after 3.5 and 2y of pump implantation	Variable	ↄ Single centre study<br>ↄ Small sample size<br>ↄ Findings not generalizable to DoC patients without spasticity

**Hoarau et al. (2012)** [116]	Retrospective cohort study<br><br>*Level3	53	24±1.2 (range: 6-46)	TBI<br>Hypoxia	6.8±1.3 mo, range 0.5–33 mo	PVS<br>MCS	CRS-R<br>Barthel index<br>GOS<br>MAS	ITB<br>**TBI:** 30–1200µg/d (mean: 232.6±42.8µg/d)<br>**Hypoxia:** 100–1200µg/d (mean: 518.1±105.4µg/d)	• ↑ responsiveness in TBI patients compared to patients with hypoxia<br>• ↑ dysautonomia and limb hypertonia in TBI patients<br>• ↑ CRS-R scores in TBI patients with 21/43 patients having a maximal CRS-R score<br>• ↓ disability in ADL in 23/43 TBI patients<br>• Severe disability in ADL in 19/43 TBI patients and all patients with hypoxia<br>• Better recovery based on GOS in TBI patients compared to patients with hypoxia<br>• Complex voluntary movements in 21/43 TBI patients but in any of the patients with hypoxia<br>• ↑ dysautonomia, limb hypertonia, and voluntary motor response in TBI patients<br>• ↑ voluntary motor response and partial ↑ dysautonomia but no changes in limb hypertonia in patients with hypoxia	9.6y (range: 2-16.3y)	/
**Shrestha et al. (2011)** [117]	Case report<br><br>*Level4	1	40	TBI	A few weeks; not precisely speified	GCS: 6	Clinical observation	50µg/day, 2w<br>Increased to 100µg/day, 3w	• ↓ in limb stiffness and clonus with parallel improvement in higher mental functions, including emergence of speech, command-following, purposeful movements, and emotional reactivity<br>• Intensification of physiotherapy with transition to wheelchair sitting and bladder training, accompanied by continued cognitive and	/	/

									functional gains such as short-sentence speech, recognition of surroundings and family, and consistent command-following<br>ꕥ ITB stopped after ~5w due to CSF leakage (epidural administration)<br>ꕥ By discharge at ~2.5mo post-admission, patient fully conscious and oriented, able to speak appropriately and walk with support, with residual mild spasticity, ocular motility deficit, and behavioural disturbances,<br>ꕥ Normal EEG at 2-mo follow-up, and restored independent urination		
**Oyama et al. (2010)** [118]	Case series<br><br>*Level4	2	43, 67	SAH	2mo, 10mo	Reduced level of consciousness indicated with behavioural presentation DoC states not specified	MAS	50µg/d	**P1:**<br>ꕥ Significant ↓ of MAS score after a single dose (50µg/d)<br>ꕥ ITB implantation leading to improvement with marked spasticity ↓ by 3mo, further MAS score ↓ by 8mo, recovery of feeding, grooming, and writing abilities within 1-3mo, ability to stand with assistance by 4mo, emergence of speech by 8mo<br>**P2:**<br>ꕥ Significant ↓ of MAS score after a single dose (100µg/d)<br>ꕥ ITB implantation leading to recovery with command-following within days, swallowing and orientation by 2w, independent eating by 3-4w, marked spasticity ↓ by 3mo, ↓ MAS score at 6mo, ambulation with cane and	Immediately/days	/

									stair use by 2-3mo, fluent speech by 6mo		
**Sarà et al. (2009)** [119]	Case series<br><br>*Level4	5	34.6	SAH<br>ICH<br>TBI<br>Hypoxia	9.2±2.6 mo (range: 6-10mo)	PVS	CRS-R<br>DRS<br>MAS	ITB 100mcg/d Increased progressively over the next 30d; range: 200-325 mcg/d, 6-mo	**P1:** CRS-R ↑ from 7 to 23 during the first four mo (subsequently ↓ to 9)<br>**P2:** CRS-R ↑ from 7 to 23; stable ↑ motion and communication, simple motor abilities, object manipulation, independent eating, moving wheelchair, and talking<br>**P3:** CRS-R ↑ from 6 to 21; command following and object manipulation<br>**P4 & P5:** CRS-R ↑ from 6 to 12 and 5 to 11, respectively; ↑ alertness, more sustained eye opening, spontaneous unpurposeful vocalisation<br>**Overall:**<br>❧↓ DRS scores in P2 and P3<br>❧↓ MAS scores in all patients (mean for upper limbs and lower limbs: -0.9±0.2 and -1.2±0.2, respectively) associated with ↑ CRS-R scores	2w	/
**Taira (2009)** [120]	Case series<br><br>*Level4	2	8,<br>18	TBI	2.5mo,<br>1.5mo	PVS	P1: GCS<br>P2: GCS	ITB<br>**P1:**<br>50, 75, and 100μg/d, 23d<br>**P2:**<br>50 and 100μg/d, 23d	**P1:** opening eyes, verbalisation, command following, sitting up, walking with leg braces, playing in the high school basketball team<br>**P2:** ↓ spasticity, command following, taking food orally, discharged to rehabilitation centre, ambulation, independent living	P1: 3d<br>P2: 5d	❧ Possibility of spontaneous natural recovery
**Midazolam**											

**Carboncini et al. (2014)** [121]	Case report<br><br>*Level4	1	43	TBI	>2y (not specified, inferred from the text)	MCS+	Wada test<br>Adapted MMSE<br>EEG	5mg in 100 cc of saline solution in 8min	• Emergence from MCS<br>• Understanding verbal commands - Following any type of behavioural or verbal commands<br>• More cooperation<br>• Understandable speech production<br>• ↓ power in 1-12 Hz band with the highest ↓ at 7 Hz (as compared to pre-midazolam)<br>• ↑ power in 12-30 Hz band with the highest difference at 15 Hz (as compared to pre-midazolam)<br>• A more balanced distribution of power across high and low, leading to a profile/spectral pattern normalisation<br>• ↓ activity of lower frequencies (delta, theta, and alpha bands) on anterior cortical regions and simultaneously ↑ activity of higher frequencies (beta band) on posterior cortical regions<br>• Midazolam-induced removal of the low frequency (theta2 band) intrinsic connectivity while fostering it in the high frequencies (beta1 band) in task-positive and linguistic networks on intra- or inter-hemispheric level	>5min	/
**Other pharmacological agents**											
**Tricyclic antidepressants**											
**Reinhard (1996)** [122]	Case series<br><br>*Level4	3	27,<br>23,<br>26	RTA	6mo,<br>2mo,<br>19mo	VS	**P1:** GCS;<br>**P2:** RLAS;<br>**P3:** RLAS, CT, EEG	**P1: Amitriptyline** 10 and 50mg/d<br>**P2:**	**P1:** ↑ arousal, verbalisation and response to yes/no questions, following simple commands, verbalizing needs, adequate responsiveness and participation, starting	**P1:** Within "several days".<br>**P2:** "rapid"	• Not an RCT<br>• Small sample size<br>• Different assessments in each patient and no detailed report of the results<br>• Not controlling for confounding variables

								**Desipramine** 50mg/d **P3: Desipramine** 75mg/d	ambulation training, discharged home, assisted ambulation, feeding himself, performing ADL, verbalizing basic needs<br>**P2:** ↑ arousal, following simple commands, starting functional rehabilitation, discharged home, assisted ambulation in short distances, performing ADL with supervision, being alert, oriented and able to learn despite memory issues<br>**P3:** Verbalizing single words, consistent use of two-word phrases, verbalizing care needs, discharged to a nursing home, following simple commands, performing self-care activities with assistance<br>**Overall:**<br>ᴄᴈ Discontinuation of the drug administration led to functional deterioration in the first two patients but not in the last<br>ᴄᴈ Reinitiation of the drug administration led to the same functional improvements	improvement. **P3:** 4d	ᴄᴈ Not controlling for spontaneous recovery
**Anticonvulsants**											
**Hérault et al. (2024)** [123]	Case series<br><br>*Level4	4 (1 paediatric <18y)	17, 32, 18, 20	TBI	299d, 147d, 280d, 111d	CRS-R: **P1:** 5-7 (UWS-level behaviours) **P2:** 8-9 (MCS-level behaviours)	CRS-R Clinical observation	**Lamotrigine** 25-100mg/d, >4w	**P1:**<br>ᴄᴈ Notable improvements in eye-opening, left leg movements on command, communication with a yes/no code ~10d after lamotrigine administration, administered for seizure management<br>ᴄᴈ Stronger, more pertinent motor responses, including goal-directed movements	Within days	/

						**P3:** 7-9 (MCS-level behaviours)<br>**P4:** 2 (UWS-level behaviours)			within weeks after lamotrigine<br>**P2:**<br>• Increased alertness, more consistent visual pursuit, goal-directed movements of the left arm, engagement in speech therapy within days after lamotrigine administration to address mood disorders<br>• Improved verbalisation, functional verbal communication and object use after 2w of administration<br>**P3:**<br>• Increased alertness, more facial expressions, reduced opposition during rehabilitation, & increased social engagement within days of lamotrigine administration<br>• Increased CRS-R score with functional communication using head movements, some yes/no responses, occasional verbal responses, & functional use of objects within weeks of administration<br>• Lamotrigine discontinuation after 2mo leading to no regression in vigilance or level of consciousness, no epileptic events, but increased aggression, disinhibition, and worsened frontal syndrome<br>• Clear improvement in frontal syndrome after re-administration of lamotrigine on d400 after TBI<br>**P4:**		

									☙ Improved vigilance, more consistent visual pursuit, spontaneous movements of the left arm, reproducible responses to simple commands within days of lamotrigine administration<br>☙ Spontaneous auditory orientation, stronger and more varied motor responses on command, & improved communication after weeks of lamotrigine administration<br>☙ CRS-R score increased to 21 ~2mo after lamotrigine initiation		
**Ng et al. (2021)** [124]	Case report<br><br>*Level4	1	29	ABI due to RTA	5mo	MCS	GCS<br>CT<br>RLAS	**Lamotrigine** 25mg/d with slow up-titration to 50mg/d over 2w and up-titration to 50mg BID on w4, continuing gradual increase to 200mg BID over 13w in total	☙Producing a short sentence<br>☙Verbalizing basic needs<br>☙Initiating a question<br>☙Being able to participate in a fulsome rehabilitation program<br>☙↑ visual tracking and right arm motor function<br>☙Responding to his name<br>☙↑ orientation<br>☙↑ RLAS score (Level VI) with consistent simple command following<br>☙Learning pre-injury familiar tasks<br>☙↑ awareness of self and environment<br>☙Discharged to complex continuing care<br>☙consistent functional improvements along with ↑ insight in spite of major neurocognitive disorder diagnosed based on DSM-5<br>☙↓ dose leading to drowsiness and less responsiveness	2w	☙ Changing medications for the spasticity management<br>☙ Findings not generalizable

**Bagnato et al. (2013)** [125]	Retrospective study<br><br>*Level3	103<br><br>Anticonvulsant: 54	45.9±19.1	Vascular injury<br>Hypoxia<br>TBI	54.5±25.9 d	VS<br>MCS	LOCFAS	/	• No responder	/	• Variability in the choice of anticonvulsants (both new and old generations)<br>• Short temporal window of evaluation
**Showalter & Kimmel (2000)** [126]	Case series<br><br>*Level4	13	49.31±13.76	TBI<br>SAH<br>Meningioma resection<br>Encephalitis	50.91±23.02 d	RLAS score of 2.6 for TBI and 2 or 3 for nonTBI patients<br><br>DoC states not specifiefd	RLAS Discharge	**Lamotrigine**: 15-400 mg/d<br><br>Varied duration of administration	• Greater-than-expected cognitive improvement in 9 patients<br>• Expected modest cognitive improvement in 4 patients<br>• 10 patients (75%) discharged to community settings after a mean rehabilitation stay of 72 d<br>• 3 patients discharged to skilled nursing facilities after a mean stay of 117 d	/	/
**Combination of the drugs**											
**Leclerc et al. (2021)** [53]	Retrospective cohort study	87<br><br>• adine: 62<br>• nil: 15<br>• 24	See above	See above	See above	See above	See above	See above	See above	See above	See above
**Bender Pape et al. (2020)** [55]	Pilot alternate-treatment, within-subject, baseline-controlled trial	4 (1 paediatric)	See above	See above	See above	See above	See above	See above	See above	See above	See above
**Lee et al. (2020)** [57]	Retrospective single-centre case-control study<br><br>*Level4	42 in dual regimen receiving amantadine and cerebrolysin	54.8±17.2 (total sample: 60.2±17.1)	Stroke (ischemic/hemorrhagic), TBI, HBI, Unspecified	24.9±25.6w	VS<br>MCS-<br>MCS+	CRS-R	**Amantadine:** 100mg BID in w1, 150mg BID in w2,	• ↑ mean change in CRS-R total score (compared to the group receiving amantadine only)<br>• DoC category change in all patients in a VS	Approx. 1mo	• Potential bias in allocating patients to groups based on one physician's decision<br>• Heterogeneity in demographic and clinical characteristics of patients leading to non-comparability of the dual vs. single regimen group

		(total sample: 84 with 42 in the single regimen receiving amantadine only)		(metabolic encephalopathy, leukoencephalopathy)				200mg BID in w3, 100mg BID in w4)<br>**Cerebrolysin**: 2.125mg/ 10ml BID (equal to 20ml/d)	❧↑ mean change in CRS-R visual function score in the dual regimen group<br>❧↑ change in CRS-R oromotor/verbal function score		❧ Results not generalizable to all patients with DoC<br>❧ Uneven distribution of aetiologies in dual vs. single regimen<br>❧ Potential biases in the results due to lower mean age in the dual regimen group<br>❧ Cerebrolysin administered in a lower dose compared to previous studies<br>❧ No follow-up assessment after discontinuation of the drug administration

[1]In years, mean ± Standard Deviation (SD)/median/range; [2]Mean ± SD/median, range [3]Before intervention; [4]Max./range; [5]Time interval between treatment administration and first signs of improvement; **Aetiology:** ABI: Acquired Brain Injury; CAMB: Community-Acquired Bacterial Meningitis; DAI: Diffuse Axonal Injury; HBI: Hypoxic Brain Injury; HI: Head Injury; ICH: Intracranial Haemorrhage; IH: Intracerebral haemorrhage; IS: Ischemic Stroke; IVH: ; NTAR: Non traumatic Aneurysm Rupture; PIHV: Postoperative Intracranial Hypotension-associated Venous congestion; RTA: Road Traffic Incident; SAH: subarachnoid haemorrhage; SDH: Subdural Hematoma; SE: Status Epilepticus; TBI: Traumatic Brain Injury; **Diagnosis:** CMD: Cognitive Motor Dissociation**;** (e)MCS: (emerging from) Minimally Conscious State; UWS: Unresponsive Wakefulness Syndrome; (P)VS: (Persistent) Vegetative State; **Behavioural outcome measures:** ABS: Agitated Behaviour Scale; BI: Barthel Index; BRISC: Barry Rehabilitation Inpatient Screening of Cognition; BMC: British Medical Council; CNCS: Coma-Near Coma Scale; CRS-R: Coma Recovery Scale-Revised; ECOG: Eastern Cooperative Oncology Group; GCS: Glasgow Coma Scale; GOS: Glasgow Outcome Scale; DRS: Disability Rating Scale; FES: Falls Efficacy Scale; FIM™: Functional Independence Measure; FND: Focal Neurological Deficits; FOUR: Full Outline of Unresponsiveness; GOAT: Galveston Orientation and Amnesia Test; IDT: Interdisciplinary Team; LOCFAS: Levels of Cognitive Functioning Assessment Scale; MAS: Modified Ashworth Scale; MoCA: Montreal Cognitive Assessment; mRS: modified Rankin Scale; NIHSS: National Institutes of Health Stroke Scale; O-Log: Orientation Log; RASS: Richmond Agitation-Sedation Scale; RLAS: Rancho Los Amigos Scale; SAPS: Simplified Acute Physiology Score; WAB: Western Aphasia Battery; WeeFIM: Functional Independence Measurement for Children score; SAS: Sedation-Agitation Scale; WNSSP: Western NeuroSensory Stimulation Profile; WHIM: Wessex Head Injury Matrix; **Neuroimaging outcome measures:** CPC: Cerebral Performance Category; CSM: Cerebral State Monitor; DTI: Diffusion Tensor Imaging; ECG: Electrocardiography; (hd)EEG: high-density Electroencephalography; fNIRS: functional Near-Infrared Spectroscopy; HRV: Heart Rate Variability; MEG: Magnetoencephalography; (f)MRI: (functional) Magnetic Resonance Imaging; MRS: Magnetic Resonance Spectroscopy; PET: Positron Emission Tomography; SPECT: Single-Photon Emission Computed Tomography; **Other:** BID: twice a day; CO: Carbon Monoxide; CoE: Class of Evidence; d: day; GPe: external Globus Pallidus; GPi: internal Globus Pallidus; h: hour; ICU: Intensive Care Unit; IQR: Interquartile Range; min: minutes; mo: month; NMDAR: N-methyl D-aspartate Receptor; P: Patient; QD: four times a day; rTMS: repetitive Transcranial Magnetic Stimulation; SOC: Standard of Care; TID: thrice a day; w: week; y: year.

Supplementary Table 3. Characteristics of RCTs included in the Multiple Correspondence Analysis (MCA)

Study ID	Treatment	Study Design	Blinding	Sex Distribution	Aetiology	Outcome Measures	Age Category	Sample Size	Treatment Administration	RoB
**Amantadine**										
Coppler2024[25]	Amantadine	Parallel	Double-Blind	Male-dominant	NonTBI	Neurobehavioural & Clinical	Mixed Age	7	Multiple administration	Some Concerns
Gatto2024[26]	Amantadine	Parallel	Double-Blind	Female-dominant	NonTBI	Neurobehavioural	Mixed Age	20	Multiple administration	Some Concerns
Shimia2023[27]	Amantadine	Parallel	Triple-Blind	Male-dominant	TBI	Neurobehavioural	Young Adult	29	Multiple administration	Low
Shafiee2022A[28]	Amantadine	Parallel	Single-Blind	Male-dominant	TBI	Neurobehavioural	Mixed Age	22	Multiple administration	Some Concerns
Abbasivash2019[29]	Amantadine	Parallel	Double-Blind	Male-dominant	TBI	Neurobehavioural	Mixed Age	33	Multiple administration	Low
Akcil2018[30]	Amantadine	Parallel	No Blinding	Male-dominant	NonTBI	Neurobehavioural	Mixed Age	5	Multiple administration	Some Concerns
Ghalaenovi2018[31]	Amantadine	Parallel	Double-Blind	Male-dominant	TBI	Neurobehavioural	Mixed Age	19	Multiple administration	High
Giacino2012[32]	Amantadine	Parallel	Double-Blind	Male-dominant	TBI	Neurobehavioural	Mixed Age	87	Multiple administration	Low
Patrick2006[34]	Amantadine	Parallel	Double-Blind	Female-dominant	TBI	Neurobehavioural	Child/Adolescent	6	Multiple administration	Low
McMahon2009[33]	Amantadine	Crossover	Double-Blind	Male-dominant	TBI	Neurobehavioural	Child/Adolescent	7	Multiple administration	High
Meythaler2002[35]	Amantadine	Crossover	Double-Blind	Male-dominant	TBI	Neurobehavioural	Mixed Age	35	Multiple administration	Some Concerns
**Apomorphine**										
Othman2025A[36]	Apomorphine	Crossover	Double-Blind	Male-dominant	Mixed Aetiology	Neurobehavioural & Clinical	Mixed Age	50	Single administration	High
**Modafinil**										
TalebiKiasari(2024)[38]	Modafinil	Parallel	Double-Blind	Male-dominant	NonTBI	Neurobehavioural & Clinical	Mixed Age	9	Multiple administration	Low
Zand2024[39]	Modafinil	Parallel	Double-Blind	Male-dominant	TBI	Neurobehavioural & Clinical	Mixed Age	41	Multiple administration	Low
**Zolpidem**										
RahatDahmardeh2024[40]	Zolpidem	Parallel	Double-Blind	Male-dominant	Mixed Aetiology	Neurobehavioural & Clinical	Mixed Age	40	Multiple administration	High
Shafiee2022Z[28]	Zolpidem	Parallel	Single-Blind	Male-dominant	TBI	Neurobehavioural	Mixed Age	22	Multiple administration	Some concerns
Chatelle2014[41]	Zolpidem	Crossover	Double-Blind	Female-dominant	NonTBI	Neurobehavioural & Imaging	Mixed Age	3	Single administration	Low

Machado2014[42]	Zolpidem	Crossover	Double-Blind	Male-dominant	Mixed Aetiology	Neurobehavioural & Imaging	Mixed Age	8	Single administration	Low
Whyte2014[43]	Zolpidem	Crossover	Double-Blind	Male-dominant	Mixed Aetiology	Neurobehavioural	Mixed Age	84	Single administration	Low
Whyte2009[44]	Zolpidem	Crossover	Double-Blind	Male-dominant	Mixed Aetiology	Neurobehavioural	Mixed Age	15	Single administration	Low
BrefelCourbon2007[45]	Zolpidem	Crossover	Double-Blind	Female-dominant	NonTBI	Neurobehavioural & Imaging	Middle-Aged	1	Single administration	Some Concerns
**Methylphenidate**										
Othman2025M[36]	Methylphenidate	Crossover	Double-Blind	Male-dominant	Mixed Aetiology	Neurobehavioural & Clinical	Mixed Age	50	Single administration	High
Moein2006[37]	Methylphenidate	Parallel	Double-Blind	Male-dominant	TBI	Clinical only	Mixed Age	41	Multiple administration	High
**Simvastatin**										
Shafiee2021[46]	Simvastatin	Parallel	Double-Blind	Male-dominant	TBI	Neurobehavioural	Mixed Age	49	Multiple administration	High

TBI: Traumatic Brain Injury; RoB: Risk of Bias; Sample Size indicates the number of patients included (excluding the control group).

Supplementary Table 4. Distribution of variables in included RCTs (n and %)

Category	Variable	n	%	Category	Variable	n	%
**Treatment**	Amantadine	11	45.83	**Outcome Measures**	Clinical only	1	4.16
	Apomorphine	1	4.16		Neurobehavioural	14	58.33
	Methylphenidate	2	8.33		Neurobehavioural & Clinical	6	25
	Modafinil	2	8.33		Neurobehavioural & Imaging	3	12.5
	Simvastatin	1	4.16	**Age Group**	Child/Adolescent	2	8.33
	Zolpidem	7	29.16		Middle-Aged	1	4.16
**Study Design**	Crossover	9	37.5		Mixed Age	20	83.33
	Parallel	15	62.5		Young Adult	1	4.16
**Blinding**	Double-Blind	20	83.33	**Treatment Administration**	Multiple administration	17	70.83
	No Blinding	1	4.16		Single administration	7	29.16
	Single-Blind	2	8.33	**RoB**	High	7	29.16
	Triple-Blind	1	4.16		Low	10	41.66
**Sex Distribution**	Female-dominant	4	16.66		Some Concerns	7	29.16
	Male-dominant	20	83.33	**Sample Size**	Small	11	45.83
**Aetiology**	Mixed Aetiology	6	25		Medium	9	37.5
	NonTBI	6	25		Large	4	16.66
	TBI	12	50				

Supplementary Table 5. Dimensional structure of the MCA: eigenvalues, variance, and cumulative inertia

Dimension	Eigenvalue	Variance (%)	Cumulative Variance (%)	Dimension	Eigenvalue	Variance (%)	Cumulative Variance (%)
**Dim 1**	0.4335769583280576	18.851172101219895	18.851172101219895	**Dim 13**	0.048927287516217936	2.1272733702703452	93.58804532266844
**Dim 2**	0.3387497422578166	14.728249663383334	33.579421764603225	**Dim 14**	0.04098119821701799	1.7817912268268694	95.3698365494953
**Dim 3**	0.22244786050733817	9.671646109014704	43.25106787361793	**Dim 15**	0.03067525477174344	1.3337067292062366	96.70354327870155
**Dim 4**	0.1884154890887985	8.191977786469499	51.44304566008743	**Dim 16**	0.0283417085651442	1.2322481984845306	97.93579147718607
**Dim 5**	0.17613849783157717	7.65819555789466	59.101241217982086	**Dim 17**	0.017377748228805296	0.7555542708176216	98.6913457480037
**Dim 6**	0.1739725042016937	7.564021921812769	66.66526313979486	**Dim 18**	0.015892921448716898	0.6909965847268217	99.38234233273052
**Dim 7**	0.15033094331036748	6.536127970015977	73.20139110981084	**Dim 19**	0.006920125914109533	0.30087503974389274	99.6832173724744
**Dim 8**	0.11873700339717026	5.16247840857262	78.36386951838347	**Dim 20**	0.004738835527125795	0.20603632726633891	99.88925369974073
**Dim 9**	0.10970765447834026	4.769898020797402	83.13376753918087	**Dim 21**	0.002054541435845024	0.08932788851500105	99.97858158825574
**Dim 10**	0.07282796182739312	3.166433122930136	86.300200662111	**Dim 22**	4.926234701187234e-4	0.021418411744292324	100.00000000000003
**Dim 11**	0.060031125045623204	2.610048915027096	88.91024957713809	**Dim 23**	2.524666789723868e-32	1.0976812129234208e-30	100.00000000000003
**Dim 12**	0.05866201463097998	2.5505223752599995	91.46077195239809				

Eigenvalues quantify the amount of corrected inertia (variance) captured by each dimension. Each eigenvalue corresponds to a latent dimension extracted by the analysis and reflects the relative importance of that dimension in structuring associations among categories. A larger eigenvalue indicates that the corresponding dimension explains a greater share of the overall variability in the dataset, whereas smaller eigenvalues indicate dimensions that capture progressively less structured information.

Supplementary Table 6. Category contributions (%) to the MCA structure

Category	Blinding	Aetiology	Outcome Measures	Age Group	Quality (RoB)	Sample Size	Sex Distribution	Study Design	Treatment	Treatment Administration
**Dim 1**	-3.00649639363301	0.7661043170859013	0.8512281765504254	6.18959107602679e-4	-0.06022337572154565	0.3721034079756688	0.6109895897233377	0.4073912802155417	0.6853907963067598	0.8426520252490877
**Dim 2**	1.6169570866022842	0.08430874072344695	-0.8856440833459396	2.681297718102202	-0.03881672258965174	-0.8692439774516584	1.158338773233466	-0.05445952418161869	-3.918119079080391	-0.12305691993739987
**Dim 3**	2.2048256513189957	-0.13097003781647962	-1.1402703410829345	3.765597315144743	-0.28100317377105904	0.1670743381159523	-0.025937849925327553	0.1097726260576436	-1.3751000946684862	0.21410093646762177
**Dim 4**	-0.26154152032632555	-0.32707744873684297	1.8726315523742219	1.2991948915861866	0.20171018594715132	-0.08747623126213094	0.287770053518438	0.10268254625003534	-0.5699862237773085	0.09974166860950649
**Dim 5**	2.18957313680104	0.1685631551951173	0.6385022242175815	-1.0433877700311076	0.03739229525533305	-0.04626891888845311	-0.434467286337911	-0.07248385986398845	0.26110396088263	0.07578811372611187
**Dim 6**	-1.428697821816004	-0.03413105101881145	3.199369368466745	1.8976724154159663	-0.10548799697730515	-0.40385487312339485	0.413477879863991	-0.0856763780827779	1.1262693642231258	-0.06704170630167078
**Dim 7**	3.4484884061487717	0.28775922000601023	0.8187706629774419	1.5749804479083667	0.20740768136220056	0.24292591133974872	-0.01216925261234406	0.003862145738358967	-0.16312303107354836	0.0388807225131325
**Dim 8**	-0.4712533811554599	-0.009372986220665387	-0.9730718584534267	1.775068686687576	0.1822919871766212	-0.046987494162004165	0.2119374210261542	-0.020463969184378455	3.7071721388884096	-0.03314965429518738
**Dim 9**	-0.9931833450924958	-0.17653898763082962	0.05916013981652224	1.9502687054276169	0.08312825786052058	0.18015990194533943	0.40062947085311096	0.016963204636188906	-0.2692804821752265	-0.04873606424677214
**Dim 10**	1.3866436313124542	0.12041381096852138	0.08592764225989136	1.0628953541647368	0.13523944004895158	-0.6677459919439107	-0.40475553740486403	0.049395383968083906	-0.6945003829492458	-0.053899472207695726
**Dim 11**	0.7363359386045781	-0.03845081437232192	1.437918033494308	-1.2771033384148136	-0.08595123902732171	-0.20038216498627187	0.2584155764130992	-0.06193409385133057	1.5745771601649334	0.021903403305207257
**Dim 12**	1.2512121330428108	-0.2690187609605903	0.27919750063419174	1.6601769904294457	-0.047060664811160356	0.11258563551689836	-0.43298517099073075	0.11147097648839688	1.0213196247938645	0.04767026763126097
**Dim 13**	2.088826134838985	0.07406310560783169	-0.43103063373915146	0.8063918492799282	-0.03675270762922132	0.2379840097379101	0.3970732045787626	-0.07519789251799447	1.437058257275476	0.07997936517701829
**Dim 14**	0.1478439357061462	-0.05210182942640923	0.046155863614926906	-1.0909577630819896	0.026679383662176204	-0.1413229348188336	-0.16608190509376425	0.15531073209710552	1.143593369965747	0.10502574405815261
**Dim 15**	-0.17137821328300473	0.06631486011654406	-0.2296130257018803	-0.2737323151859675	-4.567360809524801e-4	-0.08373724379782127	0.37140132351655375	0.09450784472591617	0.3556359411920217	0.004888358062483742
**Dim 16**	-0.41080330714436447	0.11664818803086796	0.0518218623440887	0.5267820301875799	0.013400202670390715	-0.08833678918683086	-0.11639626649779598	0.026272415829521903	0.676763921632331	0.02985951699942128
**Dim 17**	-0.1941827511391305	0.05806668485829257	0.7645888038647818	0.45601050509243607	-0.04070547341941877	0.12975781291046445	-0.27080787139422574	-0.00936574121633567	0.01409513119161486	-0.04812577503157345

**Dim 18**	-0.0504832080457604	0.01327845145456476	-0.06956843199945353	0.2734452612644277	-0.047270710830307365	-0.2149385109156689	-0.022077927792589314	-0.056642630836803806	0.538624470336357	0.09163456999861559
**Dim 19**	0.0068439498293065976	0.0038662593318374452	0.29976062643997325	-0.07700430340536282	0.04901133547850746	0.07882685455386729	0.06894824599326874	-0.010249455100768034	0.1282427267102943	-0.06082333158494928
**Dim 20**	0.04765951406924663	0.1385184801325233	0.11789190193656078	0.1334098003925766	-0.009240146240508507	0.020941088986925864	-0.00867582158530408	0.007923307408226436	-0.1542487423338803	-0.013928121360497286
**Dim 21**	0.12986372749210956	-0.02978898513451186	0.20782493041844227	-0.04374676597628696	0.008276868403915812	0.010615870229037322	0.04412586102403805	0.0018851340544733942	-0.3187186937199704	0.06531344606622122
**Dim 22**	0.08759158696934222	-0.0038071139147912877	0.03621379346695029	-0.0063861796777736344	-0.0096515094833062933	-0.0041503147104577	0.027268684441613088	0.01601632029532771	0.0011114150252452253	-0.03530692338014681
**Dim 23**	1.0879284929233165e-31	1.1954259392280767e-31	8.343333153071778e-32	1.0668089428283892e-31	8.638954385228449e-32	-1.1856675992016449e-32	4.423259760865771e-32	1.4959235348521449e-33	2.2254479677614456e-31	2.571653586831067e-32

Values represent the summed contributions of each category to each MCA dimension, indicating the relative importance of each variable in defining the corresponding dimension. Categories contributing significantly to both Dimensions 1 and 2 have the highest discriminatory power.

Supplementary Table 7. MCA variable coordinates

Variable	Level	Dim 1	Dim 2	Dim 3	Dim 4	Dim 5	Dim 6	Dim 7	Dim 8
**Pharmacological Treatment**	**Amantadine**	-0.6335734451871757	0.4129607536574133	0.07994908781607307	-0.06565586815959568	-0.43826975507780835	-0.31428736121479695	0.3089102211185886	-0.12528432342814463
	**Apomorphine**	1.419183214092111	-2.073075635121444	0.03208434778446505	-0.8367427371355716	-0.36611130116676494	-1.3480075992886982	1.317780428358757	1.7404246724274277
	**Methylphenidate**	0.1946802087243169	-1.7175042252523884	-0.814498112332845	1.2145788372087893	-0.006003611620632999	1.5024463964604742	1.1213824471174434	-0.9498002846747379
	**Modafinil**	-0.3158009622348348	-0.14734856385539433	-0.5462745492817124	-2.2670451825122933	0.6225287360052074	1.3706760231943034	-1.130338286162726	-0.04732097391669067
	**Simvastatin**	-0.9410089539335772	-0.6687121304442517	-0.44909118167485174	1.0050435183752342	-0.13521818484251755	0.05819493369349984	-1.2946872846611017	3.3320209894831465
	**Zolpidem**	0.9619107348459196	0.2755607219356744	0.32273031302038474	0.3798352084461284	0.5841780775851464	-0.14275302862165645	-0.48617055684450944	-0.24286794100259124
**Study Design**	**Crossover**	1.0184782005388542	-0.13614881045404661	0.27443156514410894	0.25670636562508864	-0.18120964965997133	-0.21419094520694515	0.009655364345897631	-0.05115992296094563
	**Parallel**	-0.6110869203233125	0.08168928627242793	-0.16465893908646534	-0.1540238193750533	0.10872578979598288	0.12851456712416726	-0.0057932186075386645	0.030695953776567177
**Blinding**	**Double-Blind**	0.21189630007162452	-0.10142694527825648	-0.09810774044269217	-0.038104415614794225	-0.2019806380318473	0.1502785562407141	-0.14754697727688282	0.05059328894449961
	**No Blinding**	-0.5416455365937822	1.2722893424009554	-1.2505470393474878	-0.9110166604593691	0.14551149760228516	-1.1631538786305575	2.5532299022550142	-0.5748683166258552
	**Single-Blind**	-1.0195333077278568	0.3101548736845878	-0.3407785829078458	0.9855254170074162	1.6480589858040569	-1.4265947467575664	-0.6450958378879985	-0.4900191087900295
	**Triple-Blind**	-1.6572138493829955	0.13593981579499767	3.8942590140170217	-0.2979458612595784	0.5979832914265452	1.0107722473314056	1.687901319058639	0.5430407553159252
**Sex Distribution**	**Female-dominant**	0.763736987154172	1.4479234665418326	-0.032422312406659534	0.3597125668980475	-0.5430841079223887	0.5168473498299888	-0.01521156576543002	0.2649217762826927
	**Male-dominant**	-0.15274739743083438	-0.2895846933083666	0.006484462481331981	-0.07194251337960952	0.10861682158447773	-0.1033694699659978	0.0030423131530859593	-0.0529843552565385
**Aetiology**	**Mixed Aetiology**	1.0770413723166892	-0.9695851128129628	0.31208381388830575	-0.09653661758574954	0.11919543537454197	-0.42880214355375984	-8.125900382243246e-4	-0.21290563579200414
	**NonTBI**	0.4551672618551138	1.1382025942598566	-0.5740238895212652	-0.5576182798879367	0.21793087501569267	0.36054004151613855	0.5763310300502449	0.19415966335067275
	**TBI**	-0.7661043170859015	-0.08430874072344686	0.13097003781647978	0.3270774487368432	-0.16856315519511736	0.03413105101880984	-0.28775922000601034	0.009372986220666001
**Outcome Measures**	**Clinical only**	-0.7474039591458191	-1.4668986250985268	-1.2805048913954986	2.1771780436285066	0.14965831849222008	2.714449529627062	1.0556282155370589	-1.3743246765508452
	**Neurobehavioural**	-0.4511414440598839	0.18816397347138922	0.20355018718477083	0.1782303639274482	-0.2229043019348274	-0.4624516461241458	-0.06155351490207152	-0.06797213734737179
	**Neurobehavioural & Clinical**	0.30468781223860153	-0.7822229027815051	-0.4597502728582236	-1.0746908576891947	0.27858576187159867	0.3059196870731632	0.11067770202152624	0.30608657769322584

	**Neurobehavioural & Imaging**	1.7450857675175269	1.1753134710627031	0.3964346359860168	0.5919140025074616	0.43316244578859014	0.6414517978906662	-0.2859817396790717	0.1631383777515646
**Age Category**	**Child/Adolescent**	-0.3843936137812493	0.5455228117258094	0.13740818497110097	0.3446695300365172	-2.6671075725738236	-0.06881498239410601	-0.5789464763348449	-0.251902042410332
	**Middle-Aged**	2.0220277562363926	2.169667750046027	-0.060648541732019355	1.3389904028946529	0.8304472825580161	1.0519708048482361	0.5184484457937055	1.564096639270257
	**Mixed Age**	0.02019866603545503	-0.1698326594646323	-0.20542134211136023	-0.08651918008540545	0.19528922855815434	-0.09625565436957163	-0.05242284060913267	-0.08016666548827611
	**Young Adult**	-1.6572138493829955	0.13593981579499767	3.8942590140170217	-0.29794586125957806	0.5979832914265455	1.0107722473314078	1.6879013190586387	0.5430407553159269
**Treatment Administration**	**Multiple administration**	-0.5898564176743615	0.08613984395618005	-0.14987065552733528	-0.06981916802665446	-0.053051679608278265	0.04692919441116953	-0.027216505759192655	0.023204758006631118
	**Single administration**	1.4325084429234491	-0.2091967638935799	0.36397159199495704	0.16956083663616095	0.12883979333439013	-0.1139709007128403	0.06609722827232516	-0.0563544123018185
**RoB**	**High**	0.00673345497648787	-1.010371920814233	-0.3849281324463562	0.4424091985168853	-0.4790923332168947	0.18395535050400683	0.2116651222317777	0.5445531595750963
	**Low**	0.14052121001694043	0.09057235270918791	0.6556740721324708	-0.4706571005433533	-0.08724868892911085	0.24613865961371217	-0.48395125651180204	-0.4253479700787832
	**Some Concerns**	-0.20747804071497394	0.8809828455153933	-0.5517491134571736	0.2299580879736193	0.6037333174013386	-0.5355820070950241	0.4796938156422249	0.0630867976803081
**Sample Size**	**Small**	0.36371303161074836	0.7088655813962882	-0.1755278920974329	-0.20808945901240194	-0.40928002728812196	0.11554828397590747	0.06407339460110979	-0.09245278730929098
	**Medium**	-0.8068809706355822	-0.2970166319934766	0.11207957844364395	0.3613062276270676	0.6100071733141329	0.16131630093244573	-0.28404348151335274	0.1670238975626105
	**Large**	0.8152713470005026	-1.28109292685447	0.23052265176974124	-0.2406929998767966	-0.24699606491446405	-0.680719458031748	0.4628959982519917	-0.12155860441532368
**Pharmacological Treatment**	**Amantadine**	0.11795238152890414	-0.1371764901230562	-0.1279736024710429	-0.0866961452450564	-0.29957119606366067	0.04791329269277605	-0.05158202097184487	-0.15501930075060988
	**Apomorphine**	1.6983923035318955	-0.1311516436703999	2.4255675981484104	0.455469608178039	-0.3150589258269935	0.003924038039856571	0.07153190806006031	0.23115246769326356
	**Methylphenidate**	0.04899850322728123	-0.2638158028254402	-0.6437362289055595	-0.10433743246575812	0.6814646826744594	0.4800629676868159	0.1296036853747853	-0.20792846123899394
	**Modafinil**	0.43782308512645174	0.060259655180366994	-0.07939772065784481	0.9983658282832399	0.35734200032889735	0.3587763882245452	0.07106175264334577	0.3637591125360522
	**Simvastatin**	-2.3396026020118343	-0.6009196734815306	-0.07126502163384621	-0.0667511392631359	0.9262405261031438	0.663177625455899	0.14176747766762632	0.325200364296501
	**Zolpidem**	-0.232844153577925	0.37830357197081405	0.07138213568481636	-0.1747310946934642	0.08664117005962962	-0.41026094213414566	-0.006746861581951156	0.11959973909611807
**Study Design**	**Crossover**	0.042408011590471306	0.12348845992020982	-0.15483523462832635	0.2786774412209926	-0.18799473129498667	0.3882768302427636	0.23626961181479098	0.06568103957380508
	**Parallel**	-0.0254448069542824	-0.07409307595212591	0.09290114077699578	-0.1672064647325957	0.1127968387769922	-0.23296609814565808	-0.1417617670888748	-0.03940862374428318
**Blinding**	**Double-Blind**	-0.010715736669740468	-0.09256134501155015	-0.05449997550176596	-0.06089415621947557	-0.1650177598993561	-0.03454181246258464	0.03771123418834123	0.019791596703591936

	**No Blinding**	-2.278851606012758	0.6733201215633262	0.5932894045117283	1.6463856814842017	0.9187166993439152	-0.5507543471953313	0.387263213356797	-0.6465933338096078
	**Single-Blind**	1.1967823418175692	0.37202192390700195	0.29916359592897773	-0.09422316487277366	1.0465113032487816	0.508450501082964	-0.5451352362954824	0.034762969776120436
	**Triple-Blind**	0.09960165577243377	0.4338629308536762	-0.10161708633436184	-0.24005622734914173	0.288615892145644	0.22468959428109828	-0.05121742453266048	0.18123546018553088
**Sex Distribution**	**Female-dominant**	0.5007868385663887	-0.5059444217560801	0.3230194705163741	-0.5412314637384135	0.4963415057234533	-0.20760238136720535	0.4642516543956921	-0.1454953331222452
	**Male-dominant**	-0.10015736771327777	0.10118888435121612	-0.06460389410327487	0.10824629274768273	-0.09926830114469073	0.0415204762734411	-0.09285033087913837	0.029099066624449204
**Aetiology**	**Mixed Aetiology**	-0.2393176223672116	0.36921415399435126	-0.1098242571273955	-0.2729124583675672	0.05216768141455157	-0.3046341316074609	0.16550375326825256	0.08775807387959667
	**NonTBI**	-0.11376035289444793	-0.1283865320573094	0.03292262838275174	-0.26512506355361326	0.09595852980111166	0.20043047275464215	-0.03287403303516455	0.1455383021821389
	**TBI**	0.1765389876308299	-0.12041381096852047	0.03845081437232184	0.2690187609605902	-0.07406310560783153	0.05210182942640951	-0.06631486011654394	-0.1166481880308676
**Outcome Measures**	**Clinical only**	-0.09695070321788211	-0.21431894054552675	1.399814025521239	0.29014774505807184	-0.35875574081469547	-0.20564536522195004	-0.0016360124615205191	0.9022300564309804
	**Neurobehavioural**	-0.16871039425957943	-0.15876850409649307	-0.05576248406810522	0.04319737611713764	0.025182233844178035	-0.03270595102631957	0.030189581194325837	0.2055611572163824
	**Neurobehavioural & Clinical**	0.49481083699001466	0.3533442457302313	-0.30024624156376256	-0.24415604969164936	0.0995252824340362	-0.06333095333305433	0.11782721968167227	-0.2040594011835043
	**Neurobehavioural & Imaging**	-0.1699895996960309	0.10567084117167985	0.39411273360493676	0.19000842915063162	-0.19698240920267024	0.34783813319625084	-0.3759938141163579	-0.8519099501197699
**Age Category**	**Child/Adolescent**	0.8240673755932173	1.0135182073013034	-0.04249734390740282	0.40452386829028586	0.800401235318488	-0.060667128037958505	0.291505128086892	-0.003766893904398958
	**Middle-Aged**	1.1726173625367469	-0.27520085970255365	-1.2024415756636904	1.6043778157367086	-0.19805722110007692	-1.3155920656998632	-0.5130846075348882	0.3768405763424055
	**Mixed Age**	-0.14601768847478083	-0.10928492428768637	0.0694526674906429	-0.10866846624840695	-0.08456805708412721	0.06061183637473419	-9.354112053118933e-4	-0.027527112435956954
	**Young Adult**	0.09960165577243348	0.43386293085367356	-0.10161708633436331	-0.24005622734914173	0.28861589214564437	0.22468959428109808	-0.051217424532659456	0.18123546018553033
**Treatment Administration**	**Multiple administration**	0.03411524497274036	0.03772963054538696	-0.015332382313644907	-0.033369187341882746	-0.05598555562391275	-0.07351802084070687	-0.0034218506437386368	-0.020901661899594946
	**Single administration**	-0.0828513092195125	-0.09162910275308268	0.037235785618852164	0.08103945497314372	0.13596492080093103	0.17854376489885948	0.008310208706222379	0.05076117889901623
**RoB**	**High**	-0.05646799392869103	0.46414554083575743	-0.0561218685167545	-0.11902236419036918	-0.0012653651598980695	-0.07450941411117462	-0.2722958427446605	-0.10047825494870913
	**Low**	-0.1939659350078812	-0.3155586934475532	0.2005528910637504	0.10980821789270724	0.08575631780151699	-0.06225189521174427	0.0010657175222228952	-0.031267139564245275
	**Some Concerns**	0.3335621867970928	-0.01334740733925262	-0.23038226157431763	-0.03784651851349841	-0.12124366027084024	0.1634406929850951	0.27077338914148513	0.14514559718334513

**Sample Size**	**Small**	-0.1527622429882	0.29918361850429126	0.061647087673136744	-0.13311449535934727	-0.042514370631460886	0.15032191541436507	-0.24600679253409685	0.18085031116376837
	**Medium**	0.06973921862720801	0.11533972764912073	0.07399980924662605	0.09629178508956755	-0.13086708890628296	-0.097392333725044	0.41139930458599255	-0.18252100427981094
	**Large**	0.2631829263063314	-1.0822693380973227	-0.33602906190603465	0.14940834578667808	0.41136546927565393	-0.19425251650815467	-0.24912975584971697	-0.08666609607078829
**Pharmacological Treatment**	**Amantadine**	0.021817236821667415	0.09407574693858393	-0.014378502423588258	0.09221670352214369	0.06135777842725697	0.00543681900150007	3.7090799462690755e-32	
	**Apomorphine**	-0.01787028199994782	0.23655719975917253	-0.0979693575247229	-0.10014649237462342	-0.2097304549764069	-0.006065320038372849	3.7090799462690815e-32	
	**Methylphenidate**	-0.17233023010980708	0.31566665910389913	-0.12030610059309296	-0.11214856158414695	-0.21924365631868317	-0.006289515840242568	3.709079946269074e-32	
	**Modafinil**	-0.41995002470410897	-0.11714649341973715	0.31761197788186246	0.09944523009041951	0.018797417473710415	-0.033248245093676285	3.70907994626907e-32	
	**Simvastatin**	0.5424263052828738	0.2891217088554985	0.07357863933036589	-0.007746461755367934	0.045835696958756375	0.04393465101375807	3.7090799462690815e-32	
	**Zolpidem**	0.06000212590093744	-0.2796503509010599	-0.030293929960529917	-0.1258691602323052	-0.0157354752846041	-0.0026569740177212146	3.709079946269074e-32	
**Study Design**	**Crossover**	-0.023414353040839303	-0.14160657709200947	-0.025623637751919927	0.01980826852056618	0.004712835136183582	0.04004080073831957	7.479617674261058e-34	
	**Parallel**	0.014048611824503633	0.08496394625520566	0.015374182651151893	-0.011884961112339745	-0.002827701081710188	-0.024024480442991856	7.479617674260391e-34	
**Blinding**	**Double-Blind**	0.011611560007541506	-0.001601835266138997	0.004192732580021979	-0.011370602507337516	-0.009362452586734136	-0.007436845509367366	3.00620582759651e-32	
	**No Blinding**	-0.1807535401137353	-0.028625777269485717	-0.013902322317462438	-0.06992277881439903	0.07692483273011293	0.03877159890485829	3.006205827596519e-32	
	**Single-Blind**	-0.02643688900415838	0.08091807810239972	-0.08650586884972442	0.16838193357016687	0.048022871655839794	0.05370847770863758	3.006205827596511e-32	
	**Triple-Blind**	0.0013961179712216527	-0.1011736736125354	0.10305940841647147	-0.0394290381791837	0.014278475692890974	0.002548355865213715	1.8606674464436233e-32	
**Sex Distribution**	**Female-dominant**	-0.3385098392427821	-0.027597409740736642	0.08618530749158605	-0.010844776981630005	0.05515732628004767	0.034085855552016465	2.2116298804328866e-32	
	**Male-dominant**	0.06770196784855638	0.005519481948147327	-0.017237061498317323	0.0021689553963259245	-0.011031465256009622	-0.006817171110403378	2.2116298804328847e-32	

**Aetiology**	**Mixed Aetiology**	-0.011226424555127465	0.28659862791924196	0.18947550151148856	0.14253644143480262	-0.018559888332483735	0.013594154919001815	3.9847531307602573e-32
	**NonTBI**	0.12735979427171265	-0.2600417250101122	-0.18174298284781362	0.13450051883024383	-0.041018081936539955	-0.021208382748583922	3.9847531307602535e-32
	**TBI**	-0.05806668485829261	-0.013278451454565006	-0.00386625933183749	-0.13851848013252313	0.029788985134511826	0.0038071139147908203	3.9847531307602557e-32
**Outcome Measures**	**Clinical only**	0.5620473516843922	-0.10298925212843263	0.1801365876033278	0.12911980116840185	0.2342759717725189	0.03899686904860189	2.0858332882679447e-32
	**Neurobehavioural**	-0.15611887975180164	-0.0141264148590464	-0.02112209651211194	0.018231834247541916	-0.03467496503278966	-0.011202973132535108	2.0858332882679453e-32
	**Neurobehavioural & Clinical**	0.18254532301475276	0.05270578506366936	-0.10222188082667688	-0.09866209339861315	0.07550058921679892	0.030861687384746376	2.0858332882679463e-32
	**Neurobehavioural & Imaging**	0.17611500891743836	-0.005158550075643857	0.24296801617543426	0.06920235991923017	-0.0672766655380859	-0.02244178983386287	2.0858332882679417e-32
**Age Category**	**Child/Adolescent**	0.5364146979618553	-0.05438356404807192	0.007631542311795085	0.06087480747760264	-0.0289177230318716	-0.06143277178654739	2.3806377617827457e-32
	**Middle-Aged**	-0.029567405416730926	0.44053206193642225	-0.19134645208592235	0.12218953677153507	-0.03293196542720932	0.048928817745438086	2.3806377617827602e-32
	**Mixed Age**	-0.05223290542391011	-0.011529563011387155	0.003651197952293047	-0.010225505677377848	0.0038244467899030687	0.0035694184981221954	2.3806377617827522e-32
	**Young Adult**	0.001396117971221908	-0.10117367361253547	0.1030594084164714	-0.03942903817918324	0.014278475692890879	0.0025483558652134716	3.526176142935634e-32
**Treatment Administration**	**Multiple administration**	0.033688042522101405	-0.06414419899903097	0.04257633210946456	0.00974968495234816	-0.04571941224635484	0.024714846366102775	1.2858267934155378e-32
	**Single administration**	-0.08181381755367485	0.15577876899764656	-0.10339966369441383	-0.023677806312845447	0.11103285831257606	-0.060021769746249586	1.285826793415529e-32
**RoB**	**High**	-0.28819718533089705	-0.21193424704012795	-0.006082144577717387	0.06576292351919336	0.030952701895551913	-0.006809625872889652	2.879651461742815e-32
	**Low**	0.09497943797864399	0.11029832527071763	-0.11435978278318433	0.021560341227853164	-0.01931269294247002	0.022520188794381613	2.879651461742819e-32
	**Some Concerns**	0.15251227393283429	0.05436521093910295	0.16945326283940917	-0.09656341098755503	-0.00336314054916608	-0.025362072404798253	2.879651461742815e-32
**Sample Size**	**Small**	-0.059433497047546496	0.1349938207724982	0.0163659318376212	-0.07158456584094224	-0.0018918392818079608	0.01631339506467463	-3.952225330672146e-33

	**Medium**	-0.02059935446180644	-0.01704054034896256	-0.08597378821576371	0.08346552098777812	-0.005844121188698678	-0.019518501322178167	-3.9522253306721504e-33	
	**Large**	0.2097906644198174	-0.33289179133920455	0.1484347109320098	0.009060133840089973	0.01835183069954396	-9.452084529541653e-4	-3.952225330672153e-33	

Values correspond to the coordinates of each variable on the MCA dimensions (see Fig. 3B(ii) in the main text). Categories located farther from the origin contribute more strongly to the corresponding dimension, whereas categories near the origin are frequently observed and are thus less discriminatory.

Supplementary Table 8. MCA category coordinates

Category	Blinding	Aetiology	Outcome Measures	Age Group	Quality (RoB)	Sample Size	Sex Distribution	Study Design	Treatment	Treatment Administration
**Dim 1**	-0.7516240984082525	0.2553681056953005	0.21280704413760634	1.5473977690066976e-4	-0.020074458573848552	0.12403446932522298	0.30549479486166886	0.20369564010777086	0.11423179938445999	0.42132601262454383
**Dim 2**	0.4042392716505711	0.02810291357448229	-0.2214110208364849	0.6703244295255504	-0.012938907529883914	-0.2897479924838861	0.5791693866167329	-0.027229762090809344	-0.6530198465133985	-0.06152845996869993
**Dim 3**	0.5512064128297489	-0.04365667927215987	-0.2850675852707336	0.9413993287861857	-0.09366772459035302	0.05569144603865076	-0.012968924962663777	0.0548863130288218	-0.2291833491114144	0.10705046823381088
**Dim 4**	-0.06538538008158136	-0.10902581624561433	0.4681578880935554	0.3247987228965466	0.06723672864905045	-0.029158743754043648	0.143885026759219	0.05134127312501767	-0.09499770396288482	0.049870834304753246
**Dim 5**	0.54739328420026	0.056187718398372416	0.15962555605439538	-0.26084694250777685	0.01246409841844435	-0.015422972962817711	-0.2172336431689555	-0.036241929931994224	0.04351732681377166	0.037894056863055935
**Dim 6**	-0.357174455454001	-0.011377017006270489	0.7998423421166864	0.4744181038539915	-0.03516266565910172	-0.13461829104113163	0.20673893993199552	-0.04283818904138895	0.18771156070385436	-0.03352085315083539
**Dim 7**	0.8621221015371929	0.0959197400020034	0.20469266574436046	0.3937451119770917	0.06913589378740019	0.08097530377991624	-0.00608462630617203	0.0019310728691794834	-0.027187171845591367	0.01944036125656625
**Dim 8**	-0.11781334528886497	-0.0031243287402218044	-0.24326796461335665	0.44376717167189395	0.060763995725540386	-0.01566249805400139	0.1059687105130771	-0.010231984592189228	0.6178620231480683	-0.01657482714759369
**Dim 9**	-0.24829583627312396	-0.05884632921027654	0.01479003495413056	0.48756717635690416	0.027709419286840193	0.060053300648446474	0.20031473542655548	0.008481602318094453	-0.04488008036253777	-0.02436803212338607
**Dim 10**	0.34666090782811354	0.04013793698950713	0.02148191056497284	0.2657238385411842	0.045079813349650534	-0.22258199731463693	-0.202377768702432	0.024697691984041953	-0.11575006382487431	-0.026949736103847863
**Dim 11**	0.18408398465114456	-0.012816938124107308	0.359479508373577	-0.3192758346037034	-0.02865041300910725	-0.06679405499542394	0.1292077882065496	-0.030967046925665286	0.26242952669415553	0.010951701652603629
**Dim 12**	0.31280303326070275	-0.08967292032019678	0.06979937515854794	0.4150442476073614	-0.015686888270386785	0.037528545172299456	-0.21649258549536537	0.05573548824419844	0.17021993746564407	0.023835133815630485
**Dim 13**	0.5222065337097461	0.02468770186927724	-0.10775765843478786	0.20159796231998206	-0.012250902543073774	0.07932800324597003	0.19853660228938128	-0.037598946258997235	0.23950970954591264	0.03998968258850914
**Dim 14**	0.036960983926536566	-0.01736727647546974	0.01153896590373174	-0.2727394407704974	0.008893127887392068	-0.0471076449396112	-0.08304095254688212	0.07765536604855276	0.19059889499429117	0.052512872029076306
**Dim 15**	-0.042844553320751176	0.022104953372181355	-0.057403256425470076	-0.0684330787964919	-1.5224536031749336e-4	-0.027912414599273756	0.18570066175827687	0.047253922362958084	0.05927265686533695	0.002444179031241871
**Dim 16**	-0.10270082678609115	0.03888272934362265	0.012955465586022175	0.13169550754689496	0.004466734223463571	-0.029445596395610285	-0.05819813324889799	0.013136207914760951	0.11279398693872184	0.01492975849971064
**Dim 17**	-0.048545687784782625	0.019355561619430858	0.19114720096619545	0.11400262627310903	-0.013568491139806266	0.04325260430348815	-0.13540393569711284	-0.004682870608167835	0.0023491885319358017	-0.024062887515786724

**Dim 18**	-0.0126208020114401	0.004426150484854936	-0.017392107999863383	0.06836131531610694	-0.015756903610102457	-0.07164617030522297	-0.011038963896294659	-0.028321315418401903	0.0897707450560595	0.045817284999307796
**Dim 19**	0.0017109874573266529	0.0012887531106124768	0.07494015660999331	-0.01925107585134071	0.016337111826169152	0.02627561818462243	0.03447412299663437	-0.005124727550384017	0.021373787785049052	-0.03041166579247464
**Dim 20**	0.011914878517311656	0.0461728267108411	0.0294729754841402	0.03335245009814416	-0.003080048746836174	0.006980362995641954	-0.00433791079265204	0.003961653704113218	-0.025708123722313382	-0.0069640606802486430
**Dim 21**	0.03246593187302739	-0.009929661711503954	0.05195623260461056	-0.01093669149407174	0.0027589561346386053	0.0035386234096791073	0.0220629305120190 26	9.425670272366971e-4	-0.05311978228666174	0.03265672303311061
**Dim 22**	0.021897896742335554	-0.001269037971597096	0.00905344836673757	-0.0015965449194434084	-0.003217169827768763	-0.0013834382368192333	0.0136343422208065 44	0.008008160147663855	1.852358375408714e-4	-0.0176534616900734 03
**Dim 23**	2.719821232308291e-32	3.9847531307602557e-32	2.0858332882679444e-32	2.667022357070973e-32	2.879651461742816e-32	-3.95222533067215e-33	2.2116298804328855e-32	7.479617674260724e-34	3.709079946269076e-32	1.2858267934155335e-32

Values represent the relative contribution of each category modality to the construction of each MCA dimension. Higher values indicate categories contributing the most to the corresponding dimension.

Supplementary Table 9. Cosine squared (cos^2) values for category and variable representations across MCA dimensions

Category	Variable	Dim 1	Dim 2	Dim 3	Dim 4	Dim 5	Dim 6	Dim 7	Dim 8
**Pharmacological Treatment**	**Amantadine**	-0.6335734451871757	0.4129607536574133	0.07994908781607307	-0.06565586815959568	-0.43826975507780835	-0.31428736121479695	0.3089102211185886	-0.12528432342814463
	**Apomorphine**	1.419183214092111	-2.073075635121444	0.03208434778446505	-0.8367427371355716	-0.36611130116676494	-1.3480075992886982	1.317780428358757	1.7404246724274277
	**Methylphenidate**	0.1946802087243169	-1.7175042252523884	-0.814498112332845	1.2145788372087893	-0.006003611620632999	1.5024463964604742	1.1213824471174434	-0.9498002846747379
	**Modafinil**	-0.3158009622348348	-0.14734856385539433	-0.5462745492817124	-2.2670451825122933	0.6225287360052074	1.3706760231943034	-1.130338286162726	-0.04732097391669067
	**Simvastatin**	-0.9410089539335772	-0.6687121304442517	-0.44909118167485174	1.0050435183752342	-0.13521818484251755	0.05819493369349984	-1.2946872846611017	3.3320209894831465
	**Zolpidem**	0.9619107348459196	0.2755607219356744	0.32273031302038474	0.3798352084461284	0.5841780775851464	-0.14275302862165645	-0.48617055684450944	-0.24286794100259124
**Study Design**	**Crossover**	1.0184782005388542	-0.13614881045404661	0.27443156514410894	0.25670636562508864	-0.18120964965997133	-0.21419094520694515	0.009655364345897631	-0.05115992296094563
	**Parallel**	-0.6110869203233125	0.08168928627242793	-0.16465893908646534	-0.1540238193750533	0.10872578979598288	0.12851456712416726	-0.0057932186075386645	0.030695953776567177
**Blinding**	**Double-Blind**	0.21189630007162452	-0.10142694527825648	-0.09810774044269217	-0.038104415614794225	-0.2019806380318473	0.1502785562407141	-0.14754697727688282	0.05059328894449961
	**No Blinding**	-0.5416455365937822	1.2722893424009554	-1.2505470393474878	-0.9110166604593691	0.14551149760228516	-1.1631538786305575	2.5532299022550142	-0.5748683166258552
	**Single-Blind**	-1.0195333077278568	0.3101548736845878	-0.3407785829078458	0.9855254170074162	1.6480589858040569	-1.4265947467575664	-0.6450958378879985	-0.4900191087900295
	**Triple-Blind**	-1.6572138493829955	0.13593981579499767	3.8942590140170217	-0.2979458612595784	0.5979832914265452	1.0107722473314056	1.687901319058639	0.5430407553159252
**Sex Distribution**	**Female-dominant**	0.763736987154172	1.4479234665418326	-0.032422312406659534	0.3597125668980475	-0.5430841079223887	0.5168473498299888	-0.01521156576543002	0.2649217762826927
	**Male-dominant**	-0.15274739743083438	-0.2895846933083666	0.006484462481331981	-0.07194251337960952	0.10861682158447773	-0.1033694699659978	0.0030423131530859593	-0.0529843552565385
**Aetiology**	**Mixed Aetiology**	1.0770413723166892	-0.9695851128129628	0.31208381388830575	-0.09653661758574954	0.11919543537454197	-0.42880214355375984	-8.125900382243246e-4	-0.21290563579200414
	**NonTBI**	0.4551672618551138	1.1382025942598566	-0.5740238895212652	-0.5576182798879367	0.21793087501569267	0.36054004151613855	0.5763310300502449	0.19415966335067275
	**TBI**	-0.7661043170859015	-0.08430874072344686	0.13097003781647978	0.3270774487368432	-0.16856315519511736	0.03413105101880984	-0.28775922000601034	0.009372986220666001
**Outcome Measures**	**Clinical only**	-0.7474039591458191	-1.4668986250985268	-1.2805048913954986	2.1771780436285066	0.14965831849222008	2.714449529627062	1.0556282155370589	-1.3743246765508452
	**Neurobehavioural**	-0.4511414440598839	0.18816397347138922	0.20355018718477083	0.1782303639274482	-0.2229043019348274	-0.4624516461241458	-0.06155351490207152	-0.06797213734737179
	**Neurobehavioural & Clinical**	0.30468781223860153	-0.7822229027815051	-0.4597502728582236	-1.0746908576891947	0.27858576187159867	0.3059196870731632	0.11067770202152624	0.30608657769322584

	**Neurobehavioural & Imaging**	1.7450857675175269	1.1753134710627031	0.3964346359860168	0.5919140025074616	0.43316244578859014	0.6414517978906662	-0.2859817396790717	0.1631383777515646
**Age Category**	**Child/Adolescent**	-0.3843936137812493	0.5455228117258094	0.13740818497110097	0.3446695300365172	-2.6671075725738236	-0.06881498239410601	-0.5789464763348449	-0.251902042410332
	**Middle-Aged**	2.0220277562363926	2.169667750046027	-0.060648541732019355	1.3389904028946529	0.8304472825580161	1.0519708048482361	0.5184484457937055	1.564096639270257
	**Mixed Age**	0.02019866603545503	-0.1698326594646323	-0.20542134211136023	-0.08651918008540545	0.19528922855815434	-0.09625565436957163	-0.05242284060913267	-0.08016666548827611
	**Young Adult**	-1.6572138493829955	0.13593981579499767	3.8942590140170217	-0.29794586125957806	0.5979832914265455	1.0107722473314078	1.6879013190586387	0.5430407553159269
**Treatment Administration**	**Multiple administration**	-0.5898564176743615	0.08613984395618005	-0.14987065552733528	-0.06981916802665446	-0.053051679608278265	0.04692919441116953	-0.027216505759192655	0.023204758006631118
	**Single administration**	1.4325084429234491	-0.2091967638935799	0.36397159199495704	0.16956083663616095	0.12883979333439013	-0.1139709007128403	0.06609722827232516	-0.0563544123018185
**RoB**	**High**	0.00673345497648787	-1.010371920814233	-0.3849281324463562	0.4424091985168853	-0.4790923332168947	0.18395535050400683	0.2116651222317777	0.5445531595750963
	**Low**	0.14052121001694043	0.09057235270918791	0.6556740721324708	-0.4706571005433533	-0.08724868892911085	0.24613865961371217	-0.48395125651180204	-0.4253479700787832
	**Some Concerns**	-0.20747804071497394	0.8809828455153933	-0.5517491134571736	0.2299580879736193	0.6037333174013386	-0.5355820070950241	0.4796938156422249	0.0630867976803081
**Sample Size**	**Small**	0.36371303161074836	0.7088655813962882	-0.1755278920974329	-0.20808945901240194	-0.40928002728812196	0.11554828397590747	0.06407339460110979	-0.09245278730929098
	**Medium**	-0.8068809706355822	-0.2970166319934766	0.11207957844364395	0.3613062276270676	0.6100071733141329	0.16131630093244573	-0.28404348151335274	0.1670238975626105
	**Large**	0.8152713470005026	-1.28109292685447	0.23052265176974124	-0.2406929998767966	-0.24699606491446405	-0.680719458031748	0.4628959982519917	-0.12155860441532368
**Pharmacological Treatment**	**Amantadine**	0.11795238152890414	-0.1371764901230562	-0.1279736024710429	-0.0866961452450564	-0.29957119606366067	0.04791329269277605	-0.05158202097184487	-0.15501930075060988
	**Apomorphine**	1.6983923035318955	-0.1311516436703999	2.4255675981484104	0.455469608178039	-0.3150589258269935	0.003924038039856571	0.07153190806006031	0.23115246769326356
	**Methylphenidate**	0.04899850322728123	-0.2638158028254402	-0.6437362289055595	-0.10433743246575812	0.6814646826744594	0.4800629676868159	0.1296036853747853	-0.20792846123899394
	**Modafinil**	0.43782308512645174	0.060259655180366994	-0.07939772065784481	0.9983658282832399	0.35734200032889735	0.3587763882245452	0.07106175264334577	0.3637591125360522
	**Simvastatin**	-2.3396026020118343	-0.6009196734815306	-0.07126502163384621	-0.0667511392631359	0.9262405261031438	0.663177625455899	0.14176747766762632	0.325200364296501
	**Zolpidem**	-0.232844153577925	0.37830357197081405	0.07138213568481636	-0.1747310946934642	0.08664117005962962	-0.41026094213414566	-0.006746861581951156	0.11959973909611807
**Study Design**	**Crossover**	0.042408011590471306	0.12348845992020982	-0.15483523462832635	0.2786774412209926	-0.18799473129498667	0.3882768302427636	0.23626961181479098	0.06568103957380508
	**Parallel**	-0.0254448069542824	-0.07409307595212591	0.09290114077699578	-0.1672064647325957	0.1127968387769922	-0.23296609814565808	-0.1417617670888748	-0.03940862374428318
**Blinding**	**Double-Blind**	-0.010715736669740468	-0.09256134501155015	-0.05449997550176596	-0.06089415621947557	-0.1650177598993561	-0.03454181246258464	0.03771123418834123	0.019791596703591936

	**No Blinding**	-2.278851606012758	0.6733201215633262	0.5932894045117283	1.6463856814842017	0.9187166993439152	-0.5507543471953313	0.387263213356797	-0.6465933338096078
	**Single-Blind**	1.1967823418175692	0.37202192390700195	0.29916359592897773	-0.09422316487277366	1.0465113032487816	0.508450501082964	-0.5451352362954824	0.034762969776120436
	**Triple-Blind**	0.09960165577243377	0.4338629308536762	-0.10161708633436184	-0.24005622734914173	0.288615892145644	0.22468959428109828	-0.05121742453266048	0.18123546018553088
**Sex Distribution**	**Female-dominant**	0.5007868385663887	-0.5059444217560801	0.3230194705163741	-0.5412314637384135	0.4963415057234533	-0.20760238136720535	0.4642516543956921	-0.1454953331222452
	**Male-dominant**	-0.10015736771327777	0.10118888435121612	-0.06460389410327487	0.10824629274768273	-0.09926830114469073	0.0415204762734411	-0.09285033087913837	0.029099066624449204
**Aetiology**	**Mixed Aetiology**	-0.2393176223672116	0.36921415399435126	-0.1098242571273955	-0.2729124583675672	0.05216768141455157	-0.3046341316074609	0.16550375326825256	0.08775807387959667
	**NonTBI**	-0.11376035289444793	-0.1283865320573094	0.03292262838275174	-0.26512506355361326	0.09595852980111166	0.20043047275464215	-0.03287403303516455	0.1455383021821389
	**TBI**	0.1765389876308299	-0.12041381096852047	0.03845081437232184	0.2690187609605902	-0.07406310560783153	0.05210182942640951	-0.06631486011654394	-0.1166481880308676
**Outcome Measures**	**Clinical only**	-0.09695070321788211	-0.21431894054552675	1.399814025521239	0.29014774505807184	-0.35875574081469547	-0.20564536522195004	-0.0016360124615205191	0.9022300564309804
	**Neurobehavioural**	-0.16871039425957943	-0.15876850409649307	-0.05576248406810522	0.04319737611713764	0.025182233844178035	-0.03270595102631957	0.030189581194325837	0.2055611572163824
	**Neurobehavioural & Clinical**	0.49481083699001466	0.3533442457302313	-0.30024624156376256	-0.24415604969164936	0.0995252824340362	-0.06333095333305433	0.11782721968167227	-0.2040594011835043
	**Neurobehavioural & Imaging**	-0.1699895996960309	0.10567084117167985	0.39411273360493676	0.19000842915063162	-0.19698240920267024	0.34783813319625084	-0.3759938141163579	-0.8519099501197699
**Age Category**	**Child/Adolescent**	0.8240673755932173	1.0135182073013034	-0.04249734390740282	0.40452386829028586	0.800401235318488	-0.060667128037958505	0.291505128086892	-0.003766893904398958
	**Middle-Aged**	1.1726173625367469	-0.27520085970255365	-1.2024415756636904	1.6043778157367086	-0.19805722110007692	-1.3155920656998632	-0.5130846075348882	0.3768405763424055
	**Mixed Age**	-0.14601768847478083	-0.10928492428768637	0.0694526674906429	-0.10866846624840695	-0.08456805708412721	0.06061183637473419	-9.354112053118933e-4	-0.027527112435956954
	**Young Adult**	0.09960165577243348	0.43386293085367356	-0.10161708633436331	-0.24005622734914173	0.28861589214564437	0.22468959428109808	-0.051217424532659456	0.18123546018553033
**Treatment Administration**	**Multiple administration**	0.03411524497274036	0.03772963054538696	-0.015332382313644907	-0.033369187341882746	-0.05598555562391275	-0.07351802084070687	-0.0034218506437386368	-0.020901661899594946
	**Single administration**	-0.0828513092195125	-0.09162910275308268	0.037235785618852164	0.08103945497314372	0.13596492080093103	0.17854376489885948	0.008310208706222379	0.050761178899901623
**RoB**	**High**	-0.05646799392869103	0.46414554083575743	-0.0561218685167545	-0.11902236419036918	-0.0012653651598980695	-0.07450941411117462	-0.2722958427446605	-0.10047825494870913
	**Low**	-0.1939659350078812	-0.3155586934475532	0.2005528910637504	0.10980821789270724	0.08575631780151699	-0.06225189521174427	0.0010657175222228952	-0.031267139564245275
	**Some Concerns**	0.3335621867970928	-0.01334740733925262	-0.23038226157431763	-0.03784651851349841	-0.12124366027084024	0.1634406929850951	0.27077338914148513	0.14514559718334513
**Sample Size**	**Small**	-0.1527622429882	0.29918361850429126	0.061647087673136744	-0.13311449535934727	-0.042514370631460886	0.15032191541436507	-0.24600679253409685	0.18085031116376837

	**Medium**	0.06973921862720801	0.11533972764912073	0.07399980924662605	0.09629178508956755	-0.13086708890628296	-0.097392333725044	0.41139930458599255	-0.18252100427981094
	**Large**	0.2631829263063314	-1.0822693380973227	-0.33602906190603465	0.14940834578667808	0.41136546927565393	-0.19425251650815467	-0.24912975584971697	-0.08666609607078829
**Pharmacological Treatment**	**Amantadine**	0.021817236821667415	0.09407574693858393	-0.014378502423588258	0.09221670352214369	0.06135777842725697	0.00543681900150007	3.7090799462690755e-32	
	**Apomorphine**	-0.01787028199994782	0.23655719975917253	-0.0979693575247229	-0.10014649237462342	-0.2097304549764069	-0.006065320038372849	3.7090799462690815e-32	
	**Methylphenidate**	-0.17233023010980708	0.31566665910389913	-0.12030610059309296	-0.11214856158414695	-0.21924365631868317	-0.006289515840242568	3.709079946269074e-32	
	**Modafinil**	-0.41995002470410897	-0.11714649341973715	0.31761197788186246	0.09944523009041951	0.018797417473710415	-0.033248245093676285	3.70907994626907e-32	
	**Simvastatin**	0.5424263052828738	0.2891217088554985	0.07357863933036589	-0.007746461755367934	0.045835696958756375	0.04393465101375807	3.7090799462690815e-32	
	**Zolpidem**	0.06000212590093744	-0.2796503509010599	-0.030293929960529917	-0.1258691602323052	-0.0157354752846041	-0.0026569740177212146	3.709079946269074e-32	
**Study Design**	**Crossover**	-0.023414353040839303	-0.14160657709200947	-0.025623637751919927	0.01980826852056618	0.004712835136183582	0.04004080073831957	7.479617674261058e-34	
	**Parallel**	0.014048611824503633	0.08496394625520566	0.015374182651151893	-0.011884961112339745	-0.002827701081710188	-0.024024480442991856	7.479617674260391e-34	
**Blinding**	**Double-Blind**	0.011611560007541506	-0.001601835266138997	0.004192732580021979	-0.011370602507337516	-0.009362452586734136	-0.007436845509367366	3.00620582759651e-32	
	**No Blinding**	-0.1807535401137353	-0.028625777269485717	-0.013902322317462438	-0.06992277881439903	0.07692483273011293	0.03877159890485829	3.006205827596519e-32	
	**Single-Blind**	-0.02643688900415838	0.08091807810239972	-0.08650586884972442	0.16838193357016687	0.048022871655839794	0.05370847770863758	3.006205827596511e-32	
	**Triple-Blind**	0.0013961179712216527	-0.1011736736125354	0.10305940841647147	-0.0394290381791837	0.014278475692890974	0.002548355865213715	1.8606674464436233e-32	
**Sex Distribution**	**Female-dominant**	-0.3385098392427821	-0.027597409740736642	0.08618530749158605	-0.010844776981630005	0.05515732628004767	0.034085855552016465	2.2116298804328866e-32	
	**Male-dominant**	0.06770196784855638	0.005519481948147327	-0.017237061498317323	0.0021689553963259245	-0.011031465256009622	-0.006817171110403378	2.2116298804328847e-32	
**Aetiology**	**Mixed Aetiology**	-0.011226424555127465	0.28659862791924196	0.18947550151148856	0.14253644143480262	-0.018559888332483735	0.013594154919001815	3.9847531307602573e-32	

	**NonTBI**	0.12735979427171265	-0.2600417250101122	-0.18174298284781362	0.13450051883024383	-0.041018081936539955	-0.021208382748583922	3.9847531307602535e-32
	**TBI**	-0.05806668485829261	-0.013278451454565006	-0.00386625933183749	-0.13851848013252313	0.029788985134511826	0.0038071139147908203	3.9847531307602557e-32
**Outcome Measures**	**Clinical only**	0.5620473516843922	-0.10298925212843263	0.1801365876033278	0.12911980116840185	0.2342759717725189	0.03899686904860189	2.0858332882679447e-32
	**Neurobehavioural**	-0.15611887975180164	-0.0141264148590464	-0.02112209651211194	0.018231834247541916	-0.03467496503278966	-0.011202973132535108	2.0858332882679453e-32
	**Neurobehavioural & Clinical**	0.18254532301475276	0.05270578506366936	-0.10222188082667688	-0.09866209339861315	0.07550058921679892	0.030861687384746376	2.0858332882679463e-32
	**Neurobehavioural & Imaging**	0.17611500891743836	-0.005158550075643857	0.24296801617543426	0.06920235991923017	-0.0672766655380859	-0.02244178983386287	2.0858332882679417e-32
**Age Category**	**Child/Adolescent**	0.5364146979618553	-0.05438356404807192	0.007631542311795085	0.06087480747760264	-0.0289177230318716	-0.06143277178654739	2.3806377617827457e-32
	**Middle-Aged**	-0.029567405416730926	0.44053206193642225	-0.19134645208592235	0.12218953677153507	-0.03293196542720932	0.048928817745438086	2.3806377617827602e-32
	**Mixed Age**	-0.05223290542391011	-0.011529563011387155	0.003651197952293047	-0.010225505677377848	0.0038244467899030687	0.0035694184981221954	2.3806377617827522e-32
	**Young Adult**	0.001396117971221908	-0.10117367361253547	0.1030594084164714	-0.03942903817918324	0.014278475692890879	0.0025483558652134716	3.526176142935634e-32
**Treatment Administration**	**Multiple administration**	0.033688042522101405	-0.06414419899903097	0.04257633210946456	0.00974968495234816	-0.04571941224635484	0.024714846366102775	1.2858267934155378e-32
	**Single administration**	-0.08181381755367485	0.15577876899764656	-0.10339966369441383	-0.023677806312845447	0.11103285831257606	-0.060021769746249586	1.285826793415529e-32
**RoB**	**High**	-0.28819718533089705	-0.21193424704012795	-0.006082144577717387	0.06576292351919336	0.030952701895551913	-0.006809625872889652	2.879651461742815e-32
	**Low**	0.09497943797864399	0.11029832527071763	-0.11435978278318433	0.021560341227853164	-0.01931269294247002	0.022520188794381613	2.879651461742819e-32
	**Some Concerns**	0.15251227393283429	0.05436521093910295	0.16945326283940917	-0.09656341098755503	-0.00336314054916608	-0.025362072404798253	2.879651461742815e-32
**Sample Size**	**Small**	-0.059433497047546496	0.1349938207724982	0.0163659318376212	-0.07158456584094224	-0.0018918392818079608	0.01631339506467463	-3.952225330672146e-33
	**Medium**	-0.02059935446180644	-0.01704054034896256	-0.08597378821576371	0.08346552098777812	-0.005844121188698678	-0.019518501322178167	-3.9522253306721504e-33

	**Large**	0.2097906644198174	-0.33289179133920455	0.1484347109320098	0.009060133840089973	0.01835183069954396	-9.452084529541653e-4	-3.952225330672153e-33	

The cosine squared ($\cos^2$) values indicate how well each category is represented by an MCA dimension. Higher $\cos^2$ values reflect better projection of the category onto the corresponding dimension, while low values indicate poor representation and should be interpreted with caution.

References

1. Porcaro C, Nemirovsky IE, Riganello F, et al. Diagnostic Developments in Differentiating Unresponsive Wakefulness Syndrome and the Minimally Conscious State. *Front Neurol*. 2021;12:778951. doi:10.3389/fneur.2021.778951
2. Schnakers C, Vanhaudenhuyse A, Giacino J, et al. Diagnostic accuracy of the vegetative and minimally conscious state: clinical consensus versus standardized neurobehavioral assessment. *BMC Neurol*. Jul 21 2009;9:35. doi:10.1186/1471-2377-9-35
3. Stender J, Gosseries O, Bruno MA, et al. Diagnostic precision of PET imaging and functional MRI in disorders of consciousness: a clinical validation study. *Lancet*. Aug 9 2014;384(9942):514-22. doi:10.1016/s0140-6736(14)60042-8
4. Teasdale G, Jennett B. Assessment of coma and impaired consciousness. A practical scale. *Lancet*. Jul 13 1974;2(7872):81-4. doi:10.1016/s0140-6736(74)91639-0
5. Giacino JT, Kalmar K, Whyte J. The JFK Coma Recovery Scale-Revised: Measurement characteristics and diagnostic utility11No commercial party having a direct financial interest in the results of the research supporting this article has or will confer a benefit upon the authors or upon any organization with which the authors are associated. *Archives of Physical Medicine and Rehabilitation*. 2004/12/01/ 2004;85(12):2020-2029. doi:10.1016/j.apmr.2004.02.033
6. Bodien YG, Chatelle C, Taubert A, Uchani S, Giacino JT, Ehrlich-Jones L. Updated Measurement Characteristics and Clinical Utility of the Coma Recovery Scale-Revised Among Individuals With Acquired Brain Injury. *Archives of Physical Medicine and Rehabilitation*. 2021;102(1):169-171. doi:10.1016/j.apmr.2020.09.369
7. Aubinet C, Cassol H, Bodart O, et al. Simplified evaluation of CONsciousness disorders (SECONDs) in individuals with severe brain injury: A validation study. *Ann Phys Rehabil Med*. Sep 2021;64(5):101432. doi:10.1016/j.rehab.2020.09.001
8. Bodien YG, Vora I, Barra A, et al. Feasibility and Validity of the Coma Recovery Scale-Revised for Accelerated Standardized Testing: A Practical Assessment Tool for Detecting Consciousness in the Intensive Care Unit. *Ann Neurol*. Nov 2023;94(5):919-924. doi:10.1002/ana.26740
9. Rappaport M, Hall KM, Hopkins K, Belleza T, Cope DN. Disability rating scale for severe head trauma: coma to community. *Arch Phys Med Rehabil*. Mar 1982;63(3):118-23.
10. Rappaport M. The Disability Rating and Coma/Near-Coma scales in evaluating severe head injury. *Neuropsychol Rehabil*. Jul-Sep 2005;15(3-4):442-53. doi:10.1080/09602010443000335
11. Jennett B, Snoek J, Bond MR, Brooks N. Disability after severe head injury: observations on the use of the Glasgow Outcome Scale. *J Neurol Neurosurg Psychiatry*. Apr 1981;44(4):285-93. doi:10.1136/jnnp.44.4.285

12. Wilson JT, Pettigrew LE, Teasdale GM. Structured interviews for the Glasgow Outcome Scale and the extended Glasgow Outcome Scale: guidelines for their use. *J Neurotrauma*. Aug 1998;15(8):573-85. doi:10.1089/neu.1998.15.573
13. Ansell BJ, Keenan JE. The Western Neuro Sensory Stimulation Profile: a tool for assessing slow-to-recover head-injured patients. *Arch Phys Med Rehabil*. Feb 1989;70(2):104-8.
14. Shiel A, Horn SA, Wilson BA, Watson MJ, Campbell MJ, McLellan DL. The Wessex Head Injury Matrix (WHIM) main scale: a preliminary report on a scale to assess and monitor patient recovery after severe head injury. *Clin Rehabil*. Aug 2000;14(4):408-16. doi:10.1191/0269215500cr326oa
15. Flannery J. Psychometric properties of a cognitive functioning scale for patients with traumatic brain injury. *West J Nurs Res*. Aug 1993;15(4):465-77; discussion 477-82. doi:10.1177/019394599301500406
16. Flannery J. Using the levels of cognitive functioning assessment scale with patients with traumatic brain injury in an acute care setting. *Rehabil Nurs*. Mar-Apr 1998;23(2):88-94. doi:10.1002/j.2048-7940.1998.tb02136.x
17. Magee WL, Siegert RJ, Taylor SM, Daveson BA, Lenton-Smith G. Music Therapy Assessment Tool for Awareness in Disorders of Consciousness (MATADOC): Reliability and Validity of a Measure to Assess Awareness in Patients with Disorders of Consciousness. *J Music Ther*. Spring 2016;53(1):1-26. doi:10.1093/jmt/thv017
18. Folstein MF, Folstein SE, McHugh PR. "Mini-mental state". A practical method for grading the cognitive state of patients for the clinician. *J Psychiatr Res*. 1975;12(3):189-98. doi:10.1016/0022-3956(75)90026-6
19. van Swieten JC, Koudstaal PJ, Visser MC, Schouten HJ, van Gijn J. Interobserver agreement for the assessment of handicap in stroke patients. *Stroke*. May 1988;19(5):604-7. doi:10.1161/01.str.19.5.604
20. Safar P. Cerebral resuscitation after cardiac arrest: a review. *Circulation*. Dec 1986;74(6 Pt 2):Iv138-53.
21. Stokes V, Gunn S, Schouwenaars K, Badwan D. Neurobehavioural assessment and diagnosis in disorders of consciousness: a preliminary study of the Sensory Tool to Assess Responsiveness (STAR). *Neuropsychol Rehabil*. Sep 2018;28(6):966-983. doi:10.1080/09602011.2016.1214604
22. Dodds TA, Martin DP, Stolov WC, Deyo RA. A validation of the Functional Independence Measurement and its performance among rehabilitation inpatients. *Archives of Physical Medicine and Rehabilitation*. 1993/05/01/ 1993;74(5):531-536. doi:10.1016/0003-9993(93)90119-U
23. Seel RT, Sherer M, Whyte J, et al. Assessment scales for disorders of consciousness: evidence-based recommendations for clinical practice and research. *Arch Phys Med Rehabil*. Dec 2010;91(12):1795-813. doi:10.1016/j.apmr.2010.07.218
24. Gill-Thwaites H, Munday R. The Sensory Modality Assessment and Rehabilitation Technique (SMART): a valid and reliable assessment for vegetative state and minimally conscious state patients. *Brain Inj*. Dec 2004;18(12):1255-69. doi:10.1080/02699050410001719952
25. Coppler PJ, Gagnon DJ, Flickinger KL, et al. A multicenter, randomized, doubleblind, placebo-controlled trial of amantadine to stimulate awakening in comatose patients resuscitated from cardiac arrest. *Clin Exp Emerg Med*. Jun 2024;11(2):205-212. doi:10.15441/ceem.23.158

26. Gatto LAM, Demartini Z, Jr., Telles JPM, Figueiredo EG. Does amantadine improve cognitive recovery in severe disorders of consciousness after aneurysmal subarachnoid hemorrhage? A double-blind placebo-controlled study. *Clin Neurol Neurosurg*. Feb 2024;237:108135. doi:10.1016/j.clineuro.2024.108135
27. Shimia M, Iranmehr A, Valizadeh A, et al. A placebo-controlled randomized clinical trial of amantadine hydrochloride for evaluating the functional improvement of patients following severe acute traumatic brain injury. *J Neurosurg Sci*. Oct 2023;67(5):598-604. doi:10.23736/s0390-5616.21.05266-8
28. Shafiee S, Ehteshami S, Moosazadeh M, Aghapour S, Haddadi K. Placebo-controlled trial of oral amantadine and zolpidem efficacy on the outcome of patients with acute severe traumatic brain injury and diffuse axonal injury. *Caspian J Intern Med*. Winter 2022;13(1):113-121. doi:10.22088/cjim.13.1.113
29. Abbasivash R, Valizade Hasanloei MA, Kazempour A, Mahdkhah A, Shaaf Ghoreishi MM, Akhavan Masoumi G. The Effect of Oral Administration of Amantadine on Neurological Outcome of Patients With Diffuse Axonal Injury in ICU. *J Exp Neurosci*. 2019;13:1179069518824851. doi:10.1177/1179069518824851
30. Akçıl EF, Dilmen Ö K, Vehid H, Tunalı Y. Can Amantadine Ameliorate Neurocognitive Functions After Subarachnoid Haemorrhage? A Preliminary Study. *Turk J Anaesthesiol Reanim*. Apr 2018;46(2):100-107. doi:10.5152/tjar.2018.20280
31. Ghalaenovi H, Fattahi A, Koohpayehzadeh J, et al. The effects of amantadine on traumatic brain injury outcome: a double-blind, randomized, controlled, clinical trial. *Brain Inj*. 2018;32(8):1050-1055. doi:10.1080/02699052.2018.1476733
32. Giacino JT, Whyte J, Bagiella E, et al. Placebo-controlled trial of amantadine for severe traumatic brain injury. *N Engl J Med*. Mar 1 2012;366(9):819-26. doi:10.1056/NEJMoa1102609
33. McMahon MA, Vargus-Adams JN, Michaud LJ, Bean J. Effects of amantadine in children with impaired consciousness caused by acquired brain injury: a pilot study. *Am J Phys Med Rehabil*. Jul 2009;88(7):525-32. doi:10.1097/PHM.0b013e3181a5ade3
34. Patrick PD, Blackman JA, Mabry JL, Buck ML, Gurka MJ, Conaway MR. Dopamine agonist therapy in low-response children following traumatic brain injury. *J Child Neurol*. Oct 2006;21(10):879-85. doi:10.1177/08830738060210100901
35. Meythaler JM, Brunner RC, Johnson A, Novack TA. Amantadine to improve neurorecovery in traumatic brain injury-associated diffuse axonal injury: a pilot double-blind randomized trial. *J Head Trauma Rehabil*. Aug 2002;17(4):300-13. doi:10.1097/00001199-200208000-00004
36. Othman MH, Toury-Puel AG, Hansen KIT, et al. Stimulants for disorders of consciousness in the intensive care unit: a randomized, placebo-controlled trial. *Brain*. 2025;148(10):3523-3536. doi:10.1093/brain/awaf228
37. Moein H, Khalili HA, Keramatian K. Effect of methylphenidate on ICU and hospital length of stay in patients with severe and moderate traumatic brain injury. *Clin Neurol Neurosurg*. Sep 2006;108(6):539-42. doi:10.1016/j.clineuro.2005.09.003
38. Talebi Kiasari F, Naghshbandi M, Emamikhah M, et al. Evaluation of the effect of Modafinil in the improvement of the level of consciousness in patients with COVID-19 encephalopathy: A randomized controlled trial. *Neuropsychopharmacol Rep*. Sep 2024;44(3):490-501. doi:10.1002/npr2.12447

39. Zand Z, Zand F, Asmarian N, et al. Efficacy of oral modafinil on accelerating consciousness recovery in adult patients with moderate to severe acute traumatic brain injury admitted to intensive care unit: a randomized double-blind clinical trial. *Neurosurg Rev*. Dec 26 2024;48(1):2. doi:10.1007/s10143-024-03145-x
40. Rahat Dahmardeh A, Khoshfetrat M, Heidari M, Keykha A. The Effectiveness of Zolpidem in Improving Consciousness in Patients with Acute Brain Injury. *Archives of Anesthesiology and Critical Care*. 2024;10(1):9-14. doi:10.18502/aacc.v10i1.14766
41. Chatelle C, Thibaut A, Gosseries O, et al. Changes in cerebral metabolism in patients with a minimally conscious state responding to zolpidem. Article. *Frontiers in Human Neuroscience*. 2014;8(DEC)917. doi:10.3389/fnhum.2014.00917
42. Machado C, Estévez M, Rodríguez R, et al. Zolpidem arousing effect in persistent vegetative state patients: autonomic, EEG and behavioral assessment. *Curr Pharm Des*. 2014;20(26):4185-202. doi:10.2174/13816128113196660646
43. Whyte J, Rajan R, Rosenbaum A, et al. Zolpidem and restoration of consciousness. *Am J Phys Med Rehabil*. Feb 2014;93(2):101-13. doi:10.1097/phm.0000000000000069
44. Whyte J, Myers R. Incidence of clinically significant responses to zolpidem among patients with disorders of consciousness: A preliminary placebo controlled trial. Article. *American Journal of Physical Medicine and Rehabilitation*. 2009;88(5):410-418. doi:10.1097/PHM.0b013e3181a0e3a0
45. Brefel-Courbon C, Payoux P, Ory F, et al. Clinical and imaging evidence of zolpidem effect in hypoxic encephalopathy. *Ann Neurol*. Jul 2007;62(1):102-5. doi:10.1002/ana.21110
46. Shafiee S, Zali A, Shafizad M, et al. The Effect of Oral Simvastatin on the Clinical Outcome of Patients with Severe Traumatic Brain Injury: A Randomized Clinical Trial. *Ethiop J Health Sci*. Jul 2021;31(4):807-816. doi:10.4314/ejhs.v31i4.15
47. Ayhan YE, Özgan B, İlerler EE, Karakurt S. Clinical Course of Amantadine in Patients with Prolonged Consciousness Disorder in the Intensive Care Unit. *İstanbul Journal of Pharmacy*. 2025-09-23T10:07:21 2025;55(2):173-182. doi:10.26650/IstanbulJPharm.2025.1579713
48. Blum C, Single C, Laichinger K, et al. Retrospective analysis of amantadine response and predictive factors in intensive care unit patients with non-traumatic disorders of consciousness. *Front Neurol*. 2025;15:1512227. doi:10.3389/fneur.2024.1512227
49. Hintze TD, Small CE, Montgomery J, Reveles KR, Hafeez S, Barthol CA. Comparison of Amantadine, Modafinil, and Standard of Care in the Acute Treatment of Disorders of Consciousness After Severe Traumatic Brain Injury. *Clin Neuropharmacol*. Jan-Feb 01 2022;45(1):1-6. doi:10.1097/wnf.0000000000000487
50. Rühl L, Kuramatsu JB, Sembill JA, et al. Amantadine treatment is associated with improved consciousness in patients with non-traumatic brain injury. *J Neurol Neurosurg Psychiatry*. Jun 2022;93(6):582-587. doi:10.1136/jnnp-2021-327408
51. FitzGerald A, Main L, Duff U, et al. Does amantadine maintain function in long-established brain injury? A single case experimental design. *Brain Inj*. Sep 19 2021;35(11):1443-1450. doi:10.1080/02699052.2021.1972341
52. Hadgu RM, Borghol A, Gillard C, et al. Evaluation of Outcomes in Patients Receiving Amantadine to Improve Alertness After Traumatic Brain Injury. *Hosp Pharm*. Oct 2021;56(5):486-494. doi:10.1177/0018578720920803

53. Leclerc AM, Riker RR, Brown CS, et al. Amantadine and Modafinil as Neurostimulants Following Acute Stroke: A Retrospective Study of Intensive Care Unit Patients. *Neurocrit Care*. Feb 2021;34(1):102-111. doi:10.1007/s12028-020-00986-4
54. Alkhachroum A, Eliseyev A, Der-Nigoghossian CA, et al. EEG to detect early recovery of consciousness in amantadine-treated acute brain injury patients. *J Neurol Neurosurg Psychiatry*. Jun 2020;91(6):675-676. doi:10.1136/jnnp-2019-322645
55. Bender Pape TL, Herrold AA, Livengood SL, et al. A Pilot Trial Examining the Merits of Combining Amantadine and Repetitive Transcranial Magnetic Stimulation as an Intervention for Persons With Disordered Consciousness After TBI. *J Head Trauma Rehabil*. Nov-Dec 2020;35(6):371-387. doi:10.1097/htr.0000000000000634
56. Gao Y, Zhang Y, Li Z, Ma L, Yang J. Persistent vegetative state after severe cerebral hemorrhage treated with amantadine: A retrospective controlled study. *Medicine (Baltimore)*. Aug 14 2020;99(33):e21822. doi:10.1097/md.0000000000021822
57. Lee S, Lee HH, Lee Y, Lee J. Additive effect of cerebrolysin and amantadine on disorders of consciousness secondary to acquired brain injury: A retrospective case-control study. *J Rehabil Med*. Feb 27 2020;52(2):jrm00025. doi:10.2340/16501977-2654
58. Zão A, Almeida AF, Beça G, Nunes R. [From Vegetative State to Participation: Amantadine As a Trigger of the Rehabilitation Program]. *Acta Med Port*. Sep 1 2020;33(9):604-609. Do Estado Vegetativo à Participação: A Amantadina Como Potenciadora do Programa de Reabilitação. doi:10.20344/amp.11257
59. Chen X, Tang C, Zhou H, Li Z. Effect of amantadine on vegetative state after traumatic brain injury: a functional magnetic resonance imaging study. *J Int Med Res*. Feb 2019;47(2):1015-1024. doi:10.1177/0300060518814127
60. Lehnerer SM, Scheibe F, Buchert R, Kliesch S, Meisel A. Awakening with amantadine from a persistent vegetative state after subarachnoid haemorrhage. *BMJ Case Rep*. Jul 24 2017;2017doi:10.1136/bcr-2017-220305
61. Estraneo A, Pascarella A, Moretta P, Loreto V, Trojano L. Clinical and electroencephalographic on-off effect of amantadine in chronic non-traumatic minimally conscious state. *J Neurol*. Jun 2015;262(6):1584-6. doi:10.1007/s00415-015-7771-y
62. Avecillas-Chasín JM, Barcia JA. Effect of amantadine in minimally conscious state of non-traumatic etiology. *Acta Neurochir (Wien)*. Jul 2014;156(7):1375-7. doi:10.1007/s00701-014-2077-x
63. Reynolds JC, Rittenberger JC, Callaway CW. Methylphenidate and amantadine to stimulate reawakening in comatose patients resuscitated from cardiac arrest. *Resuscitation*. Jun 2013;84(6):818-24. doi:10.1016/j.resuscitation.2012.11.014
64. Schnakers C, Hustinx R, Vandewalle G, et al. Measuring the effect of amantadine in chronic anoxic minimally conscious state. *J Neurol Neurosurg Psychiatry*. Feb 2008;79(2):225-227. doi:10.1136/jnnp.2007.124099
65. Hughes S, Colantonio A, Santaguida PL, Paton T. Amantadine to enhance readiness for rehabilitation following severe traumatic brain injury. *Brain Inj*. Dec 20 2005;19(14):1197-206. doi:10.1080/02699050500309296
66. Whyte J, Katz D, Long D, et al. Predictors of outcome in prolonged posttraumatic disorders of consciousness and assessment of medication effects: A multicenter study. *Arch Phys Med Rehabil*. Mar 2005;86(3):453-62. doi:10.1016/j.apmr.2004.05.016
67. Saniova B, Drobny M, Kneslova L, Minarik M. The outcome of patients with severe head injuries treated with amantadine sulphate. *J Neural Transm (Vienna)*. Apr 2004;111(4):511-4. doi:10.1007/s00702-004-0112-4

68. Patrick PD, Buck ML, Conaway MR, Blackman JA. The use of dopamine enhancing medications with children in low response states following brain injury. *Brain Inj*. Jun 2003;17(6):497-506. doi:10.1080/0269905031000070279
69. Roy D, Song J, Awad N, Zamudio P. Treatment of unexplained coma and hypokinetic-rigid syndrome in a patient with COVID-19. *BMJ Case Reports*. 2021;14(3):e239781. doi:10.1136/bcr-2020-239781
70. Bancalari E, Rabinstein A, Machiavello F, Desmaison A. Accelerated emergence from a nontraumatic minimally conscious state with levodopa/carbidopa. *Neurol Clin Pract*. Dec 2018;8(6):541-542. doi:10.1212/cpj.0000000000000508
71. Ugoya SO, Akinyemi RO. The place of L-dopa/carbidopa in persistent vegetative state. *Clin Neuropharmacol*. Nov-Dec 2010;33(6):279-84. doi:10.1097/WNF.0b013e3182011070
72. Matsuda W, Komatsu Y, Yanaka K, Matsumura A. Levodopa treatment for patients in persistent vegetative or minimally conscious states. *Neuropsychol Rehabil*. Jul-Sep 2005;15(3-4):414-27. doi:10.1080/09602010443000588
73. Krimchansky BZ, Keren O, Sazbon L, Groswasser Z. Differential time and related appearance of signs, indicating improvement in the state of consciousness in vegetative state traumatic brain injury (VS-TBI) patients after initiation of dopamine treatment. *Brain Inj*. Nov 2004;18(11):1099-105. doi:10.1080/02699050310001646206
74. Matsuda W, Matsumura A, Komatsu Y, Yanaka K, Nose T. Awakenings from persistent vegetative state: report of three cases with parkinsonism and brain stem lesions on MRI. *J Neurol Neurosurg Psychiatry*. Nov 2003;74(11):1571-3. doi:10.1136/jnnp.74.11.1571
75. Sanz LRD, Lejeune N, Szymkowicz E, et al. Apomorphine for prolonged disorders of consciousness: a multimodal open-label study. *eClinicalMedicine*. 2024;78:. doi:10.1016/j.eclinm.2024.102925
76. Fridman EA, Krimchansky BZ, Bonetto M, et al. Continuous subcutaneous apomorphine for severe disorders of consciousness after traumatic brain injury. *Brain Inj*. 2010;24(4):636-41. doi:10.3109/02699051003610433
77. Fridman EA, Calvar J, Bonetto M, et al. Fast awakening from minimally conscious state with apomorphine. *Brain Inj*. Feb 2009;23(2):172-7. doi:10.1080/02699050802649662
78. Kim YW, Shin JC, An YS. Effects of methylphenidate on cerebral glucose metabolism in patients with impaired consciousness after acquired brain injury. *Clin Neuropharmacol*. 2009;32(6):335-9. doi:10.1097/WNF.0b013e3181b40678
79. Munakomi S, Bhattarai B, Mohan Kumar B. Role of bromocriptine in multi-spectral manifestations of traumatic brain injury. *Chin J Traumatol*. Apr 2017;20(2):84-86. doi:10.1016/j.cjtee.2016.04.009
80. Passler MA, Riggs RV. Positive outcomes in traumatic brain injury-vegetative state: patients treated with bromocriptine. *Arch Phys Med Rehabil*. Mar 2001;82(3):311-5. doi:10.1053/apmr.2001.20831
81. Cross DB, Tiu J, Medicherla C, et al. Modafinil in Recovery after Stroke (MIRAS): A Retrospective Study. *J Stroke Cerebrovasc Dis*. Apr 2020;29(4):104645. doi:10.1016/j.jstrokecerebrovasdis.2020.104645
82. Mo Y, Thomas MC, Miano TA, et al. Effect of Modafinil on Cognitive Function in Intensive Care Unit Patients: A Retrospective Cohort Study. *The Journal of Clinical Pharmacology*. 2018;58(2):152-157. doi:10.1002/jcph.1002

83. Formica F, Pozzi M, Avantaggiato P, et al. Disordered Consciousness or Disordered Wakefulness? The Importance of Prolonged Polysomnography for the Diagnosis, Drug Therapy, and Rehabilitation of an Unresponsive Patient With Brain Injury. *J Clin Sleep Med*. Dec 15 2017;13(12):1477-1481. doi:10.5664/jcsm.6854
84. Dhamapurkar SK, Wilson BA, Rose A, Watson P, Shiel A. Does Modafinil improve the level of consciousness for people with a prolonged disorder of consciousness? a retrospective pilot study. *Disabil Rehabil*. Dec 2017;39(26):2633-2639. doi:10.1080/09638288.2016.1236414
85. Rivera VM. Modafinil for the treatment of diminished responsiveness in a patient recovering from brain surgery. *Brain Inj*. Aug 20 2005;19(9):725-7. doi:10.1080/02699050400025091
86. Masotta O, Trojano L, Loreto V, Moretta P, Estraneo A. Selegiline in Patients With Disorder of Consciousness: An Open Pilot Study. *Can J Neurol Sci*. Nov 2018;45(6):688-691. doi:10.1017/cjn.2018.315
87. Li J, Li H, Peng C, Xu W, Chen Q, Liu G. Paradoxical cognitive and language function recovery by zolpidem in a patient with traumatic brain injury: A case report. *Medicine*. 2024;103(28):e38964. doi:10.1097/md.0000000000038964
88. Smack C, Johnson K, Meester N, Shelton L, Singh N. A Patient With Minimally Conscious Syndrome Due to Cerebrovascular Accident Whose Symptoms Resolved With Zolpidem. *Clin Neuropharmacol*. Mar-Apr 01 2024;47(2):59-61. doi:10.1097/wnf.0000000000000587
89. Zhou Y, Altonji KA, Kakkanatt A, Greenwald BD. Speech recovery after single-dose zolpidem in two minimally conscious patients with severe traumatic brain injuries: a case report. *Brain Inj*. Feb 3 2024:1-4. doi:10.1080/02699052.2024.2311342
90. Gao Q, Hao J, Kang X, et al. EEG dynamics induced by zolpidem forecast consciousness evolution in prolonged disorders of consciousness. Article. *Clinical Neurophysiology*. 2023;153:46-56. doi:10.1016/j.clinph.2023.06.012
91. Hao Z, Xia X, Bai Y, Wang Y, Dou W. EEG Evidence Reveals Zolpidem-Related Alterations and Prognostic Value in Disorders of Consciousness. Article. *Front Neurosci*. 2022;16:863016. doi:10.3389/fnins.2022.863016
92. Desmarais LM, Milleville KA, Wagner AK. Postoperative Treatment of Intracranial Hypotension Venous Congestion-Associated Brain Injury with Zolpidem. Article. *American Journal of Physical Medicine and Rehabilitation*. 2021;100(6):E89-E92. doi:10.1097/PHM.0000000000001595
93. Zhang B, O'Brien K, Won W, Li S. A Retrospective Analysis on Clinical Practice-Based Approaches Using Zolpidem and Lorazepam in Disorders of Consciousness. *Brain Sci*. May 29 2021;11(6)doi:10.3390/brainsci11060726
94. Khalili H, Rakhsha A, Ghaedian T, Niakan A, Masoudi N. Application of Brain Perfusion SPECT in the Evaluation of Response to Zolpidem Therapy in Consciousness Disorder Due to Traumatic Brain Injury. *Indian J Nucl Med*. Oct-Dec 2020;35(4):315-320. doi:10.4103/ijnm.IJNM_97_20
95. Sripad P, Rosenberg J, Boers F, et al. Effect of Zolpidem in the Aftermath of Traumatic Brain Injury: An MEG Study. *Case Rep Neurol Med*. 2020;2020:8597062. doi:10.1155/2020/8597062
96. Arnts H, van Erp WS, Boon LI, et al. Awakening after a sleeping pill: Restoring functional brain networks after severe brain injury. *Cortex*. Nov 2020;132:135-146. doi:10.1016/j.cortex.2020.08.011

97. Delargy M, O'Connor R, McCann A, et al. An analysis of the effects of using Zolpidem and an innovative multimodal interdisciplinary team approach in prolonged disorders of consciousness (PDOC). *Brain Inj*. 2019;33(2):242-248. doi:10.1080/02699052.2018.1537008
98. Hahm MH, Woo J. Paradoxical Motor and Cognitive Function Recovery in Response to Zolpidem in a Patient with Hypoxic-ischemic Brain Injury: A Case Report. *Clin Psychopharmacol Neurosci*. Aug 31 2019;17(3):453-457. doi:10.9758/cpn.2019.17.3.453
99. Kondratyeva EA, Sinkin MV, Sharova EV, Laureys S, Kondratyev AN. Zolpidem action during prolonged disorders of consciousness (Case report). Article. *Obshchaya Reanimatologiya*. 2019;15(5):44-60. doi:10.15360/1813-9779-2019-5-44-60
100. Bomalaski MN, Smith SR. Improved Arousal and Motor Function Using Zolpidem in a Patient With Space-Occupying Intracranial Lesions: A Case Report. Article. *PM and R*. 2017;9(8):831-833. doi:10.1016/j.pmrj.2016.12.011
101. Kim C, Kwon BS, Nam KY, Park JW, Lee HJ. Zolpidem-Induced Arousal by Paradoxical GABAergic Stimulation: A Case Report With F-18 Flumazenil Positron Emission Tomography and Single Photon Emission Computed Tomography Study. *Ann Rehabil Med*. 2016;40(1):177-81. doi:10.5535/arm.2016.40.1.177
102. Calabrò RS, Aricò I, De Salvo S, Conti-Nibali V, Bramanti P. Transient awakening from vegetative state: is high-dose zolpidem more effective? *Psychiatry Clin Neurosci*. Feb 2015;69(2):122-3. doi:10.1111/pcn.12215
103. Appu M, Noetzel M. Clinically significant response to zolpidem in disorders of consciousness secondary to anti-N-methyl-d-aspartate receptor encephalitis in a teenager: A case report. Article. *Pediatric Neurology*. 2014;50(3):262-264. doi:10.1016/j.pediatrneurol.2013.11.001
104. Du B, Shan A, Zhang Y, Zhong X, Chen D, Cai K. Zolpidem arouses patients in vegetative state after brain injury: quantitative evaluation and indications. *Am J Med Sci*. Mar 2014;347(3):178-82. doi:10.1097/MAJ.0b013e318287c79c
105. Thonnard M, Gosseries O, Demertzi A, et al. Effect of zolpidem in chronic disorders of consciousness: A prospective open-label study. Article. *Functional Neurology*. 2013;28(4):259-264. doi:https://pubmed.ncbi.nlm.nih.gov/24598393/
106. Rodriguez-Rojas R, Machado C, Alvarez L, et al. Zolpidem induces paradoxical metabolic and vascular changes in a patient with PVS. *Brain Inj*. 2013;27(11):1320-9. doi:10.3109/02699052.2013.794961
107. Williams ST, Conte MM, Goldfine AM, et al. Common resting brain dynamics indicate a possible mechanism underlying zolpidem response in severe brain injury. *Elife*. Nov 19 2013;2:e01157. doi:10.7554/eLife.01157
108. Cohen SI, Duong TT. Increased arousal in a patient with anoxic brain injury after administration of zolpidem. *Am J Phys Med Rehabil*. Mar 2008;87(3):229-31. doi:10.1097/PHM.0b013e318161971b
109. Shames JL, Ring H. Transient reversal of anoxic brain injury-related minimally conscious state after zolpidem administration: a case report. *Arch Phys Med Rehabil*. Feb 2008;89(2):386-8. doi:10.1016/j.apmr.2007.08.137
110. Singh R, McDonald C, Dawson K, et al. Zolpidem in a minimally conscious state. *Brain Inj*. Jan 2008;22(1):103-6. doi:10.1080/02699050701829704
111. Clauss RP, Güldenpfennig WM, Nel HW, Sathekge MM, Venkannagari RR. Extraordinary arousal from semi-comatose state on zolpidem. A case report. *S Afr Med J*. Jan 2000;90(1):68-72.

112. Halbmayer LM, Kofler M, Hitzenberger G, et al. On the recovery of disorders of consciousness under intrathecal baclofen administration for severe spasticity-An observational study. *Brain Behav*. May 2022;12(5):e2566. doi:10.1002/brb3.2566
113. Formisano R, Aloisi M, Contrada M, et al. Late recovery of responsiveness after intra-thecal baclofen pump implantation and the role of diffuse pain and severe spasticity: a case report. *Acta Neurochir (Wien)*. Sep 2019;161(9):1965-1967. doi:10.1007/s00701-019-03994-2
114. Al-Khodairy AT, Wicky G, Nicolo D, Vuadens P. Influence of intrathecal baclofen on the level of consciousness and mental functions after extremely severe traumatic brain injury: Brief report. *Brain Injury*. 2015/03/21 2015;29(4):527-532. doi:10.3109/02699052.2014.984759
115. Margetis K, Korfias SI, Gatzonis S, et al. Intrathecal baclofen associated with improvement of consciousness disorders in spasticity patients. *Neuromodulation*. Oct 2014;17(7):699-704: discussion 704. doi:10.1111/ner.12147
116. Hoarau X, Richer E, Dehail P, Cuny E. Comparison of long-term outcomes of patients with severe traumatic or hypoxic brain injuries treated with intrathecal baclofen therapy for dysautonomia. *Brain Inj*. 2012;26(12):1451-63. doi:10.3109/02699052.2012.694564
117. Shrestha P, Malla H, Pant B, Taira T. Intrathecal baclofen therapy in severe head injury, first time in Nepal, a technique suitable for underdeveloped countries. *Asian J Neurosurg*. Jan 2011;6(1):49-51. doi:10.4103/1793-5482.85638
118. Oyama H, Kito A, Maki H, Hattori K, Tanahashi K. Consciousness recovery induced by intrathecal baclofen administration after subarachnoid hemorrhage -two case reports. *Neurol Med Chir (Tokyo)*. 2010;50(5):386-90. doi:10.2176/nmc.50.386
119. Sarà M, Pistoia F, Mura E, Onorati P, Govoni S. Intrathecal baclofen in patients with persistent vegetative state: 2 hypotheses. *Arch Phys Med Rehabil*. Jul 2009;90(7):1245-9. doi:10.1016/j.apmr.2009.01.012
120. Taira T. Intrathecal administration of GABA agonists in the vegetative state. *Prog Brain Res*. 2009;177:317-28. doi:10.1016/s0079-6123(09)17721-x
121. Carboncini MC, Piarulli A, Virgillito A, et al. A case of post-traumatic minimally conscious state reversed by midazolam: Clinical aspects and neurophysiological correlates. *Restor Neurol Neurosci*. 2014;32(6):767-87. doi:10.3233/rnn-140426
122. Reinhard DL, Whyte J, Sandel ME. Improved arousal and initiation following tricyclic antidepressant use in severe brain injury. *Arch Phys Med Rehabil*. Jan 1996;77(1):80-3. doi:10.1016/s0003-9993(96)90225-7
123. Hérault C, André-Obadia N, Naccache L, Luauté J. Potential therapeutic effect of Lamotrigine in disorders of consciousness after severe traumatic brain injury: A series of 4 cases. *Annals of Physical and Rehabilitation Medicine*. 2024/11/01/ 2024;67(8):101868. doi:10.1016/j.rehab.2024.101868
124. Ng M, Ali S, Sue J, et al. Beneficial cognitive effect of lamotrigine in severe acquired brain injury: A case report. *Interdisciplinary Neurosurgery*. 2021/09/01/ 2021;25:101186. doi:10.1016/j.inat.2021.101186
125. Bagnato S, Boccagni C, Sant'angelo A, Galardi G. A range of antiepileptic drugs do not affect the recovery of consciousness in vegetative and minimally conscious states. *Epilepsy Behav*. May 2013;27(2):365-70. doi:10.1016/j.yebeh.2013.02.005
126. Showalter PE, Kimmel DN. Stimulating consciousness and cognition following severe brain injury: a new potential clinical use for lamotrigine. *Brain Inj*. Nov 2000;14(11):997-1001. doi:10.1080/02699050050191931
